# Synthesis, structure–activity relationships, and SARS-CoV-2 antiviral activity of 3,5-disubstituted isothiazolo[4,3-*b*]pyridines as PIKfyve inhibitors

**DOI:** 10.3389/fchem.2026.1777981

**Published:** 2026-05-08

**Authors:** Ling-Jie Gao, Demian Kalebic, Chieh Wen Lo, Aakriti Gangwal, Do Hoang Nhu Tran, Jef Rozenski, Dominique Schols, Mathy Froeyen, Wim Dehaen, Shirit Einav, Steven De Jonghe

**Affiliations:** 1 Laboratory of Medicinal Chemistry, Department of Pharmaceutical and Pharmacological Sciences, Rega Institute for Medical Research, KU Leuven, Leuven, Belgium; 2 Sustainable Chemistry for Metals and Molecules, Department of Chemistry, KU Leuven, Leuven, Belgium; 3 Department of Medicine, Division of Infectious Diseases and Geographic Medicine, and Department of Microbiology and Immunology, Stanford University School of Medicine, Stanford, CA, United States; 4 Molecular, Structural and Translational Virology Research Group, Department of Microbiology, Immunology and Transplantation, Rega Institute for Medical Research, KU Leuven, Leuven, Belgium; 5 Biohub, San Francisco, CA, United States

**Keywords:** antiviral, isothiazolo[4,3-b]pyridine, PIKfyve, SARS-CoV-2, structure–activity relationship

## Abstract

**Introduction:**

3-Alkynyl-6-aryl-isothiazolo[4,3-b]pyridines have previously been shown to be potent inhibitors of the lipid kinase FYVE finger-containing phosphoinositide kinase (PIKfyve), displaying broad-spectrum antiviral activity.

**Methods:**

To further study their structure–activity relationship (SAR), an efficient synthesis toward 3- bromo-5-chloro-isothiazolo[4,3-b]pyridine was established. It allowed to introduce structural modifications at positions 3 and 5 by palladium-catalyzed cross-coupling reactions and nucleophilic aromatic substitutions.

**Results and discussion:**

It led to the generation of a focused library of 3,5-disubstituted isothiazolo[4,3-b]pyridines. Several derivatives exhibited potent PIKfyve inhibition (in the low nM range) in a biochemical assay and antiviral activity against severe acute respiratory syndrome coronavirus 2 (SARS-CoV-2) (in the low μM range). To gain an insight in their binding mode, molecular modeling was applied, indicating that these 3,5- disubstituted isothiazolo[4,3-b]pyridines bind to the ATP-binding site of PIKfyve, although with a different binding mode from that of the 3,6- disubstituted isothiazolo[4,3-b]pyridines.

## 1 Introduction

Kinases are a large and diverse class of proteins, with more than 500 members, that constitute approximately 2% of the human proteome (Anderson et al., 2023). Kinases perform phosphorylation reactions by transferring the γ phosphate of adenosine triphosphate (ATP) onto the hydroxyl groups of different substrates, including proteins and lipids. Historically, kinases that phosphorylate serine/threonine and tyrosine residues of proteins have been the focus of basic research and therapeutic investigations (Kanev et al., 2019). In contrast, research on lipid kinases has been lagging behind. Well-known examples of the lipid kinase family are phosphoinositide (PI) kinases (Llorente et al., 2023), diacylglycerol (DAG) kinases (Sakane et al., 2007), and sphingosine kinases (Hatoum et al., 2017).

The FYVE finger-containing phosphoinositide kinase (PIKfyve) is a PI kinase that catalyzes the production of phosphatidylinositol 3,5-bisphosphate (PI(3,5)P2) from phosphatidylinositol 3-phosphate (PI3P). PIKfyve also produces the main pool of PI5P in the cell, which is primarily generated indirectly through the dephosphorylation of PI(3,5)P2 by lipid phosphatases, although a small pool of PI5P may be generated by PIKfyve’s direct phosphorylation of PI to generate PI5P. PIKfyve is primarily localized at endosomal membranes, where it plays a critical role in regulating membrane homeostasis, endosomal trafficking, and autophagy in the endosomal and lysosomal systems (Burke et al., 2023).

**FIGURE 1:**
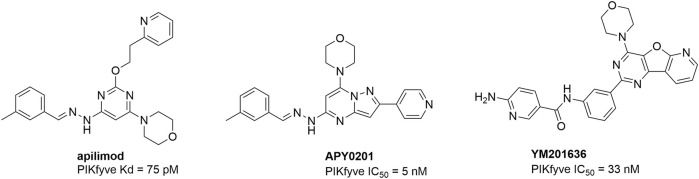
Known PIKfyve inhibitors with antiviral or antitumoral activity.

PIKfyve inhibition has been pursued as a therapeutic strategy in different disease areas. PIKfyve inhibition mediates autophagy dysfunction by blocking autophagic flux and exhibits antitumor efficacy in various cancer types. Apilimod (Figure 1) is a highly potent PIKfyve inhibitor that has been studied extensively for its antitumoral properties (Gayle et al., 2017). APY0201 and YM201636 (Figure 1) are two other PIKfyve inhibitors that have shown potent antitumoral activity when evaluated against 25 human multiple myeloma and 15 non-Hodgkin lymphoma cell lines (Campos et al., 2020).

Endocytosis is a mechanism by which viruses from various unrelated families enter their host cell. As PIKfyve is involved in virus trafficking via this endocytic pathway, PIKfyve inhibition has been explored as a possible broad-spectrum antiviral strategy. Apilimod blocks the entry of filoviruses, such as the Ebola virus (EBOV) and Marburg virus (MARV), in various cell lines and primary human macrophages (Nelson et al., 2017) and exhibits activity against several respiratory viruses, including severe acute respiratory syndrome coronavirus 2 (SARS-CoV-2), influenza virus, and respiratory syncytial virus (Baker et al., 2023). YM201636 inhibits the replication of several other RNA viruses, such as enterovirus 71, coxsackievirus B3, poliovirus 1, echovirus 11, Zika virus, and vesicular stomatitis virus (Luo et al., 2023).

**FIGURE 2:**
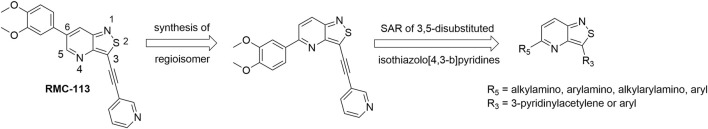
Medicinal chemistry strategy.

We recently reported the discovery of **RMC-113** as a highly potent and selective PIKfyve inhibitor, based on a chemotype, which is completely different from other PIKfyve inhibitors (Karim et al., 2025). **RMC-113** is a 3-alkynyl-6-aryl isothiazolo[4,3-*b*]pyridine analog (Figure 2), displaying antiviral activity against SARS-CoV-2, the vaccine strain of VEEV (TC-83), the dengue virus, EBOV, and MARV. Previous structure–activity relationship (SAR) studies predominantly focused on the introduction of different (hetero)aryl groups at position 6 of the scaffold and various alkynyl moieties at position 3 of the isothiazolo[4,3-*b*]pyridine core (Kalebic et al., 2025).

To expand chemical space and to study structural features that are required for PIKfyve inhibition, in this study, 3,4-dimethoxyphenyl was moved from position 6 to position 5 of the central core (Figure 2). This structural change was driven by the fact that a similar strategy has been successfully applied by us before, in the context of a drug discovery program focusing on isothiazolo[4,3-*b*]pyridines as inhibitors of cyclin G-associated kinase (Martinez-Gualda et al., 2020). Moreover, position 5 allows for the easy introduction of a broad repertoire of substituents (by nucleophilic aromatic substitutions and palladium-catalyzed reactions), whereas position 6 is restricted to structural variation by cross-coupling reactions. Hence, this is not a structure-based drug design approach, but rather an empirical exploration of the SAR. As this regioisomeric 3-alkynyl-5-aryl-substituted isothiazolo[4,3-*b*]pyridine analog was endowed with potent PIKfyve inhibition, it was selected as the lead compound for further SAR studies. Positions 3 and 5 of the isothiazolo[4,3-*b*]pyridine scaffold were both subjected to nucleophilic aromatic substitutions and palladium-catalyzed cross-coupling reactions, yielding a library of 3,5-disubstituted isothiazolo[4,3-*b*]pyridines.

## 2 Results and discussion

### 2.1 Chemistry

**SCHEME 1:**
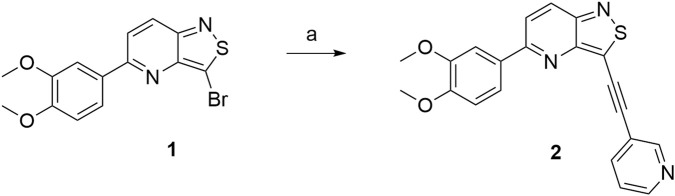
Synthesis of the regioisomer 2. *Reagents and conditions*: a) 3-ethynylpyridine, CuI, Pd(PPh_3_)_2_Cl_2_, Et_3_N, and THF, 30 °C, overnight.

Starting from the known 3-bromo-5-(3,4-dimethoxyphenyl)isothiazolo[4,3-*b*]pyridine **1** (Martinez-Gualda et al., 2020), a Sonogashira reaction with 3-ethynylpyridine allowed the preparation of compound **2** (Scheme 1).

However, this chemistry was not ideal for an efficient SAR exploration, as structural variety at position 5 is implemented early on in the synthetic sequence. For convenient introduction of modifications at positions 3 and 5 of the isothiazolo[4,3-*b*]pyridine scaffold, the availability of a 3,5-dihalo-isothiazolo[4,3-*b*]pyridine building block was preferred.

**SCHEME 2:**
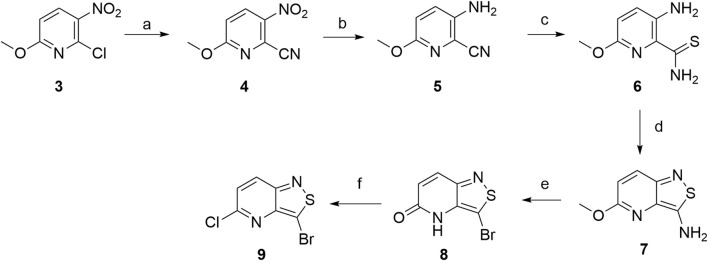
Initial synthetic route to 3-bromo-5-chloroisothiazolo[4,3-*b*]pyridine 9. *Reagents and conditions*: a) CuCN and DMF, 100 °C, 72 h; b) Fe and AcOH, r.t., 1 h; c) P_4_S_10_ and EtOH, 75 °C, overnight; d) 35% aq. H_2_O_2_, r.t., overnight; e) NaNO_2_, aq. HBr, and CuBr, 0 °C to r.t. overnight then aq. HBr, 100 °C, 2 h; f) POCl_3_ and DCE, 80 °C, 6 h.

Hence, 2-chloro-6-methoxy-3-nitropyridine **3** was selected as the starting material (Scheme 2). A Rosenmund–von Braun reaction with copper(I) cyanide in DMF yielded 6-methoxy-3-nitropicolinonitrile **4** in 60% yield based on a known procedure (Piersanti et al., 2007), although the reaction was run for 2 days instead of the reported 6 h. The subsequent reduction using stannous chloride in ethanol yielded 3-amino-6-methoxypicolinamide as the major compound, alongside a minor amount of the desired 3-amino-6-methoxypicolinonitrile **5**. In contrast, treatment of **4** with iron in acetic acid afforded the 3-amino-6-methoxypicolinonitrile **5** in good yield. Thionation using tetraphosphorus decasulfide furnished thioamide **6**, which was subjected to an oxidative cyclization yielding isothiazolo[4,3-*b*]pyridine **7** (Martinez-Gualda et al., 2020). A Sandmeyer reaction (using NaNO_2_ and CuBr in HBr) converted the 3-amino group into a bromine, with concomitant hydrolysis of the methoxy group, yielding 3-bromo-5-hydroxy-isothiazolo[4,3-*b*]pyridine **8**. Subsequent chlorination with phosphorus oxychloride furnished 3-bromo-5-chloroisothiazolo[4,3-*b*]pyridine **9**.

**SCHEME 3:**
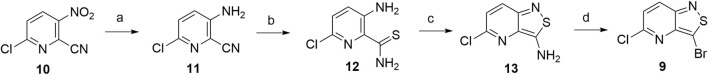
Improved synthesis of 3-bromo-5-chloroisothiazolo[4,3-*b*]pyridine 9. *Reagents and conditions*: a) Fe and HOAc, r.t., 2 h; b) P_2_S_5_ and EtOH, 75 °C, 6 h, 82% from 10; c) 30% H_2_O_2_ and MeOH, 0 °C to rt, 2 h; d) NaNO_2_, HBr, and CuBr, 0 °C to r.t., 8 h, 79% from 12.

To access key building block **9** in a more efficient way, commercially available 6-chloro-3-nitropicolinonitrile **10** was used as the alternative starting material (Scheme 3). Treatment with iron powder in acetic acid afforded 3-amino-6-chloropicolinonitrile **11** in good yield. The crude product was contaminated with a minor side product that was identified as 3-amino-6-chloropicolinamide. Although both compounds could be separated using silica gel column chromatography, this was not required. Indeed, the reaction of crude **11** (contaminated with 3-amino-6-chloropicolinamide) with tetraphosphorus decasulfide in ethanol at reflux furnished 3-amino-6-chloropyridine-2-carbothioamide **12**. As 3-amino-6-chloropicolinamide did not undergo any reaction with tetraphosphorus decasulfide, it was easily separated using silica gel flash chromatography from the desired product **12** at this stage. The yield over these two steps was 82%. The reaction of 3-amino-6-chloropyridine-2-carbothioamide **12** with hydrogen peroxide in methanol allowed the construction of the isothiazole moiety, and 3-amino-5-chloroisothiazolo[4,3-*b*]pyridine **13** was formed almost quantitatively. Finally, a Sandmeyer reaction produced 3-bromo-5-chloroisothiazolo[4,3-*b*]pyridine **9** in good yield. The yield over the last two steps was 79%, and the total overall yield over four steps was as high as 65%.

**SCHEME 4:**
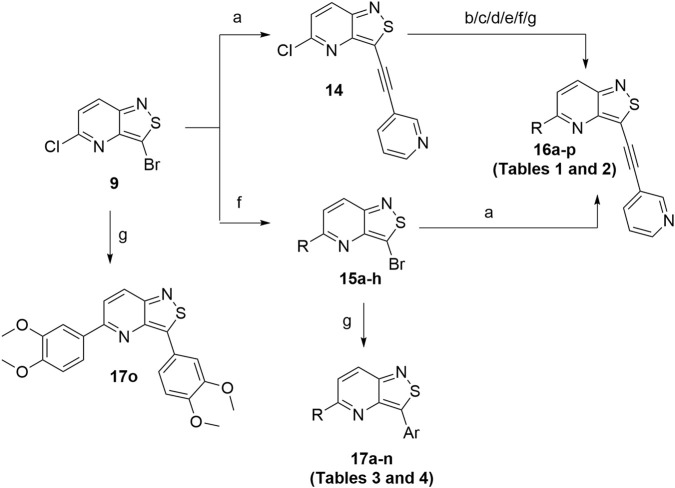
Structural variation at positions 3 and 5 of the isothiazolo[4,3-*b*]pyridine scaffold. *Reagents and conditions*: a) 3-ethynylpyridine, TEA, Pd(PPh_3_)_2_Cl_2_, CuI, and THF, r.t., overnight; b) alkyne, TEA, Pd(PPh_3_)_2_Cl_2_, CuI, and THF, 30 °C, overnight; c) 3,4-dimethoxybenzylamine and EtOH, 75 °C, overnight; d) alkylamine, K_2_CO_3_, and dioxane, 80 °C, 1–4 h; e) arylmethanamine and dioxane, reflux, 4–12 h; f) aniline, HCl (1 drop) and dioxane, reflux, 4–12 h; g) arylboronic acid, Pd(PPh_3_)_4_, K_2_CO_3_, and dioxane/H_2_O (4:1), 90 °C, 1–4 h.

This 3-bromo-5-chloro-isothiazolo[4,3-*b*]pyridine **9** is a highly versatile intermediate for the introduction of structural variety (Scheme 4). A Sonogashira reaction with 3-ethynylpyridine regioselectively afforded 5-chloro-3-(pyridine-3-ylethynyl)isothiazolo[4,3-*b*]pyridine **14**. Alternatively, nucleophilic aromatic substitutions of compound **9** with various amines led to 5-substituted-3-bromo-isothiazolo[4,3-*b*]pyridines **15a**-**h**.

Chlorine of intermediate **14** was an easy handle to introduce structural modifications at position 5 of the isothiazolo[4,3-*b*]pyridine skeleton. A Sonogashira cross-coupling with 3,4-dimethoxyphenylacetylene [prepared according to a known procedure (Li et al., 2020)] yielded bisacetylenic analog **16a**, whereas nucleophilic aromatic substitutions with a variety of amines yielded target compounds **16b**–**n**. Palladium-catalyzed Suzuki reactions with various arylboronic acids yielded compounds **16o**–**p**.

3-Bromoisothiazolo[4,3-*b*]pyridines **15a**–**h** were used as starting materials for a series of Suzuki reactions with different arylboronic acids, yielding 3-aryl-5-substituted isothiazolo[4,3-*b*]pyridines **17a**-**n**. Finally, when 3-chloro-5-bromoisothiazolo[4,3-*b*]pyridine **9** was subjected to standard Suzuki reaction conditions, but using an excess of 3,4-dimethoxyphenylboronic acid, compound **17o** was obtained.

### 2.2 PIKfyve inhibition

The newly synthesized isothiazolo[4,3-*b*]pyridines were evaluated as potential PIKfyve inhibitors. Apilimod, a well-known PIKfyve inhibitor, was taken along as the positive control, yielding an IC_50_ value of 0.79 nM, in agreement with literature data. **RMC-113**, the lead compound, was included as the reference compound.

To demonstrate the potential of 3,5-disubstituted isothiazolo[4,3-*b*]pyridines as PIKfyve inhibitors, compound **2**, the regioisomer of the lead compound **RMC-113**, in which the 3,4-dimethoxyphenyl residue is switched from position 6 to position 5 of the scaffold, was first investigated (Table 1). As compound **2** was equally active as the PIKfyve inhibitor as **RMC-113** (IC_50_ values of 10 nM and 8 nM, respectively), the SAR at position 5 was studied more extensively. The rationale for selection of certain substituents on the central isothiazolo[4,3-*b*]pyridine core was driven by the commercial availability of building blocks, synthetic feasibility, and drug-likeness of the groups. Several linkers between the 3,4-dimethoxyphenyl moiety and the isothiazolo[4,3-*b*]pyridine scaffold were inserted. The insertion of an ethynyl (compound **16a**), an amino (compound **16b**), or an aminomethylene (compound **16c**) linker afforded compounds displaying potent PIKfyve inhibition with IC_50_ values between 9 and 38 nM. Overall, PIKfyve inhibition tolerated various linkers between the dimethoxyphenyl moiety and the central isothiazolo[4,3-*b*]pyridine skeleton.

**TABLE 1:** PIKfyve inhibition of 5-substituted 3-(pyridin-3-ylethynyl)isothiazolo[4,3-*b*]pyridines.

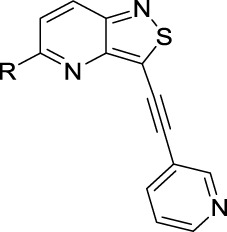		
Cmpd	R	IC_50_ (µM)
**Apilimod**	See Figure 1	0.00079
**RMC-113**	See Figure 2	0.008
**2**	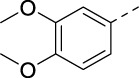	0.013
**16a**	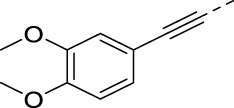	0.009
**16b**	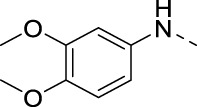	0.010
**16c**	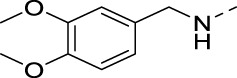	0.038

The commercial availability of a wide range of amines and their convenient introduction at position 5 of the isothiazolo[4,3-*b*]pyridine scaffold allowed to expand structural diversity (Table 2). Moreover, as we found that the two methoxy groups were metabolically labile (unpublished data), chemistry was focused on finding an alternative substitution pattern at position 5 of the scaffold. The insertion of (cyclo)aliphatic amines afforded compounds **16d**–**g**. These compounds have a reduced aromatic count, which is beneficial in terms of drug-likeness, but, unfortunately, they displayed a clearly reduced PIKfyve inhibition when compared to compounds **16a**–**c**, suggesting that aromatic groups at this position are required for potent PIKfyve inhibition. The known beneficial effect of fluorine on metabolic stability, combined with the requirement for an aryl group for potent PIKfyve inhibition, encouraged us to introduce fluorinated anilino and benzylamino groups, yielding compounds **16h**–**i** and **16j**–**k**, respectively, all displaying very potent PIKfyve inhibition, with IC_50_ values in the 15 nM range. As heteroaromatics usually exhibited an improved aqueous solubility when compared to their phenyl counterparts, 2-, 3- or 4-(aminomethyl)pyridinyl moieties were also inserted furnishing compounds **16l**–**n** that displayed potent PIKfyve inhibition, with IC_50_ values ranging from 13 to 22 nM. The presence of a halogen at position 5 of key intermediate 3-bromo-5-chloroisothiazolo[4,3-*b*]pyridine **9** allowed for the introduction of various aryl groups through Suzuki cross-coupling reactions. Unfortunately, halogenated phenyl moieties (such as in compounds **16o**–**p**) led to a strongly reduced PIKfyve inhibition, when compared to 3,4-dimethoxyphenyl analog **2**. Overall, the insertion of (hetero)arylamino groups at position 5 afforded more potent PIKfyve inhibitors than aryl and (cyclo)aliphatic amino moieties.

**TABLE 2:** PIKfyve inhibition of 5-amino-3-(pyridin-3-ylethynyl)isothiazolo[4,3-*b*]pyridines.

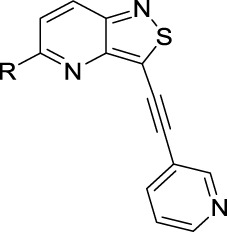		
Cmpd#	R	IC_50_ (µM)
**16d**	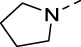	0.287
**16e**	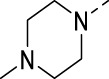	1.180
**16f**	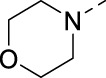	0.485
**16g**	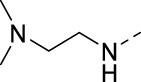	0.426
**16h**	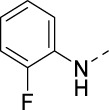	0.017
**16i**	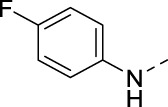	0.013
**16j**	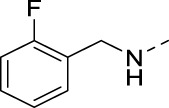	0.015
**16k**	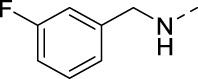	0.016
**16l**	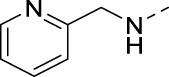	0.022
**16m**	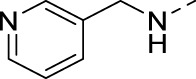	0.013
**16n**	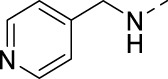	0.019
**16o**	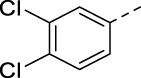	>10
**16p**	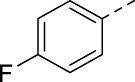	0.18

The 3-(aminomethyl)pyridinyl analog **16m** is equipotent to the 3,4-dimethoxyphenyl-containing congeners **2** and **16a**–**c**. As compound **16m** lacks methoxy groups (which is beneficial in terms of metabolic stability) and possesses a pyridinyl moiety (which is advantageous for aqueous solubility), it was selected for further SAR studies. The 3-(aminomethyl)pyridinyl group was fixed at position 5, and various other groups were inserted at position 3 (Table 3). It was previously shown that the triple bond of **RMC-113** is susceptible to nucleophilic attack (Kalebic et al., 2025), and the presence of such an electrophilic acetylene moiety was deemed as obsolete. Moreover, for 3,6-disubstituted isothiazolo[4,3-*b*]pyridines, it was demonstrated that PIKfvye inhibition tolerated an aryl group at position 3 (Kalebic et al., 2025). In addition, the availability of key intermediate **9** enables easy access to Suzuki-coupling-derived compounds. The insertion of a 3,4-dimethoxyphenyl moiety was optimal for PIKfyve inhibition, yielding compound **17a** with an IC_50_ value of 80 nM. Substitution of one of the methoxy groups by a fluorine (compounds **17b**–**c**) led to a decrease in PIKfyve inhibitory activity. This loss of PIKfyve inhibition was even more pronounced when both methoxy groups were replaced by fluorine as in derivative **17d** (IC_50_ = 1 µM). Substitution of the 4-methoxy group by a chlorine yielded compound **17e**, which was two-fold less active than the parent 3,4-dimethoxyphenyl congener **17a**. In contrast, the 3-chloro-4-methoxyphenyl congener **17f** was equally active as **17a**. This suggested that the 4-methoxy group was more important than the 3-methoxy functionality for PIKfyve inhibition. Therefore, the 4-methoxyphenyl analog **17g** was prepared, which was less potent than its 3,4-dimethoxyphenyl counterpart. The 4-fluorophenyl derivative **17h** showed a similar profile as compound **17g**. Finally, to mimic better the 3,4-dimethoxyphenyl moiety, the benzo[d][1,3]dioxole analog **17i**, as a close mimic of the 3,4-dimethoxyphenyl derivative **17a**, was four-fold less active as the PIKfyve inhibitor than **17a**. Although the 3,4-dimethoxyphenyl residue is not the preferred substituent for pharmacokinetic reasons, it was shown to be the optimal surrogate for the pyridinylacetylene moiety at position 3 of the isothiazolo[4,3-*b*]pyridine scaffold in terms of PIKfyve inhibition. Therefore, in a final round of optimization, the 3,4-dimethoxyphenyl moiety was fixed, and various amines were introduced at position 5 (Table 4). Fluorinated anilino and benzylamino groups at position 5 in the 3-(3-pyridinylacetylene) series (Table 2) were associated with potent PIKfyve inhibition; therefore, a similar strategy was pursued in the 3-(3,4-dimethoxyphenyl) series, yielding compounds **17j**–**m**. Both the 2-fluoroanilino (compound **17j**) and 3,4-difluoroanilino (compound **17l**) analogs are endowed with similar PIKfyve inhibitory activity as the parent compound **17a**. Moreover, in this series, position 5 tolerates various structural modifications. As a 4-(4-methylpiperazin-1-yl)anilino group (compound **17n**) maintained potent PIKfyve inhibition (IC_50_ = 40 nM), this position seemed ideally suited to modulate physicochemical properties, for example, for the insertion of aqueous solubility-enhancing groups. Finally, the bis(3,4-dimethoxyphenyl) analog **17o** led to potent PIKfyve inhibition (IC_50_ = 20 nM), although the presence of four methoxy groups makes this compounds less appealing in terms of drug-likeness.

**TABLE 3:** PIKfyve inhibition of 5-(3-(aminomethyl)pyridinyl)isothiazolo[4,3-*b*]pyridines.

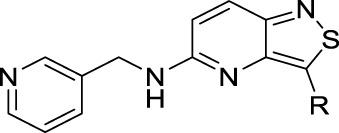		
Cmpd	R	IC_50_ (µM)
**17a**	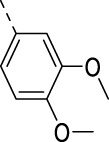	0.08
**17b**	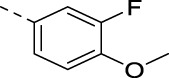	0.13
**17c**	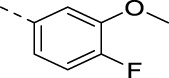	0.38
**17d**	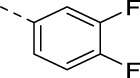	1.06
**17e**	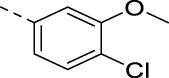	0.16
**17f**	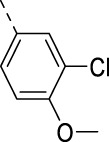	0.07
**17g**	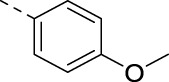	0.14
**17h**	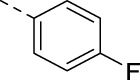	0.92
**17i**	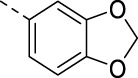	0.31

**TABLE 4:** PIKfyve inhibition of 5-substituted 3-(3,4-dimethoxyphenyl)isothiazolo[4,3-*b*]pyridines.

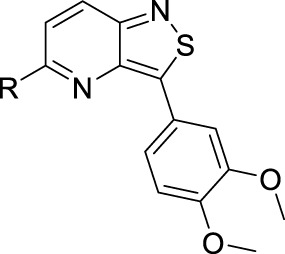		
Cmpd	R	IC_50_ (µM)
**17a**	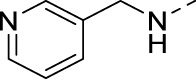	0.08
**17j**	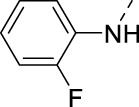	0.08
**17k**	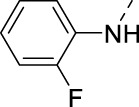	0.57
**17l**	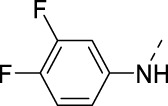	0.09
**17m**	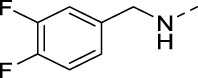	0.12
**17n**	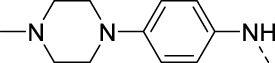	0.04
**17o**	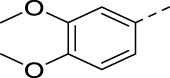	0.02

### 2.3 Molecular modeling

**FIGURE 3:**
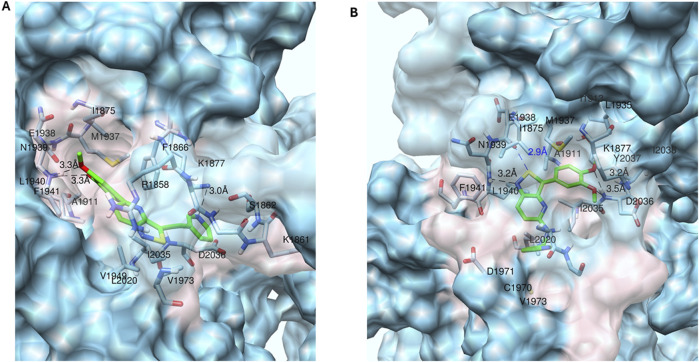
Putative binding modes of compounds 2 and 17a into the PIKfyve ATP binding pocket. (A) Snapshot during MD simulation of compound 2 (green carbons). Hydrogen bonds from the pyridine nitrogen to Lys1877.NZ and from the methoxy oxygens to Leu1940.NH are shown. Additional stabilization is obtained by van der Waals contacts with all labeled residues (in pink). (B) Snapshot during the MD of compound 17a (green carbons). Hydrogen bonds from the nitrogen of the isothiazole to Lys1940.NH and from the 2 methoxy oxygens to Asp2036.NH are shown. A chalcogen bond between the sulfur of compound 17a and the backbone oxygen in Asn1938 is shown. Additional stabilization is obtained by van der Waals contacts with all labeled residues (in pink).

As mentioned above, the design of new analogs and the SAR exploration were largely empirically driven and did not follow a structure-based drug design approach. However, to rationalize the experimentally determined SAR and to shed light on the binding mode of the 3,5-disubstituted isothiazolo[4,3-*b*]pyridines in the ATP binding pocket of PIKfyve, molecular modeling was applied. Compounds **2** and **17a** were selected as representative examples to elucidate their putative binding sites (Figure 3).

Similarly to **RMC-113**, compound **2** also interacted with Leu1940.NH in the hinge region of PIKfyve. For **RMC-113**, a hydrogen bond between Leu1940.NH and the nitrogen of the isothiazole moiety was observed (Karim et al., 2025), whereas in compound **2**, this interaction occurred via the two oxygen atoms of the dimethoxyphenyl moiety (Figure 3A). In contrast, the methoxy groups of **RMC-113** show no specific interactions, except for van der Waals contacts. Hence, moving the 3,4-dimethoxyphenyl group from position 6 (as in **RMC-113**) to position 5 (as in compound **2**) had a drastic effect on the binding orientation. This explained why compounds **16o**–**p**, both lacking the methoxy groups on the phenyl ring, were devoid of potent PIKfyve inhibition. Changing the chemical nature of the side chain at position 5 affected the activity of the compounds as compounds **16d**–**g** are clearly less active, whereas compounds **16h**–**n** are potent PIKfyve inhibitors. Moreover, an additional hydrogen bond between the nitrogen of the pyridinylacetylene group of compound **2** and the side chain of Lys1877 was noticed, similar to what was observed for **RMC-113**.

Compound **17a** restores the original hinge interaction of **RMC-113** as the nitrogen of the isothiazole moiety of compound **17a** is hydrogen-bound to Leu1940.NH of the hinge region of PIKfyve, which is identical to what was observed previously for **RMC-113** (Karim et al., 2025). However, the orientation of compound **17a** in the ATP binding site of PIKfyve does not match with **RMC-113** because of the altered position and properties of the substituents at positions 3 and 5 of the central core. Hydrogen bonds from the two methoxy oxygen atoms to the backbone NH of Asp2036 are formed. Hence, removal of one or both methoxy groups (as in compounds **17b**–**i**) led to a decrease in PIKfyve inhibition. Structural modifications of the substituent at position 5 of compound **17a** influenced the activity, with potent PIKfvye inhibitors **17j**, **17l**, and **17o**, along with the less active compounds **17k** and **17m**. In addition, there is also a chalcogen bond between the sulfur atom of compound **17a** and the backbone oxygen of Asn1938 (Figure 3B).

### 2.4 Kinase selectivity

**FIGURE 4:**
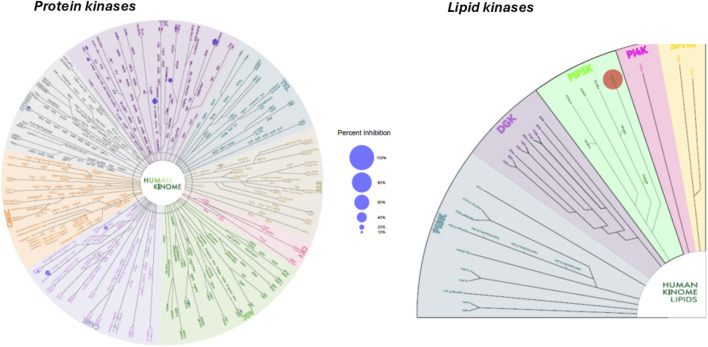
Kinome tree of compound 16i. Blue circles indicate kinases inhibited, whereas the red circle indicates the kinase of interest (i.e., PIKfyve).

Several of the newly synthesized 3,5-disubstituted isothiazolo[4,3-*b*]pyridines showed potent PIKfyve inhibition. As the ATP binding pocket of kinases is structurally conserved over the human kinome, the design and synthesis of selective kinase inhibitors are a challenging endeavor. To shed light on the selectivity of this compound class, a kinase selectivity assay was performed against a 70-membered kinase panel consisting of 66 protein kinases and 4 lipid kinases (the exact list of kinases is available in Supporting Information). Compound **16i** was selected as a representative example because of its potent PIKfyve inhibition (IC_50_ = 13 nM), its promising antiviral activity against SARS-CoV-2 (EC_50_ value of 0.18 µM), and its lack of cytotoxicity (CC_50_ > 20 µM). Compound 1**6i** was screened against these 70 kinases at a single concentration of 10 μM. As can be derived from the kinase tree (Figure 4), none of the other kinases were targeted by compound **16i**. Only a few kinases (i.e., DAPK2 and RET) were inhibited more than 20% by compound **16i** at 10 μM, making it a potent and selective PIKfyve inhibitor.

### 2.5 Antiviral evaluation

Although PIKfyve inhibition is associated with broad-spectrum antiviral activity, in the current study, antiviral profiling was limited to SARS-CoV-2. A number of anti-SARS-CoV-2 agents received marketing approval, each one with its own drawbacks (Li et al., 2023). The nucleoside analog remdesivir is not orally bioavailable and needs intravenous administration. Molnupiravir is another, orally bioavailable, viral polymerase inhibitor for which there are ongoing uncertainties about its long-term safety. Nirmatrelvir is a SARS-CoV-2 main protease inhibitor. It is orally administered with ritonavir and hence has significant drug–drug interaction concerns.

The most potent PIKfyve inhibitors (IC_50_ < 80 nM) were evaluated for their ability to inhibit the replication of wild-type SARS-CoV-2, expressing the reporter protein nanoluciferase (nLuc), in human lung epithelial (Calu-3) cells. In parallel, cell viability in the presence of the compound was determined via an alamarBlue assay (Table 5). **RMC-113**, the lead compound, was included as the reference, yielding antiviral (EC_50_ = 1.01 µM) and cytotoxicity (CC_50_ > 20 µM) values in agreement with previous data (Karim et al., 2025). The newly synthesized 3,5-disubstituted isothiazolo[4,3-*b*]pyridines exhibited antiviral potency compared to or exceeding that of the parent compound **RMC-113**, with EC_50_ values ranging from 0.10 to 1.18 µM. Although a subset (compounds **16l**, **17j**, and **17o**) demonstrated varying degrees of cytotoxicity (CC_50_ = 2.3–11.7 µM), the majority showed no detectable toxicity for the Calu-3 cells (CC_50_ > 20 µM). Compound **16i** displayed potent antiviral activity (EC_50_ = 0.18 µM) without measurable cytotoxicity (CC_50_ > 20 µM), highlighting its promise as a lead candidate. Although these isothiazolo[4,3-*b*]pyridines are endowed with potent PIKfyve inhibition (IC_50_ values in the 8–80 nM range), their antiviral activity is much less pronounced, and these compounds are up to 100-fold less potent as antiviral agents than as PIKfyve inhibitors. The exact reasons for this discrepancy are not clear, but as the antiviral efficacy is the final result of various factors (such as target affinity, solubility, permeability, efflux, and stability), it is usually more difficult to achieve potent activity in a cellular assay. Moreover, although a kinase selectivity screening revealed that compound **16i** is highly selective when evaluated against a panel of 70 kinases, it cannot be excluded that this compound class interacts with other targets (including other kinases). These off-target effects of some of the analogs might also be responsible for the cytotoxicity that is observed for some derivatives (e.g., compounds **17j** and **17o**).

**TABLE 5:** Anti-SARS-CoV-2 activity of selected isothiazolo[4,3-*b*]pyridines.

Cmpd	EC_50_ (µM)^a^	CC_50_ (µM)^b^
**RMC-113**	1.01 ± 0.04	>20
**16h**	0.32 ± 0.04	>20
**16i**	0.18 ± 0.08	>20
**16j**	0.43 ± 0.04	>20
**16k**	0.25 ± 0.09	>20
**16l**	0.33 ± 0.10	11.70 ± 1.44
**16m**	0.90 ± 0.13	>20
**17f**	0.22 ± 0.11	>20
**17j**	0.10 ± 0.00	2.87 ± 0.01
**17n**	1.18 ± 0.03	>20
**17o**	0.10 ± 0.02	2.30 ± 0.47

Data are averages ±standard deviation of two independent experiments.

^a^ 50% effective concentration, or concentration required to inhibit SARS-CoV-2 replication in Calu3 cells by 50%.

^b^ 50% cytotoxic concentration, or concentration required to reduce viability of Calu3 cells by 50%.

## 3 Conclusion

The isothiazolo[4,3-*b*]pyridine skeleton is a scaffold that has been associated with potent PIKfyve inhibition. The SAR of 3,6-disubstituted isothiazolo[4,3-b]pyridines as PIKfyve inhibitors has been studied previously. In this study, starting from 3-bromo-5-chloroisothiazolo[4,3-*b*]pyridine, nucleophilic aromatic substitutions and palladium-catalyzed cross-couplings allowed to introduce broad structural variety, yielding a focused library of 3,5-disubstituted isothiazolo[4,3-*b*]pyridines. Several analogs that were endowed with potent PIKfyve inhibition (low nM activity) emerged. Molecular modeling indicated that similarly to 3,6-disubstituted isothiazolo[4,3-*b*]pyridines, these analogs also engage the ATP-binding pocket of PIKfyve, although with a different binding mode. The most potent PIKfyve inhibitors showed promising activity against SARS-CoV-2, with EC_50_ values less than 1 μM and lacking cytotoxicity.

## 4 Experimental section

### 4.1 Chemistry

For all reactions, analytical-grade solvents were used. Argon was used to carry out reactions under an inert atmosphere. Melting points were recorded using a Stuart SMP20 melting point apparatus. ^1^H, ^13^C, and ^19^F NMR spectra were recorded on a Bruker Avance 300 MHz instrument (^1^H NMR, 300 MHz; ^13^C NMR, 75 MHz; ^19^F NMR, 282 MHz), 500 MHz instrument (^1^H NMR, 500 MHz; ^13^C NMR, 125 MHz), and 600 MHz instrument (^1^H NMR, 600 MHz; ^13^C NMR, 150 MHz), respectively, using tetramethylsilane as the internal standard for ^1^H NMR spectra and DMSO-d_6_ (39.5 ppm) or CDCl_3_ (77.2 ppm) for ^13^C NMR spectra. Abbreviations used are as follows: s, singlet; d, doublet; t, triplet; q, quartet; m, multiplet; b = broad. Coupling constants are expressed in Hz. High-resolution mass spectra were acquired on a quadrupole orthogonal acceleration time-of-flight mass spectrometer (Synapt G2 HDMS, Waters, Milford, MA). Samples were infused at 3 mL/min, and spectra were obtained in the positive or negative ionization mode with a resolution of 15,000 (FWHM) using leucine enkephalin as lock mass. Precoated aluminum sheets (Fluka silica gel/TLC-cards, 254 nm) were used for TLC. Column chromatography was performed on silica gel 0.060–0.200 mm, 60 (Acros Organics). The ratio and percentage of solvents in the mobile phase are indicated as (v/v) and (%), respectively. The elution procedure is indicated as the percentage at the starting point, percentage of the end point, and running time when a gradient is applied.

#### 4.1.1 5-(3,4-Dimethoxyphenyl)-3-(pyridin-3-ylethynyl)isothiazolo[4,3-*b*]pyridine (2)

A solution of 3-bromo-5-(3,4-dimethoxyphenyl)isothiazolo[4,3-*b*]pyridine **1** (118 mg, 0.34 mmol), triethylamine (3 equiv, 141 µL) in THF (7 mL) was degassed with a flow of argon for 5 min. Then, Pd(PPh_3_)_2_Cl_2_ (0.02 equiv, 0.0068 mmol, 4.7 mg) and CuI (0.01 equiv, 0.0034 mmol, 0.65 mg) were added, and the reaction mixture was allowed to reach 30 °C. Subsequently, a solution of 3-pyridinylacetylene (1 equiv, 0.34 mmol, 35 µL) in THF (7 mL) was added slowly over a period of 30 min. The reaction was degassed a second time, filled with argon, and stirred at 30 °C overnight. After disappearance of the starting material as monitored by TLC, the volatiles were evaporated *in vacuo*. The crude residue was purified by flash chromatography using a mixture of hexane and acetone (in a ratio of 9:1) as the mobile phase, affording the title compound as a yellow solid in 75% yield (0.255 mmol, 95 mg).

^1^H NMR (600 MHz, CDCl_3_) *δ*: 3.98 (s, 3H, OCH_3_), 4.05 (s, 3H, OCH_3_), 7.00 (d, *J* = 8.4 Hz, 1H, arom H), 7.37 (ddd, *J* = 7.9, 4.9, 0.8 Hz, 1H, arom H), 7.73 (dd, *J* = 8.4, 2.1 Hz, 1H, arom H), 7.91–7.94 (m, 2H, arom H), 7.94–7.97 (m, 1H, arom H), 8.16 (d, *J* = 9.3 Hz, 1H, arom H), 8.64 (dd, *J* = 4.9, 1.6 Hz, 1H, arom H), 8.92 (d, *J* = 1.4 Hz, 1H, arom H).

^13^C NMR (150 MHz, CDCl_3_) *δ:* 56.0 (OCH_3_), 56.0 (OCH_3_), 80.9 (C), 103.6 (C), 111.0 (CH), 110.4 (CH), 119.6 (C), 121.0 (CH), 121.7 (CH), 123.2 (CH), 130.1 (CH), 131.1 (C), 138.5 (CH), 141.5 (C), 149.1 (C), 149.5 (C), 149.6 (CH), 151.3 (C), 152.2 (CH), 154.4 (C), 157.8 (C).

HRMS m/z [M+H]^+^ calculated for C_21_H_16_N_3_O_2_S_1_: 374.0958; found: 374.0945.

#### 4.1.2 6-Methoxy-3-nitropicolinonitrile (4)

CuCN (7.12 g, 79.6 mmol, 3 equiv) was added to a solution of 2-chloro-6-methoxy-3-nitropyridine **3** (5.00 g, 26.5 mmol) in DMF (50 mL) and stirred for 72 h at 100 °C. After cooling, the solution was poured into an EtOAc/water (1:1) mixture, stirred for 5 min, and then filtered over Celite. The layers were separated, and the aqueous layer was washed with EtOAc once. The combined organic phases were washed with brine, dried over Na_2_SO_4_, and evaporated *in vacuo*. The crude mixture was purified by silica gel flash chromatography using a mixture of PE/EtOAc (8:2) as the mobile phase, affording the title compound as a yellow solid in 60% yield (2.86 g, 16.0 mmol).

^1^H NMR (400 MHz, CDCl_3_) δ: 8.46 (d, *J* = 9.2 Hz, 1H), 7.10 (d, *J* = 9.2 Hz, 1H), 4.12 (s, 3H).

^13^C NMR (101 MHz, CDCl_3_) δ: 166.3, 135.5, 126.9, 115.9, 114.0, 55.8.

HRMS m/z [M+H]^+^ calculated for C_7_H_5_N_3_O_3_: 180.0404; found: 180.0413.

#### 4.1.3 3-Amino-6-methoxypicolinonitrile (5)

6-Methoxy-3-nitropicolinonitrile **4** (2.65 g, 14.8 mmol) and iron powder (2.48 g, 44.4 mmol, 3 equiv) were dissolved in acetic acid (75 mL) and stirred for 1 h at room temperature. After completion of the reaction, the mixture was evaporated to dryness. The residue was suspended in EtOAc, filtered over Celite, and the filter cake was washed with EtOAc thrice. The filtrate was washed with 1 M NaOH, followed by brine, and dried over Na_2_SO_4_. The solvent was removed *in vacuo*, and the compound was isolated as a brown solid in 97% yield (2.14 g, 14.3 mmol).

^1^H NMR (400 MHz, CDCl_3_) δ: 7.09 (d, *J* = 9.0 Hz, 1H), 6.81 (d, *J* = 8.9 Hz, 1H), 4.11 (s, 2H), 3.86 (s, 3H).

^13^C NMR (101 MHz, CDCl_3_) δ: 157.0, 142.5, 128.0, 117.7, 116.4, 111.4, 53.8.

HRMS m/z [M+H]^+^ calculated for C_7_H_7_N_3_O: 150.0662; found: 150.0667.

#### 4.1.4 3-Amino-6-methoxypyridine-2-carbothioamide (6)

3-Amino-6-methoxypicolinonitrile **5** (2.14 g, 14.3 mmol) was dissolved in absolute EtOH (60 mL), and P_2_S_5_ (3.83 g, 17.2 mmol, 1.2 equiv) was added to the solution. The mixture was stirred overnight at 75 °C. After completion of the reaction, as monitored by TLC, the solvent was evaporated *in vacuo*, and the residue was dissolved in EtOAc and washed twice with 1 M NaOH, followed by brine. The organic layer was dried over Na_2_SO_4_, the solvent was removed *in vacuo*, and the crude mixture was purified by silica gel chromatography (PE/EtOAc, 4:1), affording the title compound as a yellow solid in 80% yield (2.11 g, 11.5 mmol).

^1^H NMR (400 MHz, CDCl_3_) δ: 9.17 (s, 1H), 7.19 (s, 1H), 7.09 (d, *J* = 8.9 Hz, 1H), 6.81 (d, *J* = 8.9 Hz, 1H), 6.72 (s, 2H), 3.86 (s, 3H).

^13^C NMR (101 MHz, CDCl_3_) δ: 194.0, 152.8, 142.9, 132.4, 124.1, 118.7, 53.1.

HRMS m/z [M+H]^+^ calculated for C_7_H_9_N_3_OS: 184.0539; found: 184.0542.

#### 4.1.5 5-Methoxyisothiazolo[4,3-*b*]pyridin-3-amine (7)

To a solution of *3-*amino*-*6-methoxypyridine-2-carbothioamide **6** (800 mg, 4.37 mmol) in MeOH (400 mL), 35% aqueous solution of H_2_O_2_ (0.76 mL, 2 equiv) was added dropwise. The reaction was stirred overnight at room temperature. After disappearance of the starting material, as monitored by TLC, the solvent was evaporated to dryness, affording the title compound as a dark red solid in 96% yield (756 mg, 4.17 mmol), which was used as such in the following reaction.

^1^H NMR (400 MHz, CDCl_3_) δ: 7.68 (d, *J* = 9.4 Hz, 1H), 6.83 (d, *J* = 9.4 Hz, 1H), 5.13 (s, 2H), 3.98 (s, 3H).

^13^C NMR (101 MHz, CDCl_3_) δ: 164.3, 159.7, 153.2, 131.6, 117.8, 53.5.

HRMS m/z [M+H]^+^ calculated for C_7_H_7_N_3_OS: 182.0383; found: 182.0383.

#### 4.1.6 3-Bromoisothiazolo[4,3-*b*]pyridin-5(4H)-one (8)

5-Methoxyisothiazolo[4,3-*b*]pyridin-3-amine **7** (692 mg, 3.82 mmol) was dissolved in 48% aqueous HBr (60 mL) and stirred for 10 min at room temperature. CuBr (1.10 g, 7.64 mmol, 2 equiv) was added, and the mixture was cooled to 0 °C. An aqueous solution (28 mL) of NaNO_2_ (790 mg, 11.5 mmol, 3 equiv) was added dropwise (0.5 mL min^*−*1^). The reaction mixture was stirred for 2 h at 0 °C and then overnight at room temperature. The mixture was cooled to 0 °C, neutralized with 2M NaOH, and extracted with EtOAc thrice. The combined organic layers were evaporated under reduced pressure, and the residue was dissolved in 48% aqueous HBr (30 mL) and stirred for 2 h at 100 °C. The mixture was cooled to 0 °C and neutralized with a 2M NaOH solution. EtOAc was added, and the mixture was filtered. The filtrate layers were separated, and the aqueous layer was extracted with EtOAc thrice. The combined organic layers were washed with brine and then dried over Na_2_SO_4_. The solvent was removed *in vacuo*, and the crude was purified by silica gel chromatography (PE/EtOAc, 65:35), affording the title compound as a light brown solid in a 79% yield (653 mg, 2.83 mmol).

^1^H NMR (400 MHz, DMSO-*d*_6_) δ: 11.81 (s, 1H), 7.82 (d, *J* = 9.9 Hz, 1H), 6.66 (d, *J* = 9.9 Hz, 1H).

^13^C NMR (101 MHz, DMSO-*d*_6_) δ: 162.1, 151.4, 135.9, 133.3, 126.7, 112.2.

HRMS m/z [M+H]^+^ calculated for C_6_H_3_BrN_2_OS: 230.9223; found: 230.9224.

#### 4.1.7 3-Bromo-5-chloroisothiazolo[4,3-*b*]pyridine (9)

In an oven-dried reaction tube, 3-bromoisothiazolo[4,3-*b*]pyridin-5(4H)-one **8** (330 mg, 1.43 mmol) was dissolved in DCE (20 mL), and POCl_3_ (0.67 mL, 7.14 mmol, 5 equiv) was added dropwise. The reaction mixture was stirred for 6 h at 80 °C. The mixture was then cooled to 0 °C and neutralized with a saturated aqueous NaHCO_3_ solution. The mixture was then extracted with EtOAc twice; the combined organic layers were washed with brine and dried over Na_2_SO_4_. The solvent was removed *in vacuo*, and the crude mixture was purified by silica gel chromatography (PE/EtOAc, 85:15), affording the title compound as an off-white solid in 66% yield (235 mg, 0.94 mmol).

^1^H NMR (400 MHz, CDCl_3_) δ: 8.05 (d, *J* = 9.2 Hz, 1H), 7.37 (d, *J* = 9.2 Hz, 1H).

^13^C NMR (101 MHz, CDCl_3_) δ: 153.6, 153.0, 145.8, 134.6, 132.1, 125.7.

HRMS m/z [M+H]^+^ calculated for C_6_H_2_BrClN_2_S: 248.8884; found: 248.8887.

#### 4.1.8 3-Amino-6-chloropicolinonitrile (11)

Iron powder (5.10 g, 90 mmol, 3 eq) was added to a suspension of 6-chloro-3-nitropicolinonitrile **10** (5.51 g, 30 mmol) in acetic acid (100 mL) at 0 °C. The resulting mixture was stirred and warmed to room temperature over 2 h. After disappearance of the starting material on TLC, the mixture was concentrated under reduced pressure to dryness. The residue was suspended in EtOAc (100 mL), filtered through a pad of Celite, and washed thrice with EtOAc (50 mL). The combined filtrate was washed with 1 M NaOH and brine, and dried over Na_2_SO_4_. After filtration, the solvent was removed *in vacuo* to yield the title compound (4.5 g) as a yellow solid. The crude product contained a minor side product (3-amino-6-chloropicolinamide) but was used in the following steps without further purification.

^1^H NMR (300 MHz, CDCl_3_) δ: 7.28 (d, *J* = 8.8 Hz, 1H), 7.11 (d, *J* = 8.8 Hz, 1H), 4.52 (br., 2H) ppm.

^13^C NMR (75 MHz, DMSO-d_6_) δ: 148.96, 136.84, 129.73, 127.88, 116.37, 112.72 ppm.

HRMS m/z [M+H]^+^ calculated for C_6_H_4_ClN_3_: 154.0167; found: 154.0157.

#### 4.1.9 3-Amino-6-chloropyridine-2-carbothioamide (12)

To a suspension of 3-amino-6-chloropicolinonitrile (crude product, 4.50 g) in ethanol (120 mL), P_2_S_5_ (10.0 g, 22.5 mmol, 0.75 eq) was added. The mixture was heated under reflux until the starting material disappeared on TLC (approximately 6 h). The solvent was removed under reduced pressure to dryness, and the residue was purified by silica gel chromatography (from 0% to 100% dichloromethane in heptane, followed by 0%–20% EtOAc in dichloromethane), yielding the title compound (4.62 g, 82% over 2 steps) as a yellow solid.

^1^H NMR (300 MHz, CDCl_3_) δ: 9.20 (br., 1H), 7.20 (br., 1H), 7.18 (d, *J* = 8.7 Hz, 1H), 7.07 (d, *J* = 8.7 Hz, 1H), 6.96 (br., 2H) ppm.

^13^C NMR (75 MHz, CDCl_3_) δ: 193.62, 145.82, 135.46, 130.37, 128.87, 128.38 ppm.

HRMS m/z [M+H]+ calculated for C_6_H_6_ClN_3_S: 188.0044; found: 188.0050.

#### 4.1.10 5-Chloro-isothiazolo[4,3-*b*]pyridin-3-amine (13)

A 30% aqueous solution of H_2_O_2_ (1.70 mL, 16.6 mmol, 2 eq) was added to a solution of 3-amino-6-chloropyridine-2-carbothioamide (1.55 g, 8.3 mmol) in methanol (30 mL) at 0 °C. The resulting mixture was stirred and warmed to room temperature over 2 h. After the disappearance of the starting material on TLC, the solvents were removed under reduced pressure to yield the title compound (1.53 g) as a yellow solid. The product was used in the following steps without further purification.

^1^H NMR (300 MHz, CD_3_OD) δ: 7.71 (d, *J* = 9.2 Hz, 1H), 7.23 (d, *J* = 9.2 Hz, 1H) ppm.

^13^C NMR (75 MHz, DMSO-d6) δ: 172.03, 152.73, 142.95, 133.62, 131.87, 125.10 ppm.

HRMS m/z [M+H]+ calculated for C_6_H_4_ClN_3_S: 185.9887; found: 185.9888.

#### 4.1.11 3-Bromo-5-chloroisothiazolo[4,3-*b*]pyridine (9)

To a mixture of 5-chloroisothiazolo[4,3-*b*]pyridin-3-amine (1.53 g, 8.3 mmol), CuBr (2.38 g, 16.6 mmol, 2 eq) in 80 mL water, and 20 mL 48% HBr at −5 °C, a solution of NaNO_2_ (1.15 g, 16.6 mmol, 2 eq) in water (20 mL) was added dropwise (0.5 mL min^*−*1^). The resulting mixture was stirred at −5 °C for 2 h and warmed to room temperature over 3 h. The mixture was cooled to 0 °C and neutralized with a 2M NaOH solution. Then, EtOAc (100 mL) was added, and the suspension was filtrated through a pad of Celite, followed by washing with EtOAc (twice 50 mL). The organic phase was separated and washed with brine, followed by drying over Na_2_SO_4_. After filtration, the solvent was removed *in vacuo*. The crude product was purified by silica gel chromatography (0%–10% EtOAc in dichloromethane), yielding the title compound (1.62 g, 79% in 2 steps) as a yellow solid.

^1^H NMR (300 MHz, CDCl_3_) δ: 8.04 (d, *J* = 9.2 Hz, 1H), 7.36 (d, *J* = 9.2 Hz, 1H) ppm.

^13^C NMR (75 MHz, CDCl_3_) δ: 153.56, 153.02, 145.79, 134.61, 132.08, 125.68 ppm.

HRMS m/z [M+H]+ calculated for C_6_H_2_BrClN_2_S: 248.8884; found: 248.8882.

#### 4.1.12 5-Chloro-3-(pyridin-3-ylethynyl)isothiazolo[4,3-*b*]pyridine (14)

A solution of 3-bromo-5-chloroisothiazolo[4,3-*b*]pyridine **9** (1 equiv) and triethylamine (3 equiv) in THF was degassed with a flow of argon for 5 min. Then, Pd(PPh_3_)_2_Cl_2_ (0.02 equiv) and CuI (0.01 equiv) were added, and the reaction mixture was allowed to reach 30 °C. Subsequently, a solution of 3-ethynylpyridine (0.95 equiv) in THF was added slowly over a period of 30 min. The reaction was degassed a second time, filled with argon, and stirred at 30 °C overnight. After disappearance of the starting material, as monitored by TLC, the volatiles were evaporated *in vacuo*. The crude mixture was purified by column chromatography (PE/EtOAc, 3:1) and isolated as a yellow solid in 62% yield (141 mg, 0.52 mmol).

^1^H NMR (600 MHz, CDCl_3_) δ: 8.90 (dd, *J* = 2.2, 0.9 Hz, 1H), 8.65 (dd, *J* = 4.9, 1.7 Hz, 1H), 8.09 (d, *J* = 9.2 Hz, 1H), 7.96 (dt, *J* = 7.9, 1.9 Hz, 1H), 7.40 (d, *J* = 9.2 Hz, 1H), 7.37 (ddd, *J* = 7.9, 4.9, 0.9 Hz, 1H).

^13^C NMR (151 MHz, CDCl_3_) δ: 153.6, 152.8, 152.3, 149.9, 147.7, 142.8, 138.8, 132.1, 125.5, 123.2, 119.1, 104.5, 79.5.

HRMS m/z [M+H]^+^ calculated for C_13_H_6_ClN_3_S: 272.0044; found: 272.0041.

#### 4.1.13 5-((3,4-Dimethoxyphenyl)ethynyl)-3-(pyridin-3-ylethynyl)isothiazolo[4,3-*b*]pyridine (16a)

A solution of 5-chloro-3-(pyridin-3-ylethynyl)isothiazolo[4,3-*b*]pyridine **14** (1 equiv) and triethylamine (3 equiv) in THF was degassed with a flow of argon for 5 min. Then, Pd(PPh_3_)_2_Cl_2_ (0.02 equiv) and CuI (0.01 equiv) were added, and the reaction mixture was allowed to reach 30 °C. Subsequently, a solution of 4-ethynyl-1,2-dimethoxybenzene (0.95 equiv) in THF was added slowly over a period of 30 min. The reaction was degassed a second time, filled with argon, and stirred at 30 °C overnight. After disappearance of the starting material, as monitored by TLC, the volatiles were evaporated *in vacuo*. The crude mixture was purified by column chromatography (PE/EtOAc, 1:1) and isolated as an orange or yellow solid in 91% yield (60 mg, 0.15 mmol).

^1^H NMR (400 MHz, CDCl_3_) δ: 8.92 (d, *J* = 2.1 Hz, 1H), 8.64 (dd, *J* = 4.9, 1.6 Hz, 1H), 8.11 (d, *J* = 9.1 Hz, 1H), 7.98 (d, *J* = 7.9 Hz, 1H), 7.58 (d, *J* = 9.1 Hz, 1H), 7.37 (ddd, *J* = 7.9, 4.9, 0.9 Hz, 1H), 7.30 (dd, *J* = 8.3, 1.9 Hz, 1H), 7.18 (d, *J* = 1.9 Hz, 1H), 6.89 (d, *J* = 8.3 Hz, 1H), 3.93 (s, 3H), 3.93 (s, 3H).

^13^C NMR (101 MHz, CDCl_3_) δ: 153.6, 152.4, 150.7, 149.8, 149.0, 148.8, 145.3, 142.6, 138.8, 129.7, 127.2, 126.2, 123.2, 119.4, 114.9, 113.7, 111.1, 104.7, 93.7, 88.3, 80.3, 56.0, 56.0.

HRMS m/z [M+H]^+^ calculated for C_23_H_15_N_3_O_2_S: 398.0958; found: 398.0952.

#### 4.1.14 *N*-(3,4-dimethoxyphenyl)-3-(pyridin-3-ylethynyl)isothiazolo[4,3-*b*]pyridin-5-amine (16b)

3,4-Dimethoxybenzylamine (74 mg, 0.44 mmol, 3 equiv) was added to a solution of 5-chloro-3-(pyridin-3-ylethynyl)isothiazolo[4,3-*b*]pyridine **14** (40 mg, 0.15 mmol) in absolute EtOH (1.6 mL), and the reaction was stirred overnight at 75 °C. After disappearance of the starting material, as monitored by TLC, the solvent was evaporated *in vacuo*. The crude mixture was purified by silica gel column chromatography (PE/EtOAc, 3:7), affording the title compound as a brown solid in 70% yield (40 mg, 0.10 mmol).

^1^H NMR (600 MHz, CDCl_3_) δ: 8.85 (d, J = 1.5 Hz, 1H), 8.61 (dd, *J* = 4.9, 1.7 Hz, 1H), 7.89 (dt, *J* = 7.9, 1.9 Hz, 1H), 7.86 (d, *J* = 9.5 Hz, 1H), 7.65 (s, 1H), 7.34 (ddd, *J* = 7.9, 4.9, 0.9 Hz, 1H), 7.09 (s, 1H), 6.96 (dd, *J* = 8.5, 2.5 Hz, 1H), 6.92 (d, *J* = 9.5 Hz, 1H), 6.86 (d, *J* = 8.5 Hz, 1H), 3.89 (s, 3H), 3.82 (s, 3H).

^13^C NMR (151 MHz, CDCl_3_) δ: 155.0, 153.3, 152.1, 149.3, 148.3, 146.0, 138.4, 133.5, 132.7, 131.1, 123.2, 119.8, 117.4, 113.4, 111.6, 106.5, 100.8, 81.2, 56.2, 55.9.

HRMS m/z [M+H]^+^ calculated for C_21_H_16_N_4_O_2_S: 389.1067; found: 389.1057.

#### 4.1.15 N-(3,4-dimethoxybenzyl)-3-(pyridin-3-ylethynyl)isothiazolo[4,3-*b*]pyridin-5-amine (16c)

3,4-Dimethoxybenzylamine (74 mg, 0.44 mmol, 3 equiv) was added to a solution of 5-chloro-3-(pyridin-3-ylethynyl)isothiazolo[4,3-*b*]pyridine **22** (40 mg, 0.15 mmol) in absolute EtOH (1.6 mL), and the reaction was stirred overnight at 75 °C. After disappearance of the starting material, as monitored by TLC, the solvent was evaporated *in vacuo*. The crude residue was purified by silica gel column chromatography (PE/EtOAc, 3:7), affording the title compound as a brown solid in 98% yield (58 mg, 0.14 mmol).

^1^H NMR (600 MHz, CDCl_3_) δ: 8.86 (dd, *J* = 2.2, 0.9 Hz, 1H), 8.58 (dd, *J* = 4.9, 1.7 Hz, 1H), 7.89 (dt, *J* = 7.9, 1.9 Hz, 1H), 7.79 (d, *J* = 9.4 Hz, 1H), 7.32 (ddd, *J* = 7.9, 4.9, 0.9 Hz, 1H), 7.02 (d, *J* = 2.0 Hz, 1H), 6.98 (dd, *J* = 8.1, 2.0 Hz, 1H), 6.84 (d, *J* = 8.2 Hz, 1H), 6.70 (d, *J* = 9.4 Hz, 1H), 5.30 (s, 1H), 4.71 (d, *J* = 5.3 Hz, 2H), 3.87 (s, 3H), 3.85 (s, 3H).

^13^C NMR (151 MHz, CDCl_3_) δ: 156.6, 153.5, 152.1, 149.2, 149.1, 148.7, 148.6, 138.4, 132.3, 131.0, 130.8, 123.1, 120.5, 120.1, 111.6, 111.2, 100.6, 81.5, 56.0, 55.9, 45.9.

HRMS m/z [M+H]^+^ calculated for C_22_H_18_N_4_O_2_S: 403.1223; found: 403.1218.

#### 4.1.16 4-Ethynyl-1,2-dimethoxybenzene

4-Bromoveratrole **24** (600 mg, 2.76 mmol), triethylamine (1.16 mL, 8.29 mmol, 3 equiv), Pd(PPh_3_)_4_ (160 mg, 0.14 mmol, 0.05 equiv), and CuI (53 mg, 0.28 mmol, 0.1 equiv) were dissolved in dry THF (2.0 mL). Trimethylsilylacetylene (0.46 mL, 3.32 mmol, 1.2 equiv) was added to the mixture dropwise (0.03 mL min^*−*1^). The resulting mixture was stirred overnight at 50 °C. After cooling to room temperature, the reaction mixture was filtered through Celite and washed with EtOAc extensively. The solvent was removed in vacuo, and the residue was dissolved in dry THF (4.2 mL). A solution of 1M TBAF in THF (1.38 mL, 1.38 mmol, 0.5 equiv) was added to the mixture under argon at 0 °C, and the mixture was stirred at room temperature for 1 h. Following completion of the reaction, as monitored by TLC, the solvent was evaporated under reduced pressure, and the residue was dissolved in EtOAc, washed with water and then brine, and dried over Na_2_SO_4_. The solvent was evaporated *in vacuo*, and the crude mixture was purified by silica gel chromatography (PE/EtOAc, 9:1) and isolated as a light brown solid in 37% yield (165 mg, 1.01 mmol).

^1^H NMR (400 MHz, CDCl_3_) δ: 7.11 (dd, *J* = 8.3, 1.9 Hz, 1H), 6.99 (d, *J* = 1.8 Hz, 1H), 6.80 (d, *J* = 8.3 Hz, 1H), 3.89 (s, 3H), 3.88 (s, 3H), 3.00 (s, 1H).

#### 4.1.17 General procedures for structural modifications at positions 3 and 5 starting from 3-bromo-5-chloroisothiazolo[4,3-*b*]pyridine

##### 4.1.17.1 Procedure 1: Sonogashira coupling at position 3

A mixture of 3-bromo-5-chloroisothiazolo[4,3-*b*]pyridine **9** (1.0 eq), an appropriate acetylene (1.0 eq), and triethylamine (3.0 eq) in THF was degassed with argon. Then, Pd(PPh_3_)_2_Cl_2_ (0.02 eq) and CuI (0.01 eq) were added under the argon flow. The reaction mixture was stirred under an argon atmosphere at room temperature until the disappearance of the starting material, as monitored by TLC (4–12 h). The volatiles were evaporated *in vacuo*, and the crude residue was purified by silica gel flash chromatography to afford the desired compound in yields varying from 70% to 90%.

##### 4.1.17.2 Procedure 2: Suzuki coupling at position 3

A mixture of 3-bromo-5-chloroisothiazolo[4,3-*b*]pyridine **9** (1.0 eq), an appropriate boronic acid (1.0 eq), and K_2_CO_3_ (4.0 eq) in dioxane/water (ratio 4:1) was degassed with argon. Then, Pd(PPh_3_)_4_ (0.02 eq) was added under a flow of argon. The reaction mixture was stirred under an atmosphere of argon at room temperature until disappearance of the starting material, as monitored by TLC (4–12 h). The reaction mixture was diluted with EtOAc and washed with brine. The organic phase was evaporated *in vacuo*, and the crude residue was purified by silica gel flash chromatography to afford the desired compound in yields varying from 70% to 95%.

##### 4.1.17.3 Procedure 3: nucleophilic aromatic substitution at position 3

A mixture of 3-bromo-5-chloroisothiazolo[4,3-*b*]pyridine **9** (1.0 eq), an appropriate alkylamine (1.5 eq), and K_2_CO_3_ (2 eq) in dioxane (0.5 mmol/mL) was heated at 80 °C until the starting material almost disappeared on TLC. After concentration under reduced pressure, the residue was purified by silica gel flash chromatography to afford the title compounds in yields varying from 60% to 90%.

##### 4.1.17.4 Procedure 4: nucleophilic aromatic substitution of 3-bromo-5-chloroisothiazolo[4,3-b]pyridine

A mixture of 3-bromo-5-chloroisothiazolo[4,3-*b*]pyridine **9** (1.0 eq), an appropriate benzylamine (1.5 eq), and K_2_CO_3_ (2 eq) in dioxane (0.5 mmol/mL) was heated under reflux until the starting material almost disappeared on TLC. After concentration under reduced pressure, the residue was purified by silica gel flash chromatography to yield the title compounds (60%–90%).

##### 4.1.17.5 Procedure 5: nucleophilic aromatic substitution

To a mixture of 3-bromo-5-chloroisothiazolo[4,3-*b*]pyridine **9** (1.0 eq) and an appropriate aniline (1.5 eq) in dioxane (0.5 mmol/mL), a drop of concentrated hydrochloride acid was added. The resulting mixture was heated under reflux until all the starting material was consumed, according to TLC. After concentration under reduced pressure, the residue was purified by silica gel flash chromatography to afford the desired compound in yields ranging from 60% to 90%.

#### 4.1.18 3-Bromo-N-(pyridin-3-ylmethyl)isothiazolo[4,3-*b*]pyridin-5-amine (15a) (LJ 17184)

The title compound was synthesized from 3-bromo-5-chloroisothiazolo[4,3-*b*]pyridine **9** and pyridin-3-ylmethanamine using general procedure 4 in 62% yield.

^1^H NMR (300 MHz, CDCl_3_) δ: 8.72 (s, 1H), 8.53 (d, *J* = 4.5 Hz, 1H), 7.85 (d, *J* = 7.7 Hz, 1H), 7.73 (d, *J* = 9.4 Hz, 1H), 7.25 (m, 1H), 6.68 (d, *J* = 9.4 Hz, 1H), 5.38 (br., 1H), 4.77 (d, *J* = 5.7 Hz, 2H) ppm.

^13^C NMR (75 MHz, CDCl_3_) δ: 156.21, 153.27, 149.91, 148.83, 145.20, 136.31, 134.43, 130.75, 124.91, 123.50, 118.02, 43.22 ppm.

HRMS m/z [M+H]^+^ calculated for C_12_H_9_BrN_4_S: 320.9805; found: 320.9790.

#### 4.1.19 3-Bromo-N-(pyridin-2-ylmethyl)isothiazolo[4,3-*b*]pyridin-5-amine (15b) (LJ 19005)

The title compound was synthesized from 3-bromo-5-chloroisothiazolo[4,3-b]pyridine and pyridin-2-ylmethanamine using typical nucleophilic displacement procedure 4 in 62% yield.

^1^H NMR (300 MHz, CDCl_3_) δ: 8.58 (d, *J* = 4.6 Hz, 1H), 7.71 (d, *J* = 9.4 Hz, 1H), 7.68 (d, *J* = 7.6 Hz, 1H), 7.44 (d, *J* = 7.6 Hz, 1H), 7.22 (t, *J* = 7.5 Hz, 1H), 6.80 (d, *J* = 9.4 Hz, 1H), 6.41 (br. s, 1H), 4.86 (d, *J* = 4.5 Hz, 2H) ppm.

^13^C NMR (75 MHz, CDCl_3_) δ: 156.73, 156.46, 153.42, 148.89, 145.38, 136.70, 130.38, 123.99, 122.70, 122.38, 118.73, 46.26 ppm.

HRMS m/z [M+H]^+^ calculated for C_12_H_9_BrN_4_S: 320.9805; found: 320.9795.

#### 4.1.20 3-Bromo-N-(pyridin-4-ylmethyl)isothiazolo[4,3-*b*]pyridin-5-amine (15c) (LJ 19003)

The title compound was synthesized from 3-bromo-5-chloroisothiazolo[4,3-b]pyridine **9** and pyridin-4-ylmethanamine according to general procedure 4 in 50% yield.

^1^H NMR (300 MHz, CDCl_3_) δ: 8.56 (d, *J* = 5.0 Hz, 2H), 7.75 (d, *J* = 9.4 Hz, 1H), 7.36 (d, *J* = 5.0 Hz, 2H), 6.72 (d, *J* = 9.4 Hz, 1H), 5.47 (br. s, 1H), 4.98 (d, *J* = 5.8 Hz, 2H) ppm.

^13^C NMR (75 MHz, CDCl_3_+CD_3_OD) δ: 156.75, 153.35, 149.53, 148.94, 145.08, 130.33, 124.36, 123.31, 118.55, 44.09 ppm.

HRMS m/z [M+H]^+^ calculated for C_12_H_9_BrN_4_S: 320.9805; found: 320.9790.

#### 4.1.21 3-Bromo-N-(4-(4-methylpiperazin-1-yl)phenyl)isothiazolo[4,3-*b*]pyridin-5-amine (15 days)

The title compound was synthesized from 3-bromo-5-chloroisothiazolo[4,3-*b*]pyridine **9** and 4-(4-methylpiperazin-1-yl)aniline using general procedure 5 in 90% yield.

^1^H NMR (300 MHz, CDCl_3_) δ: 7.76 (d, *J* = 9.5 Hz, 1H), 7.48 (d, *J* = 8.6 Hz, 2H), 6.96 (d, *J* = 8.6 Hz, 2H), 6.94 (br., 1H), 6.88 (d, *J* = 9.5 Hz, 1H), 3.22 (m, 4H), 2.60 (m, 4H), 2.36 (s, 3H) ppm.

^13^C NMR (75 MHz, CDCl_3_) δ: 155.46, 153.26, 148.52, 145.32, 131.31, 130.87, 124.91, 123.29, 117.27, 116.87, 55.09, 49.39, 46.14 ppm.

HRMS m/z [M+H]^+^ calculated for C_17_H_18_BrN_5_S: 403.0467; found: 404.0540.

#### 4.1.22 3-Bromo-N-(2-fluorophenyl)isothiazolo[4,3-*b*]pyridin-5-amine (15e)

The title compound was synthesized from 3-bromo-5-chloroisothiazolo[4,3-*b*]pyridine **9** and 2-fluoroaniline using general procedure 5 in 85% yield.

^1^H NMR (300 MHz, CDCl_3_) δ: 8.90 (t, *J* = 8.2 Hz, 1H), 7.85 (d, *J* = 9.4 Hz, 1H), 7.23 (t, *J* = 7.8 Hz, 1H), 7.10 (m, 3H), 6.90 (d, *J* = 9.4 Hz, 1H) ppm.

^13^C NMR (75 MHz, CDCl_3_) δ: 153.64, 152.98, 152.76 (d, *J*_C–F_ = 241.2 Hz), 145.01, 131.14, 128.09 (d, *J*_C–F_ = 9.4 Hz), 124.69 (d, *J*_C-F_ = 3.6 Hz), 123.08 (d, *J*_C–F_ = 7.6 Hz), 121.11, 118.60, 114.71 (d, J_C–F_ = 19.1 Hz) ppm.

HRMS m/z [M+H]^+^ calculated for C_12_H_7_BrFN_3_S: 323.9601; found: 323.9605.

#### 4.1.23 3-Bromo-N-(2-fluorobenzyl)isothiazolo[4,3-*b*]pyridin-5-amine (15f)

The title compound was synthesized from 3-bromo-5-chloroisothiazolo[4,3-*b*]pyridine **9** and (2-fluorophenyl)methanamine using general procedure 4 in 70% yield.

^1^H NMR (300 MHz, CDCl_3_) δ: 7.70 (d, *J* = 9.4 Hz, 1H), 7.62 (t, *J* = 7.5 Hz, 1H), 7.25 (m, 1H), 7.10 (m, 2H), 6.66 (d, *J* = 9.4 Hz, 1H), 5.29 (br. s, 1H), 4.79 (d, *J* = 5.6 Hz, 2H) ppm.

^13^C NMR (75 MHz, CDCl_3_) δ: 161.36 (d, *J*_C–F_ = 244.5 Hz), 156.33, 153.30, 145.27, 131.33 (d, *J*_C–F_ = 4.2 Hz), 130.64, 129.34 (d, *J*_C–F_ = 8.1 Hz), 125.55 (d, *J*_C–F_ = 14.5 Hz), 124.57, 124.14 (d, J_C–F_ = 3.4 Hz), 118.00, 115.36 (*J*_C–F_ = 21.2 Hz), 39.76 ppm.

HRMS m/z [M+H]^+^ calculated for C_13_H_9_BrFN_3_S: 337.9758; found: 337.9760.

#### 4.1.24 3-Bromo-N-(3,4-difluorobenzyl)isothiazolo[4,3-*b*]pyridin-5-amine (15 g)

The title compound was synthesized from 3-bromo-5-chloroisothiazolo[4,3-*b*]pyridine **9** and (3,4-difluorophenyl)methanamine using general procedure 4 in 48% yield.

^1^H NMR (300 MHz, CDCl_3_) δ: 7.73 (d, J = 9.4 Hz, 1H), 7.28 (m, 1H), 7.16 (m, 2H), 6.66 (d, J = 9.4 Hz, 1H), 5.24 (br., 1H), 4.70 (d, J = 5.6 Hz, 2H) ppm.

^13^C NMR (75 MHz, DMSO-d6) δ: 157.31, 153.86, 149.63 (dd, J_C–F_ = 243.7 and 12.5 Hz), 148.91 (dd, J_C–F_ = 242.6 and 12.5 Hz), 145.38, 137.72 (dd, J_C–F_ = 5.9 and 3.3 Hz), 130.32, 125.16, 122.69, 120.15, 117.66 (d, J_C–F_ = 16.8 Hz), 117.51 (d, J_C–F_ = 16.9 Hz), 43.70 ppm.

HRMS m/z [M+H]^+^ calculated for C_13_H_8_BrF_2_N_3_S: 355.9664; found: 355.9659.

#### 4.1.25 3-Bromo-N-(3,4-difluorophenyl)isothiazolo[4,3-*b*]pyridin-5-amine (15 h)

The title compound was synthesized from 3-bromo-5-chloroisothiazolo[4,3-*b*]pyridine **9** and 3,4-difluoroaniline using general procedure 5 in 92% yield.

^1^H NMR (300 MHz, CDCl_3_) δ: 8.15 (m, 1H), 7.86 (d, *J* = 9.4 Hz, 1H), 7.25 (m, 1H), 7.15 (m, 1H), 6.85 (s, 1H), 6.84 (d, *J* = 9.4 Hz, 1H) ppm.

^13^C NMR (75 MHz, CDCl_3_+CD_3_OD) δ: 154.40, 153.19, 150.06 (dd, *J*_C–F_ = 242.9 and 12.9 Hz), 145.77 (dd, *J*_C–F_ = 241.2 and 12.9 Hz), 144.94, 137.22 (d, *J*_C–F_ = 9.3 Hz), 130.38, 126.24, 119.75, 116.95 (d, *J*_C–F_ = 17.9 Hz), 114.71, 108.95 (d, *J*_C–F_ = 22.6 Hz) ppm.

HRMS m/z [M+H]^+^ calculated for C_12_H_6_BrF_2_N_3_S: 341.9507; found: 341.9497.

#### 4.1.26 3-(Pyridin-3-ylethynyl)-5-(pyrrolidin-1-yl)isothiazolo[4,3-*b*]pyridine (16 days)

The title compound was synthesized from 5-chloro-3-(pyridin-3-ylethynyl)isothiazolo[4,3-*b*]pyridine and pyrrolidine using general procedure 3 in 81% yield.

^1^H NMR (300 MHz, CDCl_3_) δ: 8.85 (s, 1H), 8.57 (d, *J* = 4.2 Hz, 1H), 7.89 (d, *J* = 7.8 Hz, 1H), 7.80 (d, *J* = 9.5 Hz, 1H), 7.32 (dd, *J* = 4.2 Hz, 7.8 Hz, 1H), 6.89 (d, *J* = 9.5 Hz, 1H), 3.69 (br. s, 4H), 2.06 (br. s, 4H) ppm.

^13^C NMR (75 MHz, CDCl_3_) δ: 155.56, 153.04, 152.17, 149.53, 148.91, 138.28, 130.78, 130.33, 123.06, 120.32, 115.69, 100.42, 81.98, 47.31, 25.50 ppm.

HRMS m/z [M+H]^+^ calculated for C_17_H_14_N_4_S: 307.1012; found: 307.1014.

#### 4.1.27 5-(4-Methylpiperazin-1-yl)-3-(pyridin-3-ylethynyl)isothiazolo[4,3-*b*]pyridine (16e)

The title compound was synthesized from 5-chloro-3-(pyridin-3-ylethynyl)isothiazolo[4,3-*b*]pyridine and 1-methylpiperazine using general procedure 3 in 81% yield.

^1^H NMR (300 MHz, CDCl_3_) δ: 8.85 (d, *J* = 1.2 Hz, 1H), 8.59 (dd, *J* = 4.8 Hz, 1.2 Hz, 1H), 7.89 (d, *J* = 7.8 Hz, 1H), 7.85 (d, *J* = 9.8 Hz, 1H), 7.33 (dd, *J* = 7.8 Hz, 4.9 Hz, 1H), 7.12 (d, *J* = 9.4 Hz, 1H), 3.85 (t, *J* = 5.0 Hz, 4H), 2.58 (t, *J* = 5.0 Hz, 4H), 2.37 (s, 3H) ppm.

^13^C NMR (75 MHz, CDCl_3_) δ: 157.01, 152.89, 152.17, 149.08, 148.72, 138.34, 132.51, 130.79, 123.11, 120.11, 114.15, 100.79, 81.58, 54.89, 46.11, 45.19 ppm.

HRMS m/z [M+H]^+^ calculated for C_18_H_17_N_5_S: 336.1277; found: 336.1279.

#### 4.1.28 4-(3-((Pyridin-3-ylethynyl)isothiazolo[4,3-*b*]pyridin-5-yl)morpholine (16f)

The title compound was synthesized from 5-chloro-3-(pyridin-3-ylethynyl)isothiazolo[4,3-*b*]pyridine and morpholine using general procedure 3 in 67% yield.

^1^H NMR (300 MHz, CDCl_3_) δ: 8.85 (d, *J* = 1.2 Hz, 1H), 8.59 (dd, *J* = 4.8 Hz, 1.2 Hz, 1H), 7.88 (d, *J* = 7.8 Hz, 1H), 7.86 (d, *J* = 9.8 Hz, 1H), 7.33 (dd, *J* = 7.8 Hz, 4.9 Hz, 1H), 7.08 (d, *J* = 9.4 Hz, 1H), 3.85 (m, 4H), 3.83 (m, 4H), 2.37 (s, 3H) ppm.

^13^C NMR (75 MHz, CDCl_3_) δ: 157.14, 152.93, 152.17, 149.16, 148.56, 138.33, 133.10, 130.95, 123.11, 120.01, 113.82, 100.97, 81.43, 66.73, 45.61 ppm.

HRMS m/z [M+H]^+^ calculated for C_17_H_14_N_4_OS: 323.0961; found: 323.0969.

#### 4.1.29 *N*1,*N*1-Dimethyl-*N*2-(3-(pyridin-3-ylethynyl)isothiazolo[4,3-*b*]pyridin-5-yl)ethane-1,2-diamine (16 g)

The title compound was synthesized from 5-chloro-3-(pyridin-3-ylethynyl)isothiazolo[4,3-*b*]pyridine and N1,N1-dimethylethane-1,2-diamine using general procedure 3 in 30% yield.

^1^H NMR (300 MHz, CDCl_3_) δ: 8.85 (s, 1H), 8.58 (*d*, *J* = 4.2 Hz, 1H), 7.89 (d, *J* = 7.8 Hz, 1H), 7.73 (d, *J* = 9.5 Hz, 1H), 7.32 (dd, *J* = 4.2 Hz, 7.8 Hz, 1H), 6.73 (d, *J* = 9.5 Hz, 1H), 5.86 (br. s, 1H), 3.66 (m, 2H), 2.61 (t, *J* = 5.8 Hz, 2H), 2.30 (s, 6H) ppm.

^13^C NMR (75 MHz, CDCl_3_) δ: 156.88, 153.53, 152.12, 148.97, 138.37, 131.55, 130.33, 123.09, 120.17, 118.36, 100.34, 81.66, 57.60, 45.07, 38.56 ppm.

HRMS m/z [M+H]^+^ calculated for C_17_H_17_N_5_S: 324.1277; found: 324.1273.

#### 4.1.30 N-(2-Fluorophenyl)-3-(pyridin-3-ylethynyl)isothiazolo[4,3-*b*]pyridin-5-amine (16 h)

The title compound was synthesized from 5-chloro-3-(pyridin-3-ylethynyl)isothiazolo[4,3-*b*]pyridine and 2-fluoroaniline using general procedure 5 in % yield.

^1^H NMR (300 MHz, CDCl_3_) δ: 8.94 (m, 2H), 8.64 (d, *J* = 4.1 Hz, 1H), 7.93 (m, 2H), 7.38 (m, 1H), 7.00–7.30 (m, 5H), 6.96 (d, *J* = 9.5 Hz, 1H) ppm.

^13^C NMR (75 MHz, CDCl_3_+CD_3_OD) δ: 154.48, 153.59, 153.37 (d, *J*_C–F_ = 242.6 Hz), 151.37, 149.27, 148.57, 139.43, 138.91, 133.91, 132.23, 130.59, 127.97 (d, *J*_C–F_ = 10.0 Hz), 125.61, 124.08 (*J*_C–F_ = 3.4 Hz), 123.65, 123.49 (d, *J*_C–F_ = 7.5 Hz), 122.05, 120.35, 118.84, 114.92 (d, *J*_C–F_ = 19.4 Hz), 100.73, 81.63 ppm.

^19^F NMR (282 MHz, CDCl_3_) δ: −131.36 ppm.

HRMS m/z [M+H]^+^ calculated for C_19_H_11_FN_4_S: 347.0765; found: 347.0761.

#### 4.1.31 N-(4-Fluorophenyl)-3-(pyridin-3-ylethynyl)isothiazolo[4,3-*b*]pyridin-5-amine (16i)

The title compound was synthesized from 5-chloro-3-(pyridin-3-ylethynyl)isothiazolo[4,3-*b*]pyridine and 4-fluoroaniline using general procedure 5 in 77% yield.

^1^H NMR (300 MHz, DMSO-d_6_) δ: 9.98 (s, 1H), 8.86 (br. s, 1H), 8.67 (br. s, 1H), 8.15 (m, 2H), 8.03 (m, 2H), 7.58 (m, 1H), 7.20 (m, 3H) ppm.

^13^C NMR (75 MHz, DMSO-d_6_) δ: 157.89 (d, *J*_C–F_ = 237.7 Hz), 154.76, 153.55, 151.83, 150.04, 148.90, 138.80, 137.40, 132.02, 130.81, 124.40, 120.98 (d, *J*_C–F_ = 7.5 Hz), 119.50, 115.63 (d, *J*_C–F_ = 21.9 Hz), 101.24, 82.02 ppm.

^19^F NMR (282 MHz, CDCl_3_+CD_3_OD) δ: −120.22 ppm.

HRMS m/z [M+H]^+^ calculated for C_19_H_11_FN_4_S: 347.0765; found: 347.0761.

#### 4.1.32 N-(2-Fluorobenzyl)-3-(pyridin-3-ylethynyl)isothiazolo[4,3-*b*]pyridin-5-amine (16j)

The title compound was synthesized from 5-chloro-3-(pyridin-3-ylethynyl)isothiazolo[4,3-*b*]pyridine and (2-fluorophenyl)methanamine using general procedure 4 in 69% yield.

^1^H NMR (300 MHz, CDCl_3_+CD_3_OD) δ: 8.81 (br. s, 1H), 8.54 (d, *J* = 4.7 Hz, 1H), 8.00 (d, *J* = 7.8 Hz, 1H), 7.72 (d, *J* = 9.5 Hz, 1H), 7.59 (t, *J* = 7.3 Hz, 1H), 7.43 (m, 1H), 7.24 (m, 1H), 7.09 (m, 2H), 6.89 (d, *J* = 9.5 Hz, 1H), 4.80 (s, 2H) ppm.

^13^C NMR (75 MHz, CDCl_3_+CD_3_OD) δ: 161.18 (d, *J*_C–F_ = 244.5 Hz), 157.20, 153.56, 151.19, 148.85, 148.28, 139.08, 130.65 (d, *J*_C–F_ = 3.9 Hz), 129.91, 129.96 (*J*_C–F_ = 8.1 Hz), 125.61 (d, *J*_C–F_ = 14.7 Hz), 123.87 (d, *J*_C–F_ = 3.5 Hz), 123.68, 120.59, 118.71,115.07 (d, *J*_C–F_ = 21.4 Hz), 99.75, 81.80, 38.95 ppm.

^19^F NMR (282 MHz, CDCl_3_) δ: −118.84 ppm.

HRMS m/z [M+H]^+^ calculated for C_20_H_13_FN_4_S: 361.0918; found: 368.0915.

#### 4.1.33 N-(3-Fluorobenzyl)-3-(pyridin-3-ylethynyl)isothiazolo[4,3-*b*]pyridin-5-amine (16k)

The title compound was synthesized from 5-chloro-3-(pyridin-3-ylethynyl)isothiazolo[4,3-*b*]pyridine and (3-fluorophenyl)methanamine using general procedure 4 in 42% yield.

^1^H NMR (300 MHz, CDCl_3_) δ: 8.84 (s, 1H), 8.58 (d, *J* = 4.5 Hz, 1H), 7.88 (d, *J* = 7.8 Hz, 1H), 7.80 (d, *J* = 9.5 Hz, 1H), 7.24 (m, 4H), 6.98 (t, *J* = 7.6 Hz, 1H), 6.70 (d, J = 9.5 Hz, 1H), 5.31 (br.s, 1H), 4.78 (d, *J* = 5.5 Hz, 2H) ppm.

^13^C NMR (75 MHz, CDCl_3_+CD_3_OD) δ: 162.89 (d, *J*_C–F_ = 244.1 Hz), 157.09, 153.53, 151.32, 148.74, 148.37, 141.61 (d, *J*_C–F_ = 6.9 Hz), 139.05, 130.17, 129.91 (d, *J*_C–F_ = 8.2 Hz), 123.59, 120.48, 118.49, 114.94 (d, *J*_C–F_ = 21.5 Hz), 113.98 (d, *J*_C–F_ = 21.0 Hz), 99.94, 81.69, 44.83 ppm.

^19^F NMR (282 MHz, CDCl_3_) δ: −112.67 ppm.

HRMS m/z [M+H]^+^ calculated for C_20_H_13_FN_4_S: 361.0918; found: 368.0915.

#### 4.1.34 3-(Pyridin-3-ylethynyl)-N-(pyridin-2-ylmethyl)isothiazolo[4,3-*b*]pyridin-5-amine (16L)

The title compound was synthesized from 3-bromo-N-(pyridin-2-ylmethyl)isothiazolo[4,3-*b*]pyridin-5-amine and 3-ethynylpyridine according to general procedure 1 in 64% yield.

^1^H NMR (300 MHz, CDCl_3_) δ: 8.88 (s, 1H), 8.59 (m, 2H), 7.91 (d, *J* = 7.7 Hz, 1H), 7.78 (d, *J* = 9.4 Hz, 1H), 7.68 (t, *J* = 7.5 Hz, 1H), 7.43 (d, *J* = 7.7 Hz, 1H), 7.34 (dd, *J* = 7.7 and 5.7 Hz, 1H), 7.22 (dd, *J* = 6.8 and 5.7 Hz, 1H), 6.84 (d, *J* = 9.4 Hz, 1H), 6.49 (br. s, 1H), 4.90 (d, *J* = 4.5 Hz, 2H) ppm.

^13^C NMR (75 MHz, CDCl_3_) δ: 156.62, 156.50, 153.58, 152.20, 149.08, 148.95, 138.39, 136.69, 132.14, 130.53, 123.12, 122.51, 122.40, 120.19, 118.32, 100.44, 81.63, 46.26 ppm.

HRMS m/z [M+H]^+^ calculated for C_19_H_13_N_5_S: 344.0964; found: 344.0963.

#### 4.1.35 3-(Pyridin-3-ylethynyl)-N-(pyridin-3-ylmethyl)isothiazolo[4,3-*b*]pyridin-5-amine (16m)

The title compound was synthesized from 3-bromo-N-(pyridin-3-ylmethyl)isothiazolo[4,3-*b*]pyridin-5-amine and 3-ethynylpyridine using general procedure 1 in 70% yield.

^1^H NMR (300 MHz, CDCl_3_+ CD_3_OD) δ: 8.78 (s, 1H), 8.69 (s, 1H), 8.54 (d, *J* = 4.3 Hz, 1H), 8.42 (d, *J* = 3.1 Hz, 1H), 8.00 (m, 2H), 7.74 (d, *J* = 9.4 Hz, 1H), 7.47 (dd, *J* = 5.3 and 4.9 Hz, 1H), 7.35 (dd, *J* = 5.3 and 4.9 Hz, 1H), 6.90 (d, *J* = 9.4 Hz, 1H), 4.78 (s, 2H), 4.68 (s, 2H) ppm.

^13^C NMR (75 MHz, CDCl_3_+ CD_3_OD) δ: 157.07, 153.58, 151.15, 148.86, 148.34, 147.49, 139.09, 137.06, 129.99, 123.75, 118.87, 99.68, 81.67, 42.46 ppm.

HRMS m/z [M+H]^+^ calculated for C_19_H_13_N_5_S: 344.0964; found: 344.0961.

#### 4.1.36 3-(Pyridin-3-ylethynyl)-N-(pyridin-4-ylmethyl)isothiazolo[4,3-*b*]pyridin-5-amine (16n)

The title compound was synthesized from 3-bromo-N-(pyridin-4-ylmethyl)isothiazolo[4,3-*b*]pyridin-5-amine and 3-ethynylpyridine with the typical Sonogashira coupling procedure 1 in 67% yield.

^1^H NMR (300 MHz, CDCl_3_) δ: 8.82 (s, 1H), 8.57 (m, 3H), 7.84 (m, 2H), 7.33 (m, 3H), 6.74 (d, *J* = 9.4 Hz, 1H), 5.51 (br. s, 1H), 4.82 (d, *J* = 5.7 Hz, 2H) ppm.

^13^C NMR (75 MHz, CDCl_3_) δ: 156.27, 153.41, 152.09, 150.03, 149.18, 148.57, 147.92, 138.34, 133.26, 131.08, 123.15, 122.79, 119.99, 117.27, 100.74, 81.30, 44.64 ppm.

HRMS m/z [M+H]^+^ calculated for C_19_H_13_N_5_S: 344.0964; found: 344.0963.

#### 4.1.37 5-(3,4-Dichlorophenyl)-3-(pyridin-3-ylethynyl)isothiazolo[4,3-*b*]pyridine (16o)

The title compound was synthesized from 5-chloro-3-(pyridin-3-ylethynyl)isothiazolo[4,3-*b*]pyridine and (3,4-dichlorophenyl)boronic acid using general procedure 2 in 73% yield.

^1^H NMR (300 MHz, CDCl_3_) δ: 8.93 (d, *J* = 1.0 Hz, 1H), 8.66 (dd, *J* = 4.8 Hz, 1.3 Hz, 1H), 8.34 (d, *J* = 2.0 Hz, 1H), 8.32 (d, *J* = 9.3 Hz, 1H), 8.04 (dd, *J* = 8.3 Hz and 2.0 Hz, 1H), 7.98 (dt, *J* = 7.9 Hz, 1.0 Hz, 1H), 7.88 (d, *J* = 9.3 Hz, 1H), 7.60 (d, *J* = 8.3 Hz, 1H), 7.39 (dd, *J* = 7.9 Hz and 4.8 Hz, 1H) ppm.

^13^C NMR (75 MHz, CDCl_3_) δ: 155.83, 154.22, 152.34, 149.83, 149.02, 143.30, 138.66, 138.17, 134.62, 133.39, 130.91, 130.76, 129.63, 126.79, 123.24, 121.26, 119.40, 104.52, 80.38 ppm.

HRMS m/z [M+H]^+^ calculated for C_19_H_9_Cl_2_N_3_S: 381.9967; found: 381.9958.

#### 4.1.38 5-(4-Fluorophenyl)-3-(pyridin-3-ylethynyl)isothiazolo[4,3-*b*]pyridine (16p)

The title compound was synthesized from 5-chloro-3-(pyridin-3-ylethynyl)isothiazolo[4,3-*b*]pyridine and (4-fluorophenyl)boronic acid using general procedure 2 in 93% yield.

^1^H NMR (300 MHz, CDCl_3_) δ: 8.92 (d, *J* = 1.4 Hz, 1H), 8.64 (dd, *J* = 4.8 Hz and 1.3 Hz, 1H), 8.19 (m, 2H), 7.95 (m, 2H), 7.48 (m, 1H), 7.37 (m, 2H), 7.23 (m, 1H) ppm.

^13^C NMR (75 MHz, CDCl_3_) δ: 161.03 (*J*_C–F_ = 174.2 Hz), 155.64, 154.12, 152.34, 149.70, 149.01, 143.06, 138.62, 131.84, 131.69, 129.69, 126.83 (*J*_C–F_ = 11.3 Hz), 125.26 (*J*_C–F_ = 9.2 Hz), 124.85, 124.82, 123.16, 119.48, 116.38 (*J*_C–F_ = 22.8 Hz), 104.18, 80.53 ppm.

^19^F NMR (282 MHz, CDCl_3_) δ: −115.73 ppm.

HRMS m/z [M+H]^+^ calculated for C_19_H_10_FN_3_S: 332.0652; found: 332.0651.

#### 4.1.39 3-(3,4-Dimethoxyphenyl)-*N*-(pyridin-3-ylmethyl)isothiazolo[4,3-*b*]pyridin-5-amine (17a)

The title compound was synthesized from 3-bromo-N-(pyridin-3-ylmethyl)isothiazolo[4,3-*b*]pyridin-5-amine and (3,4-dimethoxyphenyl)boronic acid using general procedure 2 in 92% yield.

^1^H NMR (300 MHz, CDCl_3_ + CD_3_OD) δ: 8.61 (s, 1H), 8.42 (d, *J* = 4.7 Hz, 1H), 7.95 (s, 1H), 7.86 (d, *J* = 7.7 Hz, 1H), 7.70 (d, *J* = 9.4 Hz, 1H), 7.57 (m, 1H), 7.36 (m, 1H), 6.93 (d, *J* = 7.7 Hz, 1H), 6.90 (d, *J* = 9.4 Hz, 1H), 4.65 (s, 2H), 3.93 (s, 3H), 3.75 (s, 3H) ppm.

^13^C NMR (75 MHz, CDCl_3_ + CD_3_OD) δ: 156.15, 155.09, 151.68, 149.50, 148.89, 148.51, 148.09, 147.35, 142.28, 136.09, 129.85, 124.35, 123.88, 120.49, 118.21, 111.44, 110.70, 55.60, 55.41, 42.30 ppm.

HRMS m/z [M+H]^+^ calculated for C_20_H_18_N_4_O_2_S: 379.1223; found: 379.1224.

#### 4.1.40 3-(3-Fluoro-4-methoxyphenyl)-N-(pyridin-3-ylmethyl)isothiazolo[4,3-*b*]pyridin-5-amine (17b)

The title compound was synthesized from 3-bromo-N-(pyridin-3-ylmethyl)isothiazolo[4,3-*b*]pyridin-5-amine and 3-fluoro-4-methoxyphenyl boronic acid using general procedure 2 in 89% yield.

^1^H NMR (300 MHz, CDCl_3_+CD_3_OD) δ: 8.69 (s, 1H), 8.53 (d, *J* = 4.2 Hz, 1H), 8.08 (d, *J* = 12.8 Hz, 1H), 7.88 (d, *J* = 7.6 Hz, 1H), 7.69 (m, 2H), 7.34 (m, 1H), 7.03 (t, *J* = 8.6 Hz, 1H), 6.86 (d, *J* = 9.4 Hz, 1H), 4.74 (s, 2H), 3.96 (s, 3H) ppm.

^13^C NMR (75 MHz, CDCl_3_+CD_3_OD) δ: 155.89, 154.99, 152.17 (d, *J*_C–F_ = 243.7 Hz), 150.22, 148.14, 147.85 (d, *J*_C–F_ = 10.9 Hz), 147.40, 142.53, 136.18, 135.86, 129.99, 124.47 (d, *J*_C–F_ = 7.8 Hz), 123.83, 123.52 (d, *J*_C–F_ = 3.5 Hz), 118.13, 115.05 (d, *J*_C–F_ = 20.5 Hz), 113.40 (d, *J*_C–F_ = 1.8 Hz), 55.12, 42.82 ppm.

^19^F NMR (282 MHz, CDCl_3_) δ: −134.45 ppm.

HRMS m/z [M+H]^+^ calculated for C_19_H_15_FN_4_OS: 367.1023; found: 367.1021.

#### 4.1.41 3-(4-Fluoro-3-methoxyphenyl)-N-(pyridin-3-ylmethyl)isothiazolo[4,3-*b*]pyridin-5-amine (17c)

The title compound was synthesized from 3-bromo-*N*-(pyridin-3-ylmethyl)isothiazolo[4,3-*b*]pyridin-5-amine and 4-fluoro-3-methoxyphenylboronic acid using general procedure 2 in 93% yield.

^1^H NMR (300 MHz, CDCl_3_) δ: 8.66 (s, 1H), 8.54 (s, 1H), 8.02 (d, J = 8.2 Hz, 1H), 7.80 (d, J = 9.4 Hz, 1H), 7.74 (m, 1H), 7.50 (br., 1H), 7.25 (m, 1H), 7.13 (t, J = 8.8 Hz, 1H), 6.73 (d, J = 9.4 Hz, 1H), 5.29 (br., 1H), 4.80 (d, J = 5.4 Hz, 2H), 3.78 (s, 3H) ppm.

^13^C NMR (75 MHz, CDCl_3_) δ: 155.59, 155.07, 152.49 (d, *J*_C–F_ = 247.9 Hz), 151.60, 149.15, 148.92, 147.80 (d, *J*_C–F_ = 10.9 Hz), 142.63, 135.22, 134.53, 131.11, 127.92 (d, *J*_C–F_ = 4.0 Hz), 123.58, 120.34 (d, *J*_C–F_ = 6.7 Hz), 117.12, 116.40 (d, *J*_C–F_ = 18.7 Hz), 112.72 (d, *J*_C–F_ = 1.0 Hz), 55.02, 49.28 ppm.

^19^F NMR (282 MHz, CDCl_3_) δ: −133.85 ppm.

HRMS m/z [M+H]^+^ calculated for C_19_H_15_FN_4_OS: 367.1023; found: 367.1020.

#### 4.1.42 3-(3,4-Difluorophenyl)-N-(pyridin-3-ylmethyl)isothiazolo[4,3-*b*]pyridin-5-amine (17 days)

The title compound was synthesized from 3-bromo-N-(pyridin-3-ylmethyl)isothiazolo[4,3-*b*]pyridin-5-amine and 3,4-difluorophenylboronic acid using general procedure 2 in 85% yield.

^1^H NMR (300 MHz, CDCl_3_) δ: 8.69 (s, 1H), 8.54 (d, *J* = 4.4 Hz, 1H), 8.18 (t, *J* = 9.1 Hz, 1H), 7.77 (m, 2H), 7.66 (d, *J* = 8.4 Hz, 1H), 7.24 (m, 2H), 6.75 (d, *J* = 9.4 Hz, 1H), 5.39 (br., 1H), 4.76 (d, *J* = 5.5 Hz, 2H) ppm.

^13^C NMR (75 MHz, CDCl_3_+CD_3_OD) δ: 156.14, 155.06, 150.31 (dd, *J*_C–F_ = 247.9 and 14.3 Hz), 150.20 (dd, *J*_C–F_ = 248.0 and 14.3 Hz), 148.67, 148.12, 147.52, 142.89, 136.00, 135.63, 130.13, 128.37 (t, *J*_C–F_ = 5.6 Hz), 123.79, 123.56 (t, *J*_C–F_ = 4.8 Hz), 118.19, 117.53 (dd, *J*_C–F_ = 12.3 and 6.2 Hz), 116.25 (t, *J*_C–F_ = 10.2 Hz), 42.87 ppm.

^19^F NMR (282 MHz, CDCl_3_) δ: −136.70 (d, J_F–F_ = 21 Hz, 1F), −136.79 (d, J_F–F_ = 21 Hz, 1F) ppm.

HRMS m/z [M+H]^+^ calculated for C_18_H_12_F_2_N_4_S: 355.0823; found: 355.0822.

#### 4.1.43 3-(4-Chloro-3-methoxyphenyl)-N-(pyridin-3-ylmethyl)isothiazolo[4,3-*b*]pyridin-5-amine (17e)

The title compound was synthesized from 3-bromo-N-(pyridin-3-ylmethyl)isothiazolo[4,3-*b*]pyridin-5-amine and 4-chloro-3-methoxyphenyl boronic acid using general procedure 2 in 87% yield.

^1^H NMR (300 MHz, CDCl_3_+CD_3_OD) δ: 8.59 (s, 1H), 8.44 (d, *J* = 4.4 Hz, 1H), 8.01 (s, 1H), 7.84 (d, *J* = 7.6 Hz, 1H), 7.74 (d, *J* = 9.4 Hz, 1H), 7.50 (d, *J* = 8.2 Hz, 1H), 7.37 (m, 2H), 6.88 (d, *J* = 9.4 Hz, 1H), 4.80 (s, 2H), 3.79 (s, 3H) ppm.

^13^C NMR (75 MHz, CDCl_3_+CD_3_OD) δ: 156.37, 155.19, 155.03, 150.12, 148.12, 147.59, 142.94, 136.00, 131.08, 130.25, 130.13, 123.87, 122.54, 120.32, 118.28, 110.99, 55.74, 42.43 ppm.

HRMS m/z [M+H]^+^ calculated for C_19_H_15_ClN_4_OS: 383.0728; found: 383.0728.

#### 4.1.44 3-(3-Chloro-4-methoxyphenyl)-N-(pyridin-3-ylmethyl)isothiazolo[4,3-*b*]pyridin-5-amine (17f)

The title compound was synthesized from 3-bromo-N-(pyridin-3-ylmethyl)isothiazolo[4,3-*b*]pyridin-5-amine and 3-chloro-4-methoxyphenyl boronic acid using general procedure 2 in 85% yield.

^1^H NMR (300 MHz, CDCl_3_+CD_3_OD) δ: 8.64 (s, 1H), 8.38 (m, 2H), 7.91 (d, *J* = 7.4 Hz, 1H), 7.82 (d, *J* = 8.4 Hz, 1H), 7.70 (d, *J* = 9.4 Hz, 1H), 7.35 (m, 1H), 7.02 (d, *J* = 8.4 Hz, 1H), 6.88 (d, *J* = 9.4 Hz, 1H), 4.76 (s, 2H), 3.96 (s, 3H) ppm.

^13^C NMR (75 MHz, CDCl_3_+CD_3_OD) δ: 156.04, 155.13, 155.01, 149.86, 148.12, 147.26, 142.54, 136.23, 129.86, 128.95, 124.84, 123.85, 122.68, 118.29, 112.16, 56.00, 42.59 ppm.

HRMS m/z [M+H]^+^ calculated for C_19_H_15_ClN_4_OS: 383.0728; found: 383.0730.

#### 4.1.45 3-(4-Methoxyphenyl)-N-(pyridin-3-ylmethyl)isothiazolo[4,3-*b*]pyridin-5-amine (17g)

The title compound was synthesized from 3-bromo-N-(pyridin-3-ylmethyl)isothiazolo[4,3-*b*]pyridin-5-amine and (4-methoxyphenyl)boronic acid using general procedure 2 in 86% yield.

^1^H NMR (300 MHz, CDCl_3_) δ: 8.70 (s, 1H), 8.53 (d, *J* = 4.4 Hz, 1H), 8.02 (d, *J* = 8.4 Hz, 2H), 7.76 (d, *J* = 9.2 Hz, 2H), 7.26 (m, 1H), 6.96 (d, J = 8.4 Hz, 2H), 6.70 (d, *J* = 9.2 Hz, 1H), 5.26 (br., 1H), 4.75 (d, *J* = 5.5 Hz, 2H), 3.86 (s, 3H) ppm.

^13^C NMR (75 MHz, CDCl_3_) δ: 159.99, 155.18, 154.91, 152.99, 149.31, 148.66, 142.32, 135.33, 134.94, 130.84, 128.96, 124.12, 123.49, 116.94, 114.28, 55.38, 43.40 ppm.

HRMS m/z [M+H]^+^ calculated for C_19_H_16_N_4_OS: 349.1123; found: 349.1115.

#### 4.1.46 3-(4-Fluorophenyl)-N-(pyridin-3-ylmethyl)isothiazolo[4,3-*b*]pyridin-5-amine (17h)

The title compound was synthesized from 3-bromo-N-(pyridin-3-ylmethyl)isothiazolo[4,3-*b*]pyridin-5-amine and (4-fluorophenyl)boronic acid using general procedure 2 in 71% yield.

^1^H NMR (300 MHz, CDCl_3_) δ: 8.70 (s, 1H), 8.55 (d, *J* = 4.3 Hz, 1H), 8.01 (dd, *J* = 8.2 Hz and 5.6 Hz, 2H), 7.77 (m, 2H), 7.28 (m, 1H), 7.12 (t, *J* = 8.5 Hz, 2H), 6.73 (d, *J* = 9.4 Hz, 1H), 5.30 (br., 1H), 4.75 (d, *J* = 5.5 Hz, 2H) ppm.

^13^C NMR (75 MHz, CDCl_3_) δ: 162.78 (d, *J*_C–F_ = 248.0 Hz), 155.47, 154.94, 151.59, 149.26, 148.73, 142.65, 135.22, 134.78, 130.92, 129.38 (d, *J*_C–F_ = 8.0 Hz), 127.58, 123.49, 117.10, 116.40 (d, *J*_C–F_ = 21.6 Hz), 43.46 ppm.

^19^F NMR (282 MHz, CDCl_3_) δ: −111.95 ppm.

HRMS m/z [M+H]^+^ calculated for C_18_H_13_FN_4_S: 337.0927; found: 337.0918.

#### 4.1.47 3-(Benzo[d][1,3]dioxol-5-yl)-N-(pyridin-3-ylmethyl)isothiazolo[4,3-*b*]pyridin-5-amine (17i)

The title compound was synthesized from 3-bromo-N-(pyridin-3-ylmethyl)isothiazolo[4,3-*b*]pyridin-5-amine and benzo[d][1,3]dioxol-5-ylboronic acid using general procedure 2 in 83% yield.

^1^H NMR (300 MHz, CDCl_3_) δ: 8.69 (s, 1H), 8.53 (d, *J* = 4.4 Hz, 1H), 7.75 (m, 3H), 7.52 (d, *J* = 8.1 Hz, 1H), 7.28 (m, 1H), 6.86 (d, *J* = 8.1 Hz, 1H), 6.70 (d, *J* = 9.4 Hz, 1H), 6.02 (s, 2H), 5.31 (br., 1H), 4.75 (d, *J* = 5.5 Hz, 2H) ppm.

^13^C NMR (75 MHz, CDCl_3_) δ: 155.16, 154.95, 152.68, 149.32, 148.76, 148.03, 147.97, 142.38, 135.44, 134.73, 130.95, 125.41, 123.53, 121.79, 116.98, 108.77, 107.97, 101.32, 43.45 ppm.

HRMS m/z [M+H]^+^ calculated for C_19_H_14_N_4_O_2_S: 363.0910; found: 363.0911.

#### 4.1.48 3-(3,4-Dimethoxyphenyl)-N-(2-fluorophenyl)isothiazolo[4,3-*b*]pyridin-5-amine (17j)

The title compound was synthesized from 3-bromo-N-(2-fluorophenyl)isothiazolo[4,3-*b*]pyridin-5-amine and (3,4-dimethoxyphenyl)boronic acid using general procedure 2 in 92% yield.

^1^H NMR (300 MHz, CDCl_3_) δ: 8.78 (t, *J* = 8.2 Hz, 1H), 7.89 (d, *J* = 9.4 Hz, 1H), 7.81 (s, 1H), 7.61 (d, *J* = 8.3 Hz, 1H), 7.14 (t, *J* = 8.1 Hz, 2H), 7.00 (m, 2H), 6.90 (m, 2H), 3.97 (s, 3H), 3.90 (s, 3H) ppm.

^13^C NMR (75 MHz, CDCl_3_) δ: 155.25, 154.67, 152.76 (d, *J*_C–F_ = 241.2 Hz), 152.58, 149.94, 149.34, 141.88, 131.18, 128.43 (d, *J*_C–F_ = 9.6 Hz), 124.30 (*J*_C–F_ = 3.5 Hz), 123.98, 122.62 (*J*_C–F_ = 7.5 Hz), 120.99, 120.79, 117.92, 114.80 (d, *J*_C–F_ = 19.2 Hz), 111.37, 111.03, 56.16, 55.99 ppm.

^19^F NMR (282 MHz, CDCl_3_) δ: −131.75 ppm.

HRMS m/z [M+H]^+^ calculated for C_20_H_16_FN_3_O_2_S: 382.1020; found: 382.1020.

#### 4.1.49 3-(3,4-Dimethoxyphenyl)-N-(2-fluorobenzyl)isothiazolo[4,3-*b*]pyridin-5-amine (17k)

The title compound was synthesized from 3-bromo-N-(2-fluorobenzyl)isothiazolo[4,3-*b*]pyridin-5-amine and (3,4-dimethoxyphenyl)boronic acid using general procedure 2 in 89% yield.

^1^H NMR (300 MHz, CDCl_3_) δ: 8.10 (s, 1H), 7.76 (d, *J* = 9.4 Hz, 1H), 7.60 (d, *J* = 8.3 Hz, 1H), 7.44 (t, *J* = 7.3 Hz, 1H), 7.26 (m, 1H), 7.08 (m, 2H), 6.93 (d, *J* = 8.3 Hz, 1H), 6.70 (d, *J* = 9.4 Hz, 1H), 5.10 (br., 1H), 4.85 (d, *J* = 5.6 Hz, 2H), 3.93 (s, 3H), 3.84 (s, 3H) ppm.

^13^C NMR (75 MHz, CDCl_3_) δ: 160.79 (d, *J*_C–F_ = 243.7 Hz), 156.17, 155.12, 151.56, 149.33, 148.89, 142.41, 129.85, 129.20 (d, *J*_C–F_ = 4.4 Hz), 128.62 (d, *J*_C–F_ = 8.0 Hz), 126.07 (*J*_C–F_ = 14.6 Hz), 124.51, 124.09 (d, *J*_C–F_ = 3.4 Hz), 120.31, 118.22, 114.99 (d, *J*_C–F_ = 21.2 Hz), 111.36, 110.62, 55.72, 55.44, 38.66 ppm.

^19^F NMR (282 MHz, CDCl_3_) δ: −119.01 ppm.

HRMS m/z [M+H]^+^ calculated for C_21_H_18_FN_3_O_2_S: 396.1176; found: 396.1179.

#### 4.1.50 *N*-(3,4-Difluorophenyl)-3-(3,4-dimethoxyphenyl)isothiazolo[4,3-*b*]pyridin-5-amine (17L)

The title compound was synthesized from 3-bromo-N-(3,4-difluorophenyl)isothiazolo[4,3-*b*]pyridin-5-amine and (3,4-dimethoxyphenyl)boronic acid using general procedure 2 in 88% yield.

^1^H NMR (300 MHz, CDCl_3_) δ: 8.11 (m, 1H), 7.88 (d, *J* = 9.4 Hz, 1H), 7.74 (d, *J* = 8.3 Hz, 1H), 7.63 (s, 1H), 7.18 (m, 1H), 7.10 (m, 1H), 7.02 (d, *J* = 8.3 Hz, 1H), 6.82 (d, *J* = 9.4 Hz, 1H), 6.80 (br., 1H), 3.97 (s, 3H), 3.92 (s, 1H) ppm.

^13^C NMR (75 MHz, DMSO-d6) δ: 154.93, 153.76, 153.68, 149.42 (dd, *J*_C–F_ = 242.4 & 12.9 Hz), 150.24, 149.63, 146.67 (dd, *J*_C–F_ = 238.3 & 12.7 Hz), 141.64, 138.23 (d, *J*_C–F_ = 7.3 Hz), 131.18, 123.63, 120.94, 119.65, 117.85 (d, *J*_C–F_ = 17.5 Hz), 115.18, 112.56, 111.27, 107.61 (d, *J*_C–F_ = 22.0 Hz), 56.07, 55.98 ppm.

^19^F NMR (282 MHz, CDCl_3_) δ: −135.68 (d, J_F–F_ = 22 Hz, 1F), −144.24 (d, J_F–F_ = 22 Hz, 1F) ppm.

HRMS m/z [M+H]^+^ calculated for C_20_H_15_F_2_N_3_O_2_S: 400.0931; found: 400.0914.

#### 4.1.51 *N*-(3,4-Difluorobenzyl)-3-(3,4-dimethoxyphenyl)isothiazolo[4,3-*b*]pyridin-5-amine (17m)

The title compound was synthesized from 3-bromo-N-(3,4-difluorobenzyl)isothiazolo[4,3-*b*]pyridin-5-amine and (3,4-dimethoxyphenyl)boronic acid using general procedure 2 in 73% yield.

^1^H NMR (300 MHz, CDCl_3_) δ: 7.95 (s, 1H), 7.78 (d, *J* = 9.4 Hz, 1H), 7.58 (d, *J* = 8.3 Hz, 1H), 7.23 (m, 1H), 7.12 (m, 2H), 6.92 (d, *J* = 8.3 Hz, 1H), 6.70 (d, *J* = 9.4 Hz, 1H), 5.14 (m, 1H), 4.73 (d, *J* = 5.6 Hz, 2H), 3.93 (s, 3H), 3.81 (s, 1H) ppm.

^13^C NMR (75 MHz, CDCl_3_) δ: 155.27, 155.04, 152.79, 150.37 (dd, *J*_C–F_ = 247.0 & 12.5 Hz), 149.57 (dd, *J*_C–F_ = 246.1 & 12.4 Hz), 149.54, 149.02, 142.38, 136.26 (t, *J*_C–F_ = 4.3 Hz), 131.01, 124.41, 123.33 (dd, *J*_C–F_ = 6.0 & 3.6 Hz), 120.50, 117.40 (d, *J*_C–F_ = 17.1 Hz), 116.98, 116.46 (d, *J*_C–F_ = 17.3 Hz), 111.33, 110.67, 67.09, 55.94, 55.68, 44.62 ppm.

^19^F NMR (282 MHz, CDCl_3_) δ: −137.19 (d, J_F–F_ = 21 Hz, 1F), −139.71 (d, J_F–F_ = 21 Hz, 1F) ppm.

HRMS m/z [M+H]^+^ calculated for C_21_H_17_F_2_N_3_O_2_S: 414.1088; found: 414.1078.

#### 4.1.52 3-(3,4-Dimethoxyphenyl)-*N*-(4-(4-methylpiperazin-1-yl)phenyl)isothiazolo[4,3-*b*]pyridin-5-amine (17n)

The title compound was synthesized from 3-bromo-N-(4-(4-methylpiperazin-1-yl)phenyl)isothiazolo[4,3-*b*]pyridin-5-amine and (3,4-dimethoxyphenyl)boronic acid using general procedure 2 in 76% yield.

^1^H NMR (300 MHz, CDCl_3_) δ: 7.92 (s, 1H), 7.80 (d, *J* = 9.4 Hz, 1H), 7.57 (m, 3H), 6.94 (m, 3H), 6.84 (d, *J* = 9.4 Hz, 1H), 6.67 (s, 1H), 3.95 (s, 3H), 3.87 (s, 3H), 3.20 (m, 4H), 2.61 (m, 4H), 2.37 (s, 3H) ppm.

^13^C NMR (75 MHz, CDCl_3_) δ: 154.91, 153.89, 153.56, 149.67, 149.24, 147.85, 142.35, 132.17, 130.89, 124.33, 122.36, 120.76, 117.24, 116.82, 111.36, 111.00, 56.16, 55.99, 55.09, 49.51, 46.08 ppm.

HRMS m/z [M+H]^+^ calculated for C_25_H_27_N_5_O_2_S: 462.1958; found: 462.1966.

#### 4.1.53 3,5-Bis(3,4-dimethoxyphenyl)isothiazolo[4,3-*b*]pyridine (17o)

The title compound was synthesized from 3-bromo-5-chloroisothiazolo[4,3-*b*]pyridine and 2.5 equivalents of (3,4-dimethoxyphenyl)boronic acid using general procedure 2 in 83% yield.

^1^H NMR (300 MHz, CDCl_3_) δ: 8.10 (m, 2H), 7.82 (m, 3H), 7.72 (d, *J* = 8.3 Hz, 1H), 7.00 (m, 2H), 4.03 (s, 3H), 4.00 (s, 3H), 3.98 (s, 6H) ppm.

^13^C NMR (75 MHz, CDCl_3_) δ: 160.76, 156.28, 155.98, 150.86, 150.45, 149.27, 144.09; 131.90, 129.84, 123.89, 121.46, 120.98, 120.60, 111.56, 111.43, 111.06, 110.50, 56.15, 56.07, 56.03 ppm.

HRMS m/z [M+H]^+^ calculated for C_22_H_20_N_2_O_4_S: 409.1216; found: 409.1214.

### 4.2 Kinase assays

Compounds were screened for PIKfyve activity using a mobility shift assay (Nanosyn, Santa Clara, CA, USA). Data are expressed as the compound concentration that causes 50% inhibition of the enzymatic activity (IC_50_ values).

The kinase selectivity profile of compound **16i** was determined by measuring the residual activity values at 10 µM in duplicate in 70 kinase assays (66 protein kinases and 4 lipid kinases) and was performed at Reaction Biology, using the “Diversify-Panel.” The list of kinases that have been investigated and the obtained values (percentages of control) are available in the Supplementary Material.

### 4.3 Molecular modeling

Autodock Vina (Eberhardt et al., 2021) was used for docking of compounds **2** and **17a** in the ATP binding site of the PIKfyve structure (PDB 7K2V) (Lees et al., 2020). As this structure has a resolution of 6.60 Å, we opted for induced fit of the ligands by flexible docking, with some active-site amino acid side chains also treated as flexible: Phe1866, Lys1877, Met1937, and Phe1941. Molecular docking of the reference compound **RMC-113** was used to calibrate the docking protocol and to reproduce previous docking results (added polar hydrogens, Vina scoring function, docking box center E1938.O, and box size 70 × 70 × 70 Å) (Karim et al., 2025). The top five score docking hits were selected, and additional molecular dynamic (MD) simulations were performed to verify the stability of the complexes. Those simulations were performed using the Amber25 package (Case et al., 2023). The protein force field was ff19SB. For the ligands, the gaf2 force field was used, and explicit solvent water molecules were modeled as Optimal Point Charge (OPC). Unrestrained MD simulations were performed for 10 ns at 300K.

### 4.4 SARS-CoV-2 assays

#### 4.4.1 Cell lines

Calu-3 cells were maintained in Dulbecco’s modified Eagle’s medium (DMEM, Corning) supplemented with 10% fetal bovine serum (FBS, Omega Scientific, Inc), 1% L-glutamine, 1% penicillin–streptomycin (Pen–Strep), 1% nonessential amino acids (NEAA, Gibco), 1% HEPES (Gibco), and 1% sodium pyruvate (Thermo Fisher Scientific).

Vero E6/TMPRSS2 (JCRB cell bank, #cat JCRB1819) cells were grown in DMEM (Corning) supplemented with 10% FBS (Omega Scientific, Inc), 1% L-glutamine (Gibco), 1% Pen–Strep (Gibco), and 1 mg/mL G418 (Gibco, #10131035). Vero E6 (ATCC) cells were maintained in DMEM supplemented with 10% FBS (Omega Scientific, Inc), 1% L-glutamine, 1% Pen–Strep, 1% non-essential amino acids (Corning), 1% HEPES (Gibco), and 1% sodium pyruvate (Thermo Fisher Scientific). All cell lines were maintained in a humidified incubator with 5% CO_2_ at 37 °C and tested negative for *mycoplasma* using MycoAlert (Lonza, Morristown, NJ). Cells from passages 14–15 were used for this study.

#### 4.4.2 Virus constructs

Plasmids used to produce SARS-CoV-2 (SARS-CoV-2-expressing nLuc-reporter gene) were a gift from Dr. Luis Martinez-Sobrino (Chiem et al., 2021).

#### 4.4.3 Virus production

Viral stock for SARS-CoV-2-nLuc was generated as previously described (Saul et al., 2023) and sequenced. Virus produced in Vero E6/TMPRSS2 cells and passaged 3–4 times was used for the experiments. The supernatants were collected, clarified, and stored at −80 °C. Viral titers were measured using plaque assays on Vero E6 cells.

#### 4.4.4 Measuring antiviral activity

Calu-3 cells were pretreated with the compounds or DMSO for 1 h prior to infection with SARS-CoV-2-nLuc at a multiplicity of infection (MOI) of 0.05. At 2 h post-infection, the viral inoculum was removed, the cells were washed with warm PBS, and the inhibitors were added back for the duration of the experiment. Viral infection was measured at 24 h post-infection using a nanoluciferase assay. The relative light units (RLUs) were normalized to DMSO-treated cells (set as 100%).

#### 4.4.5 Cell viability assays

Cell viability was measured in virus-infected cells using the AlamarBlue® reagent (Invitrogen) according to the manufacturer’s protocol. Fluorescence was detected at 560 nm using a GloMax Discover Microplate Reader (Promega). The raw fluorescence values were normalized to DMSO-treated cells (set as 100%).

## Data Availability

The original contributions presented in the study are included in the article/Supplementary Material; further inquiries can be directed to the corresponding author.
