# Supplementary material for: Synthesis, structure–activity relationships, and SARS-CoV-2 antiviral activity of 3,5-disubstituted isothiazolo[4,3-b]pyridines as PIKfyve inhibitors
Source: Front Chem. 2026 May 8;14:1777981. doi: 10.3389/fchem.2026.1777981 (PMC13194157; doi:10.3389/fchem.2026.1777981)
Supplement: Supplementary file 1 [file DataSheet1.docx]

Supplementary Material

Synthesis, structure-activity relationships and SARS-CoV-2 antiviral activity of 3,5-disubstituted isothiazolo[4,3-*b*]pyridines as PIKfyve inhibitors

**Ling-Jie Gao^1^, Demian Kalebic^2^, Chieh Wen Lo^3^, Aakriti Gangwal^3^, Do Hoang Nhu Tran^3^, Jef Rozenski^1^, Dominique Schols^4^, Mathy Froeyen^1^, Wim Dehaen^2^, Shirit Einav^3,5^, Steven De Jonghe^4^**

^1^ Laboratory of Medicinal Chemistry, Department of Pharmaceutical and Pharmacological Sciences, Rega Institute for Medical Research, KU Leuven, Leuven, Belgium

^2^ Sustainable Chemistry for Metals and Molecules, Department of Chemistry, KU Leuven, Leuven, Belgium.

^3^ Department of Medicine, Division of Infectious Diseases and Geographic Medicine, and Department of Microbiology and Immunology, Stanford University School of Medicine, Stanford, CA 94305, USA

^4^ Molecular, Structural and Translational Virology Research Group, Department of Microbiology, Immunology and Transplantation, Rega Institute for Medical Research, KU Leuven, Leuven, Belgium

^5^ Biohub, San Francisco, California, 94158, USA

*** Correspondence:**Corresponding Author
steven.dejonghe@kuleuven.be

^1^H NMR spectrum of **2**


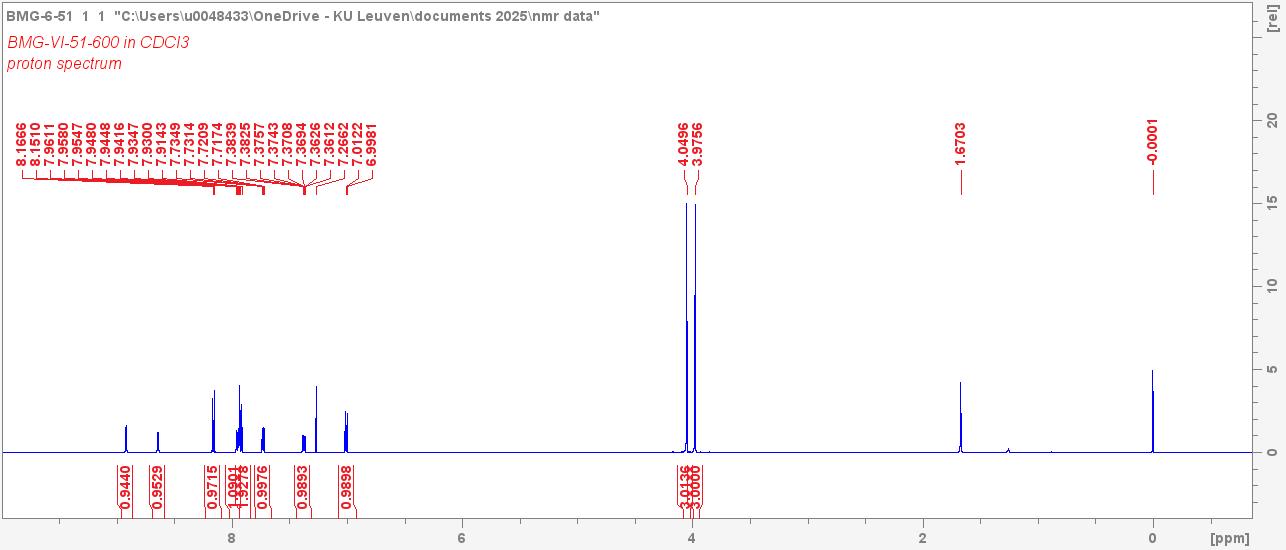


^13^C NMR spectrum of **2**


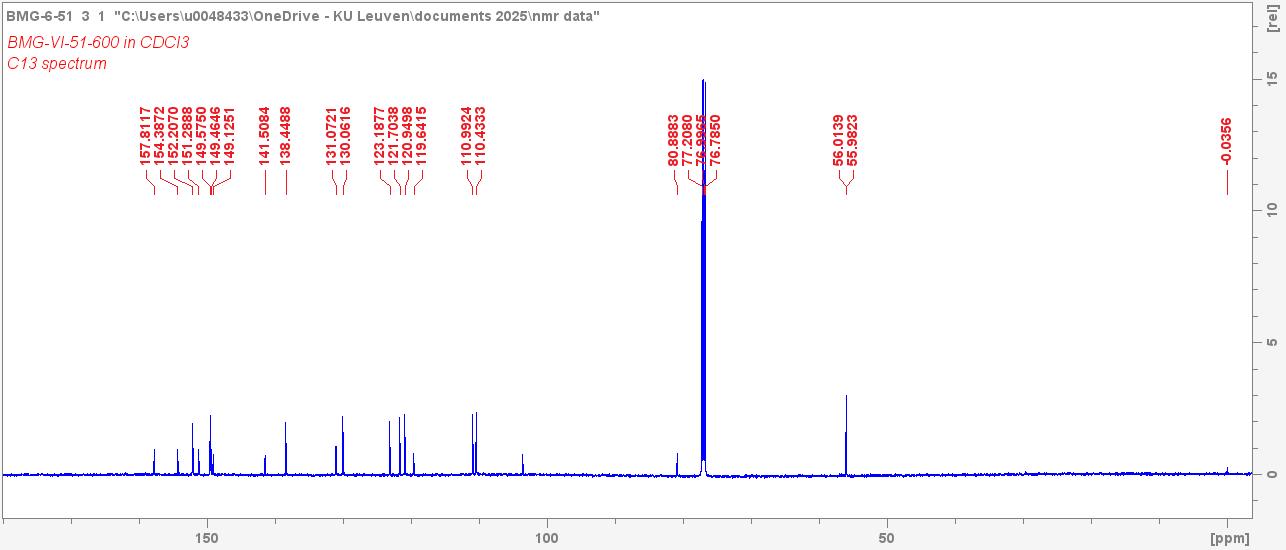


^1^H NMR spectrum of **9**


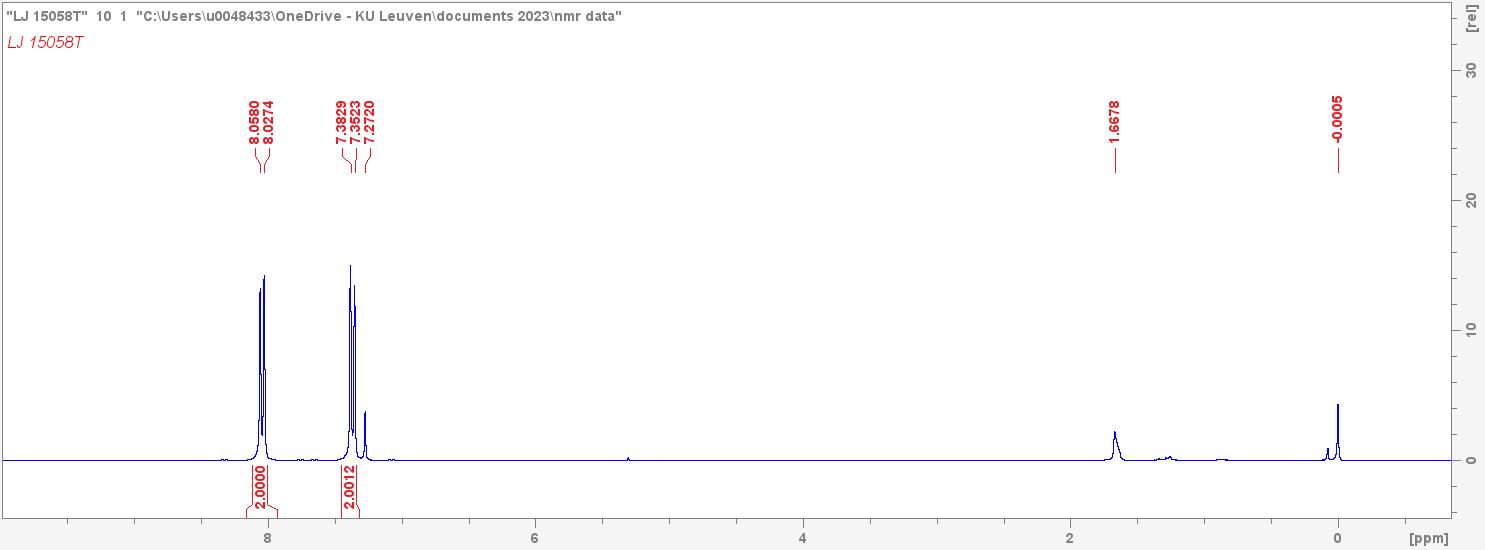


^13^C NMR spectrum of **9**


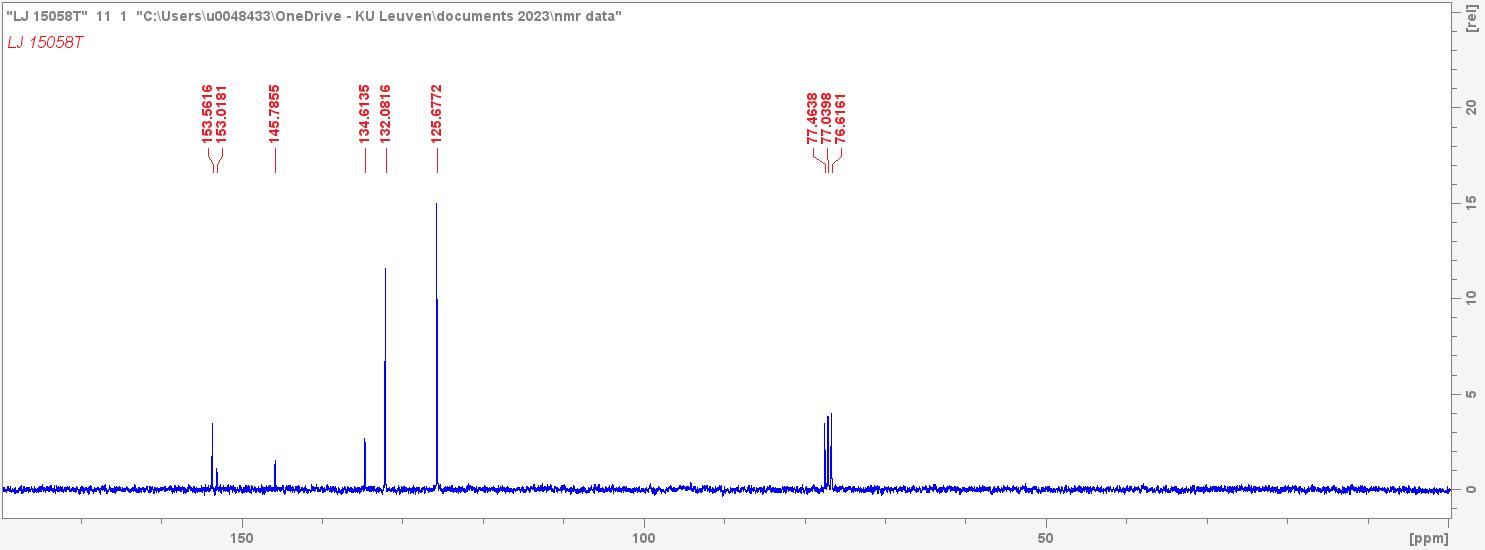


^1^H NMR spectrum of **11**


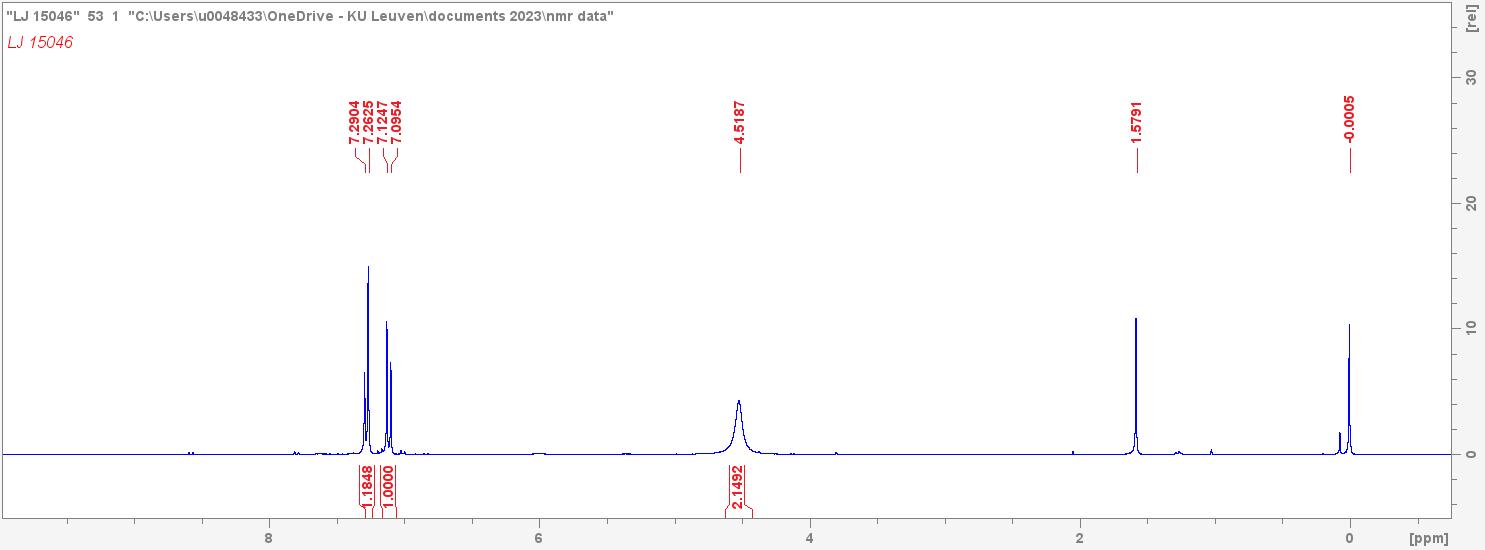


^13^C NMR spectrum of **11**


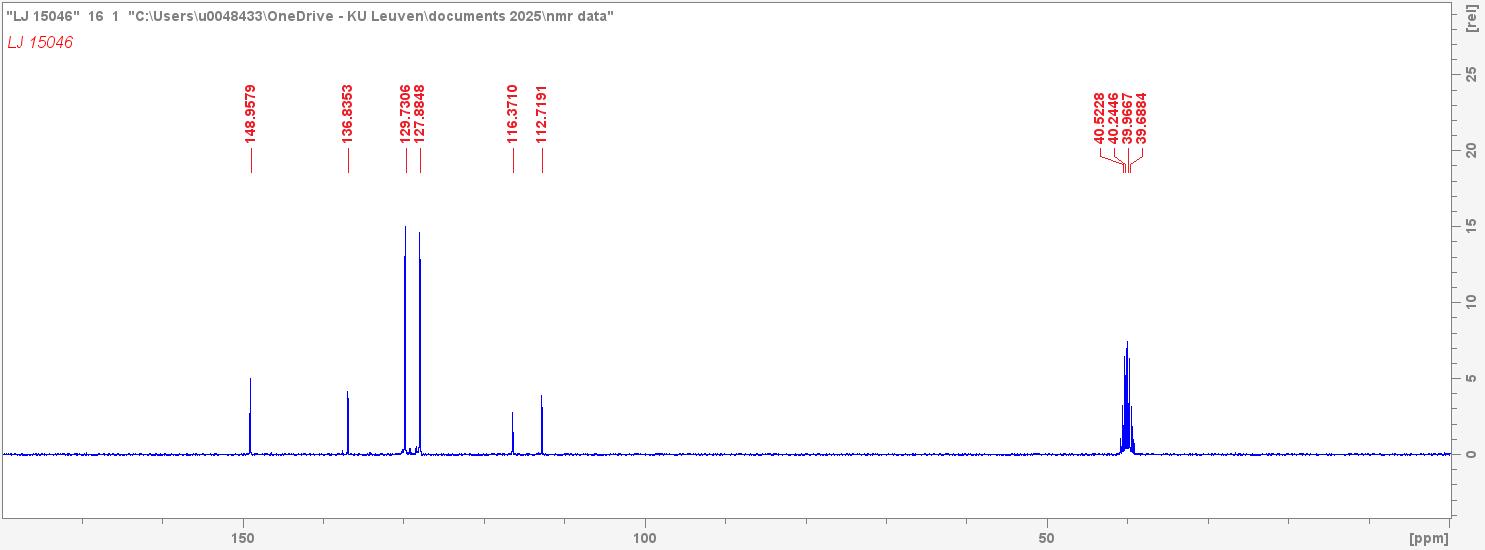


^1^H NMR spectrum of **12**


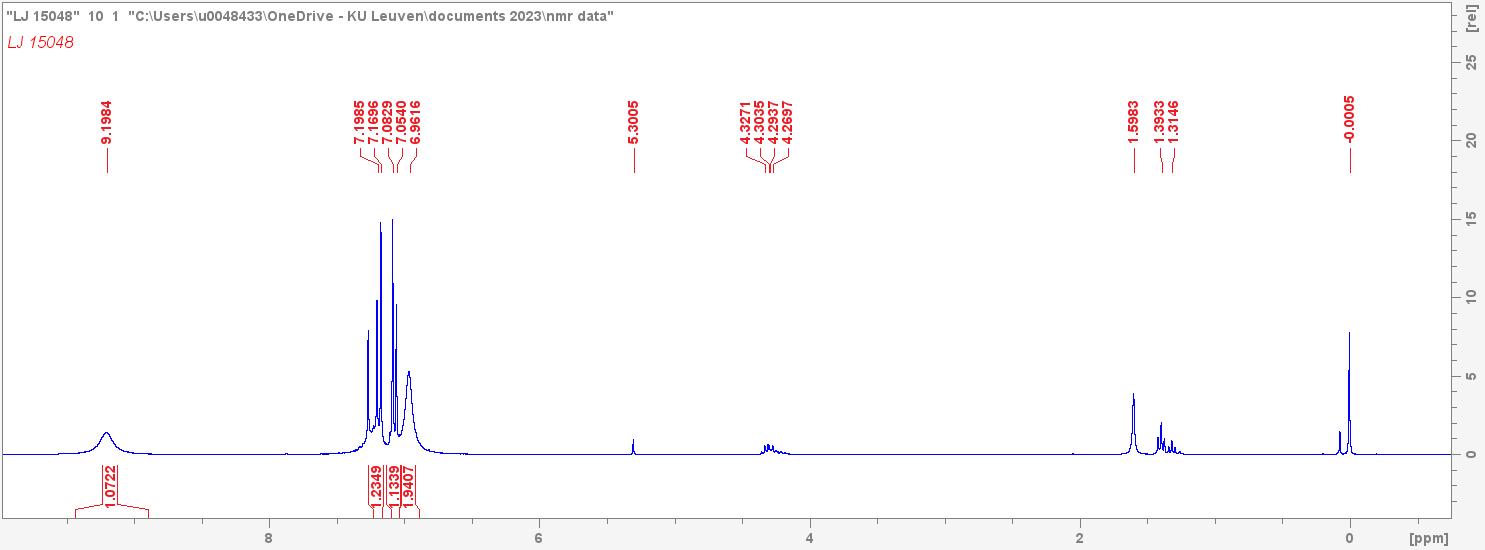


^13^C NMR spectrum of **12**


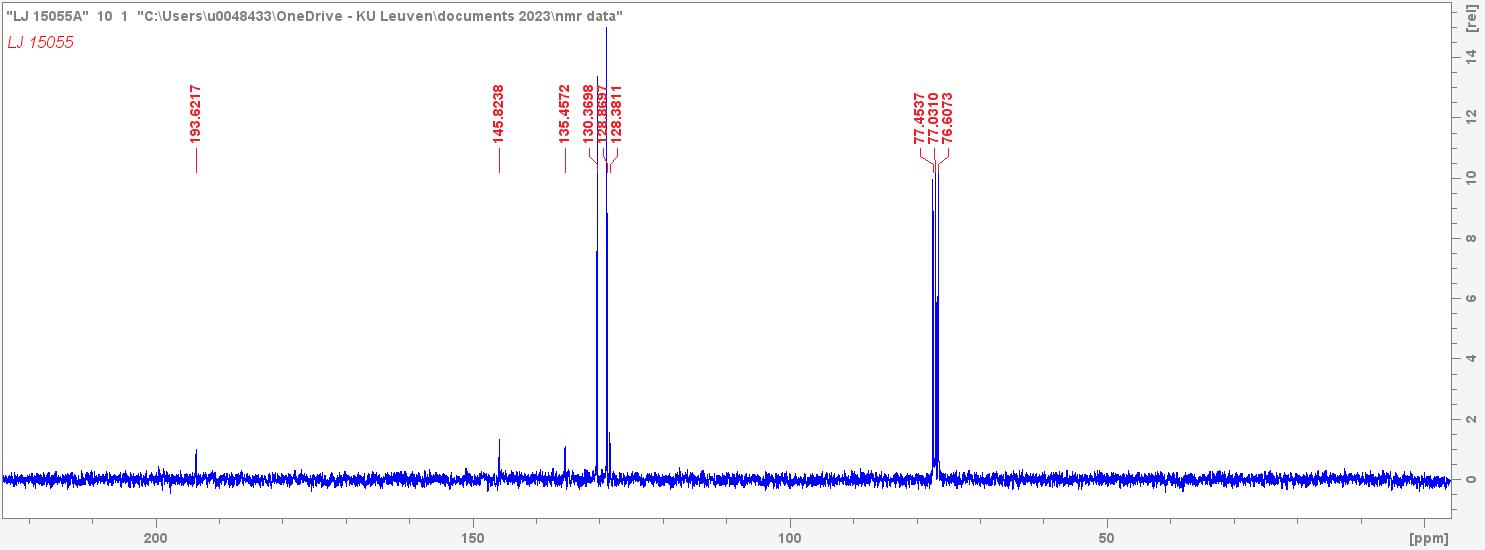


^1^H NMR spectrum of **13**


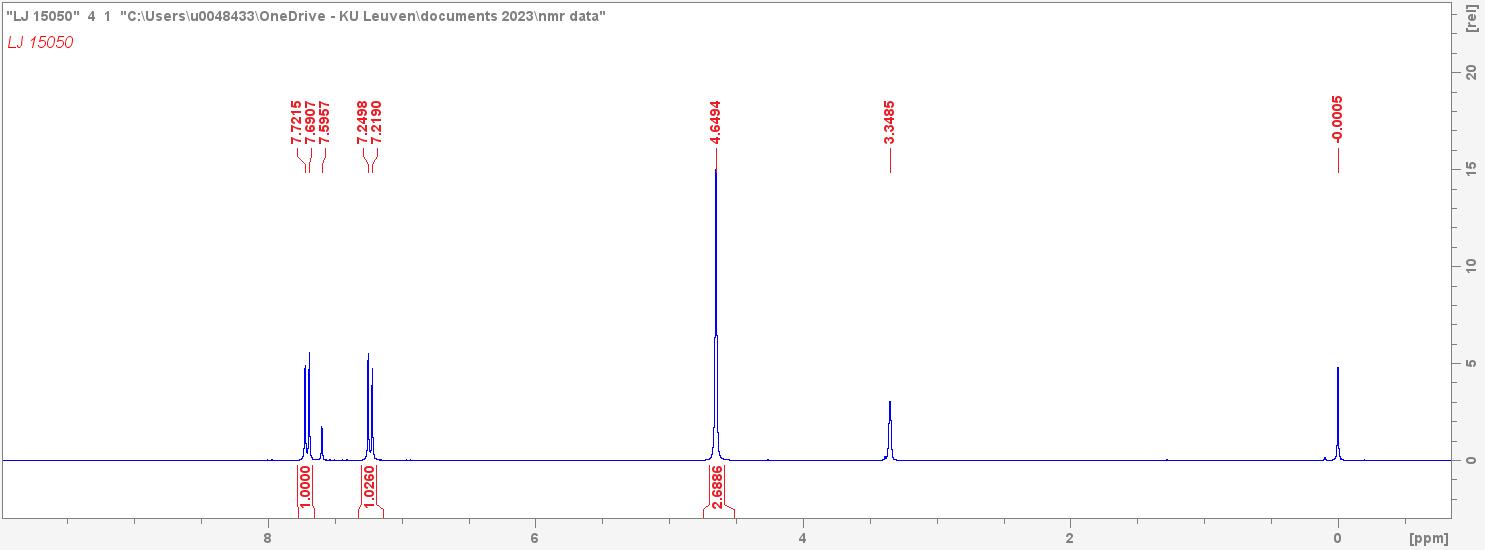


^13^C NMR spectrum of **13**


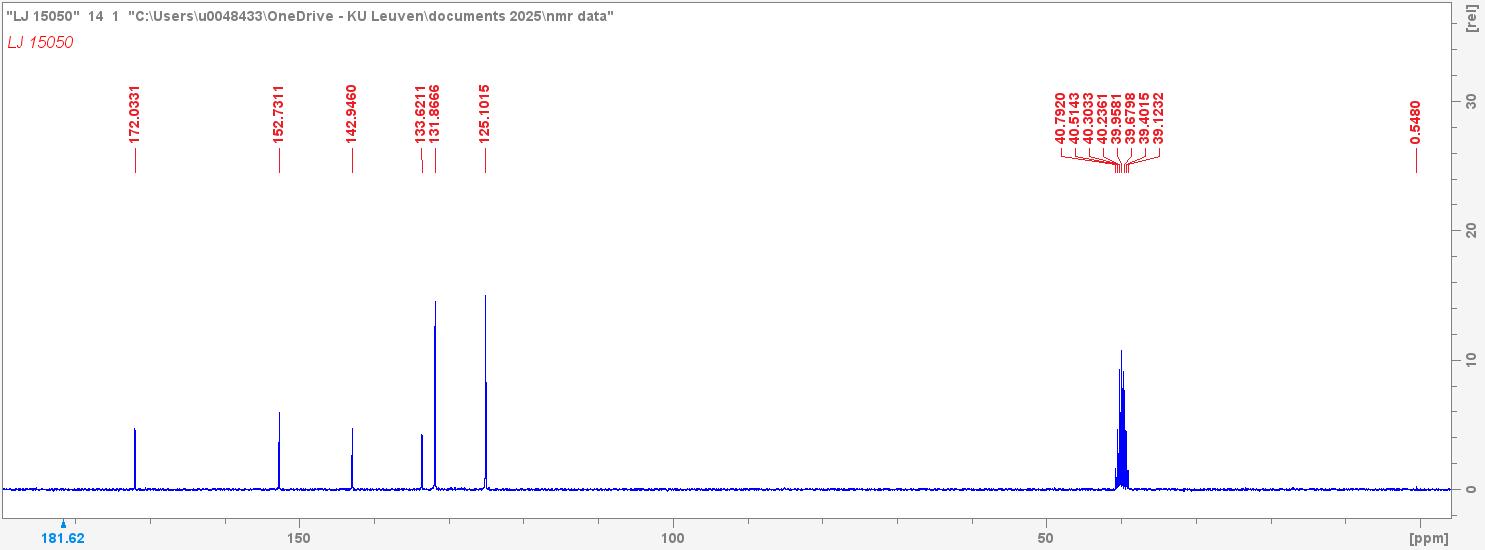


^1^H NMR spectrum of **15a**


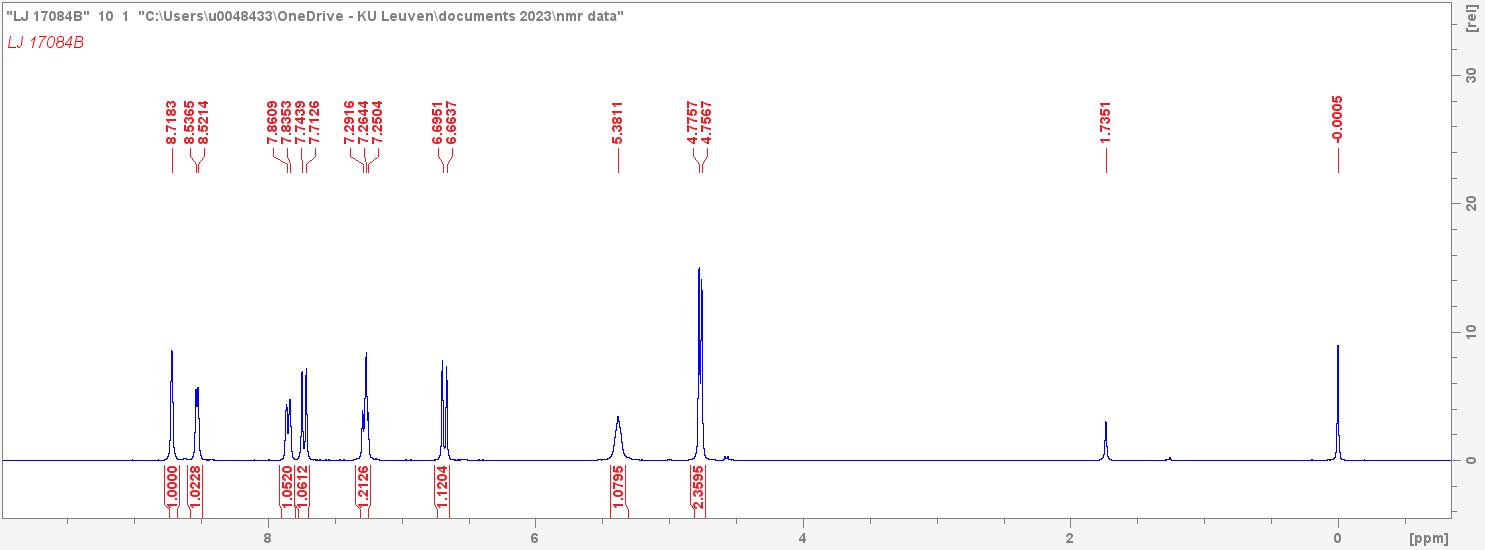


^13^C NMR spectrum of **15a**


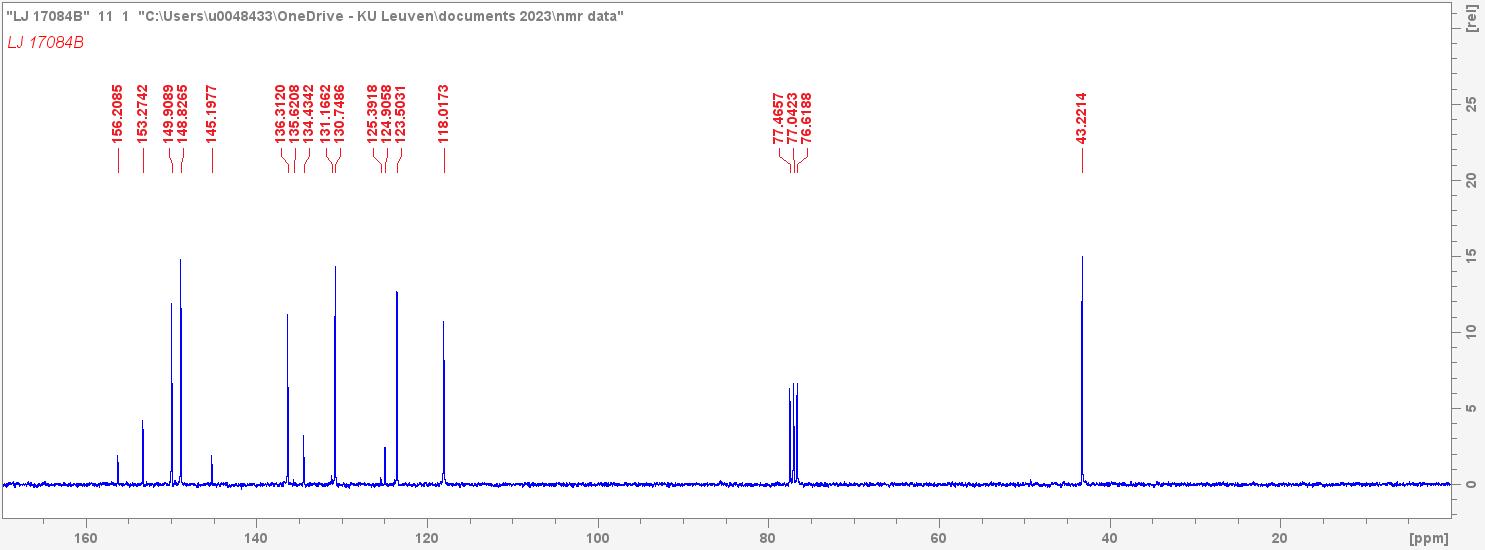


^1^H NMR spectrum of **15b**


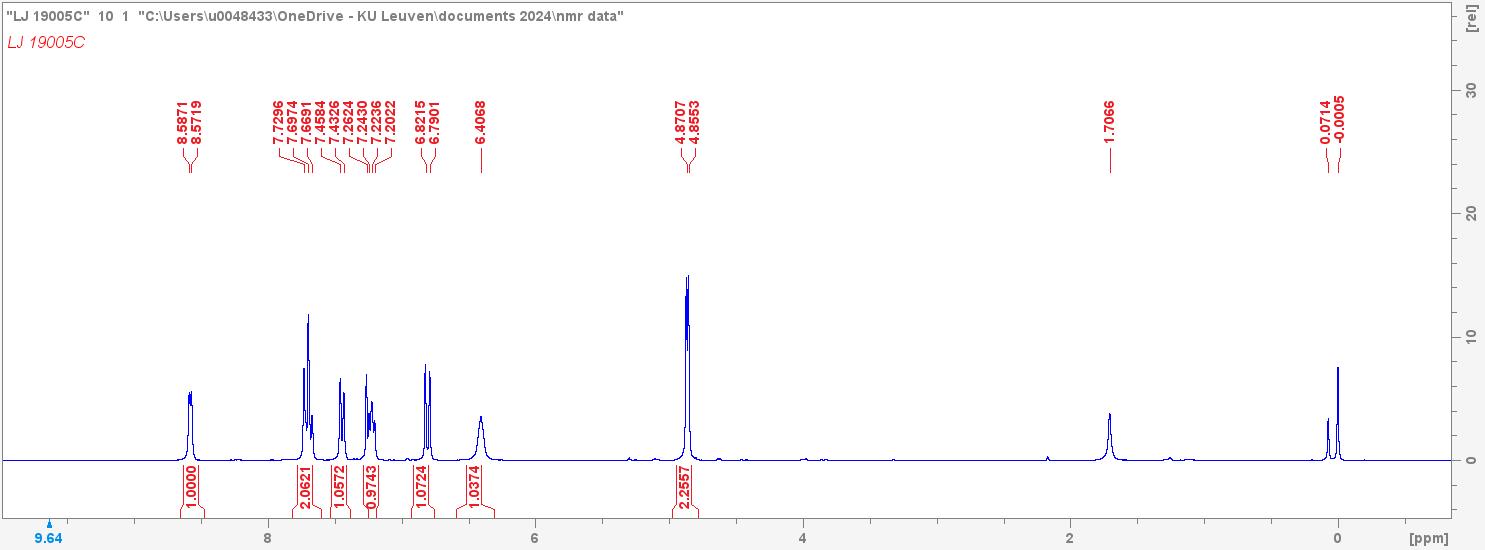


^13^C NMR spectrum of **15b**


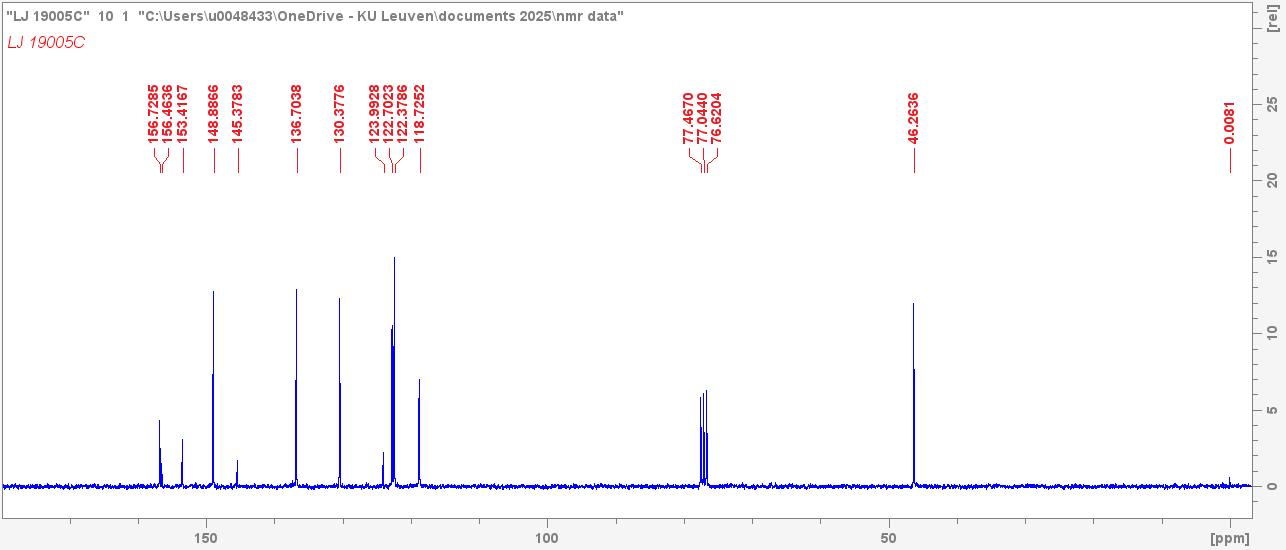


^1^H NMR spectrum of **15c**


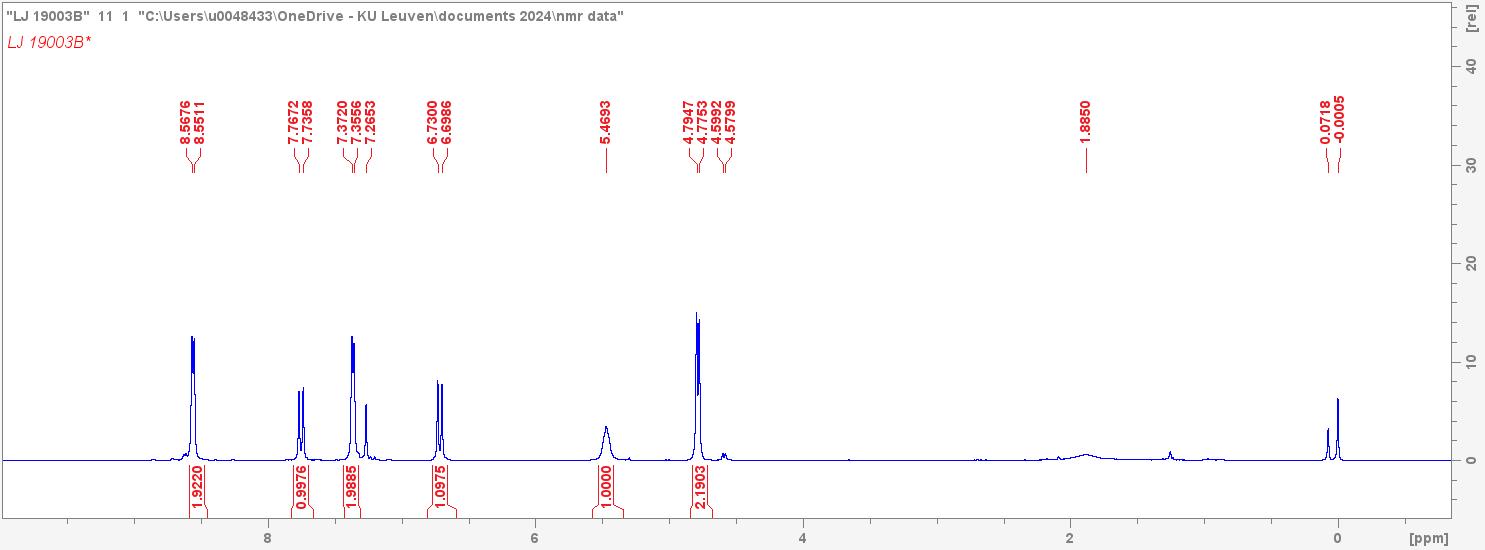


^13^C NMR spectrum of **15c**


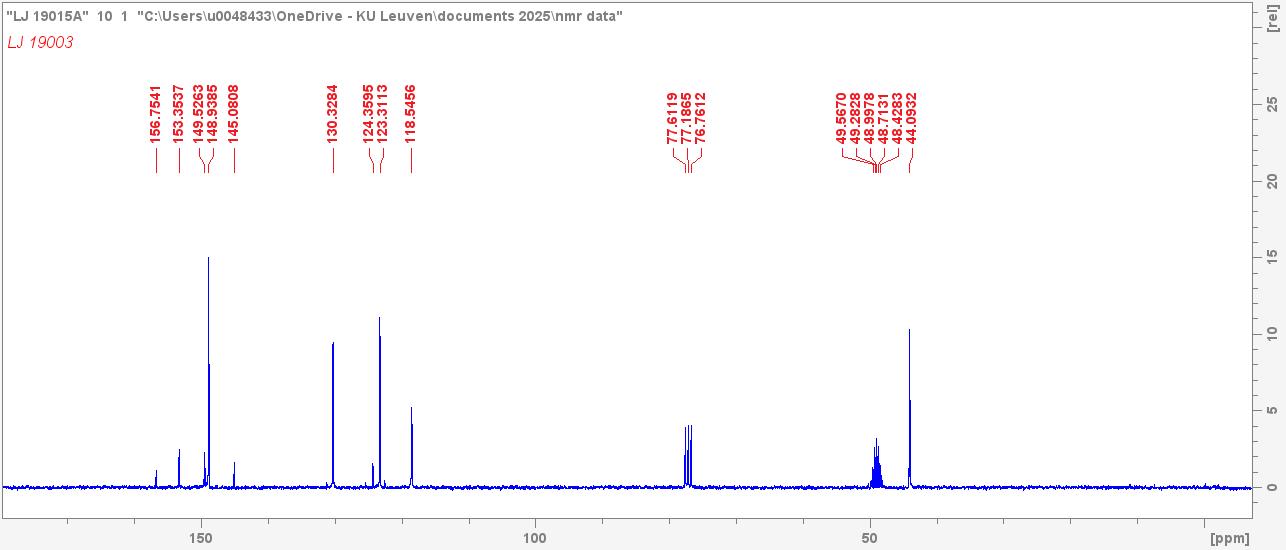


^1^H NMR spectrum of **15d**


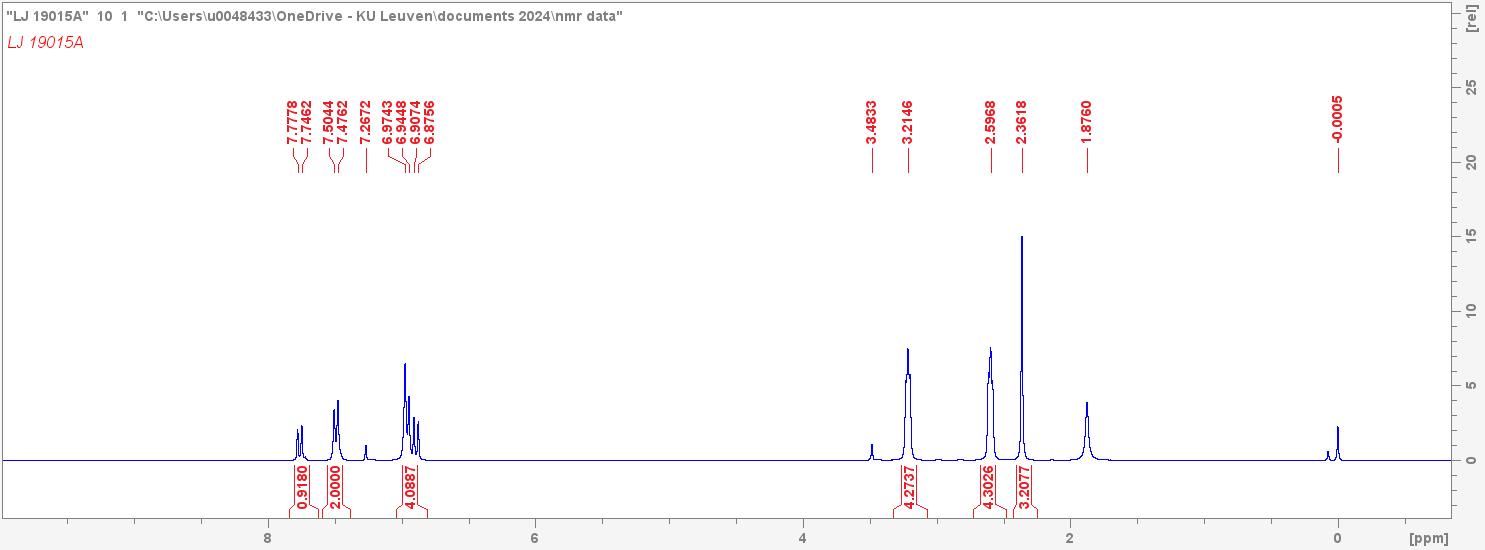


^13^C NMR spectrum of **15d**


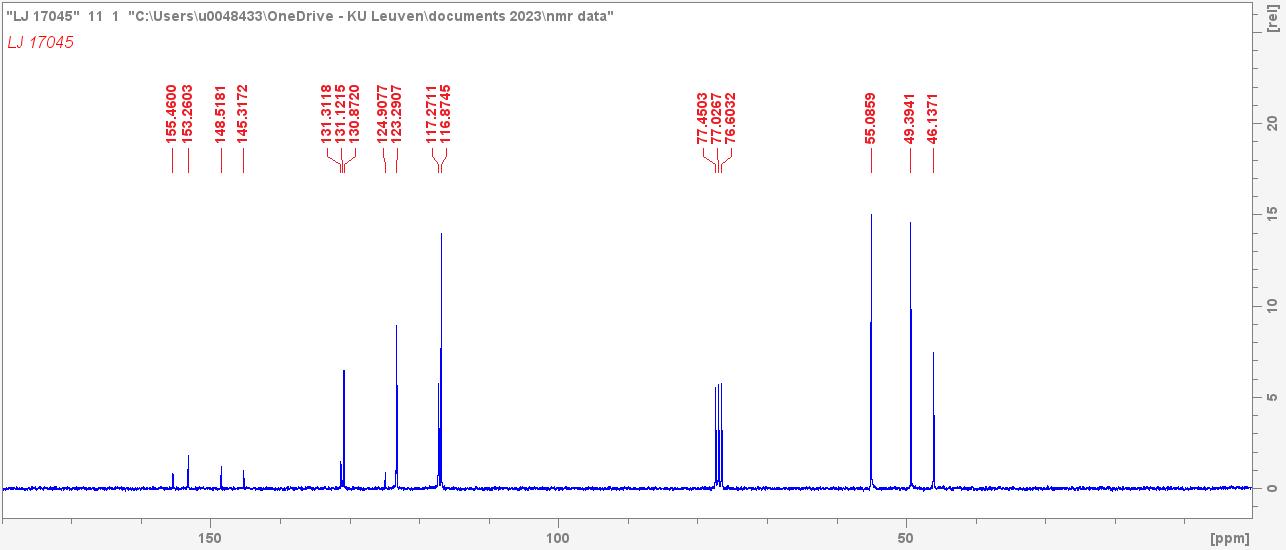


^1^H NMR spectrum of **15e**


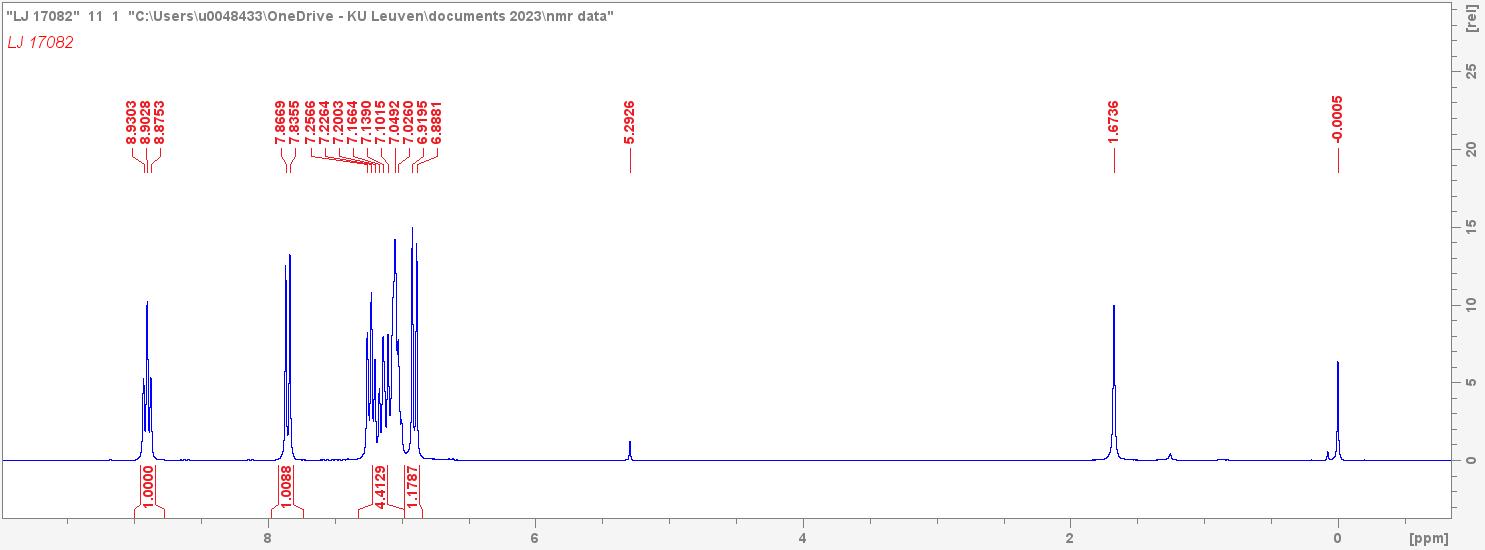


^13^C NMR spectrum of **15e**


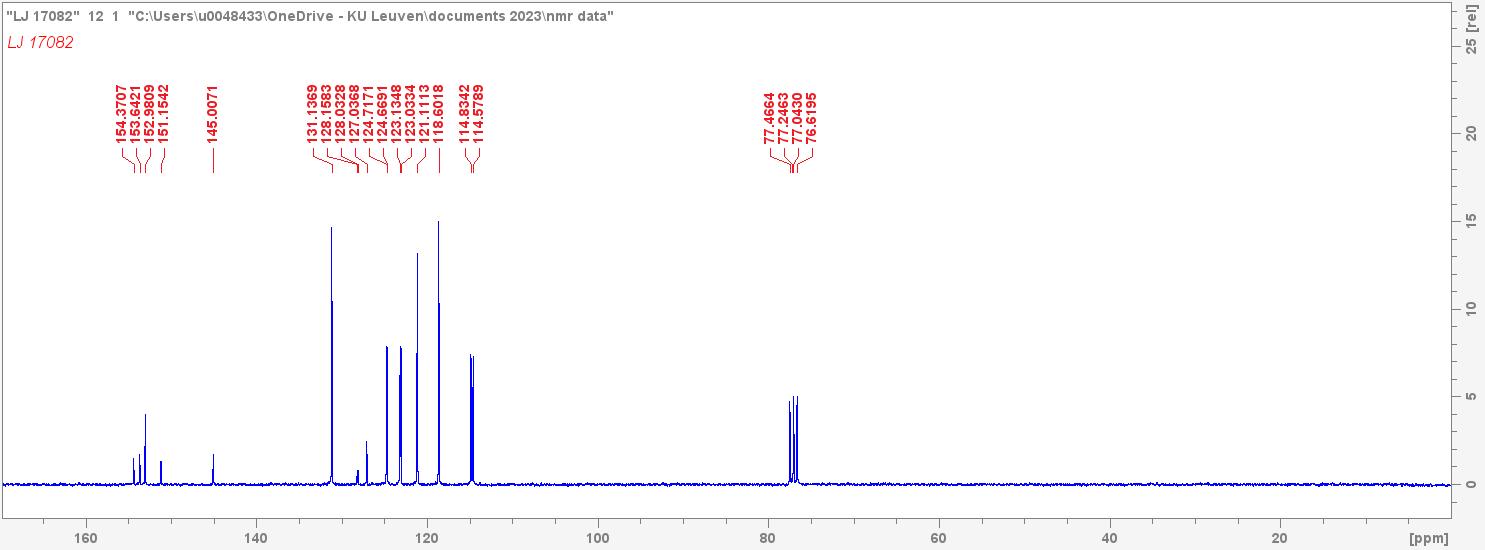


^1^H NMR spectrum of **15f**


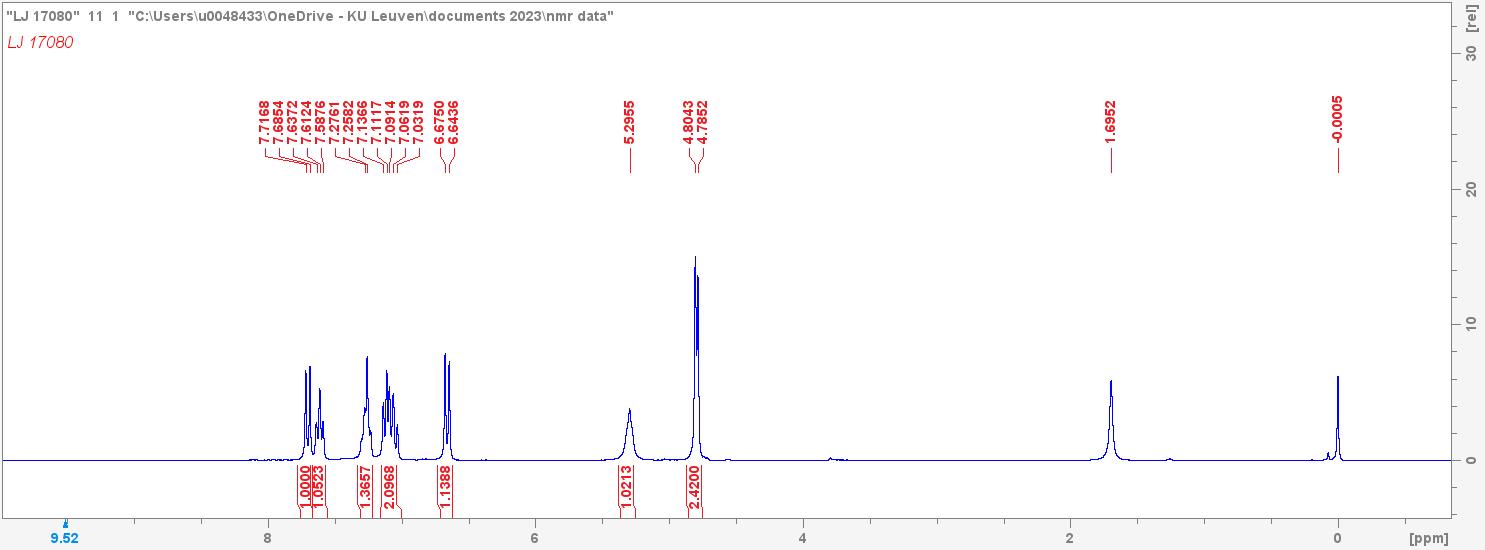


^13^C NMR spectrum of **15f**


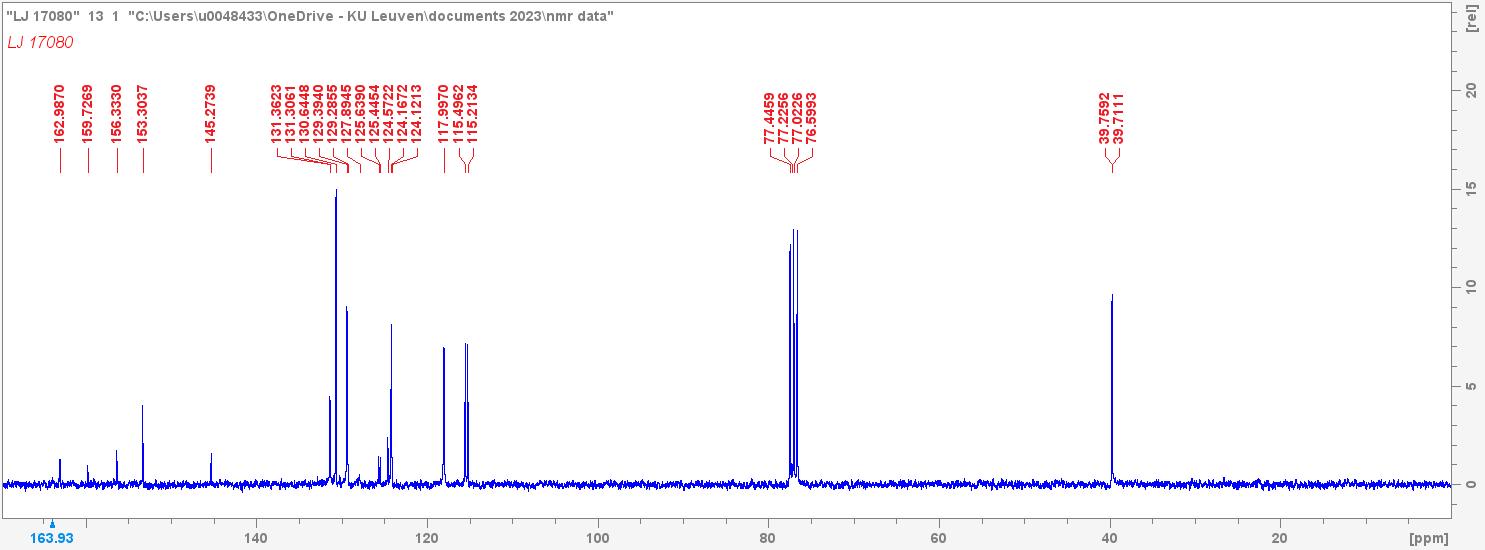


^1^H NMR spectrum of **15g**


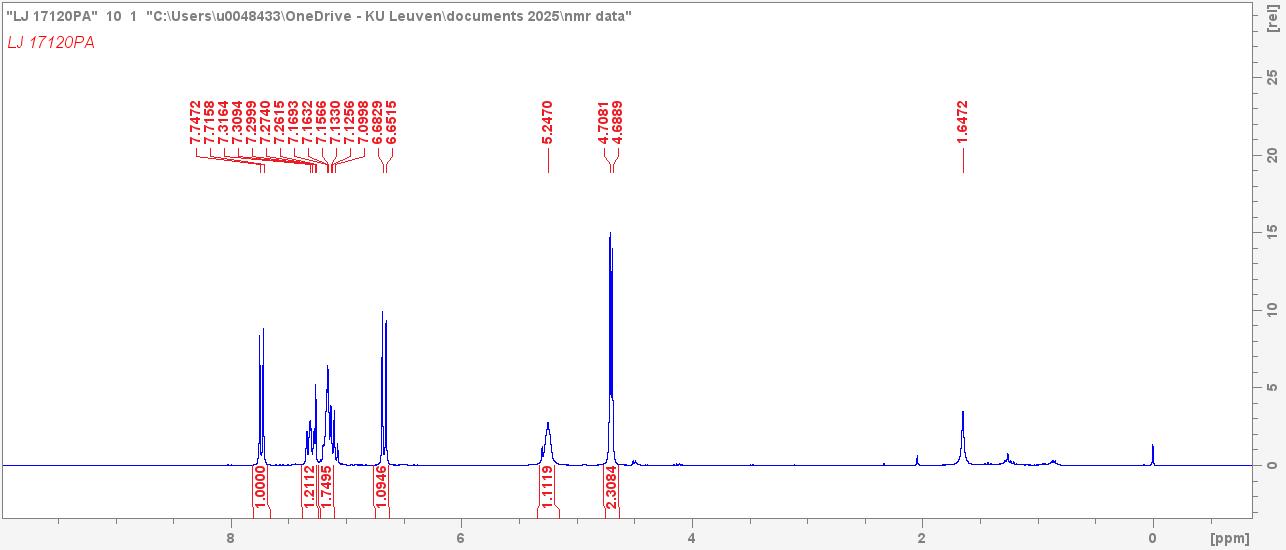


^13^C NMR spectrum of **15g**


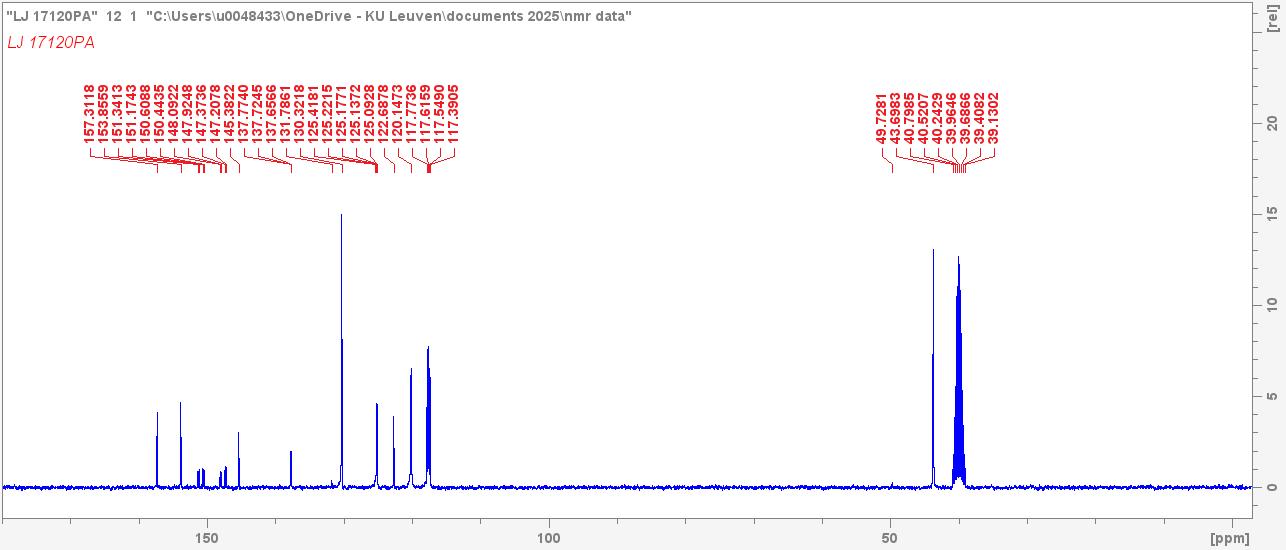


^1^H NMR spectrum of **15h**


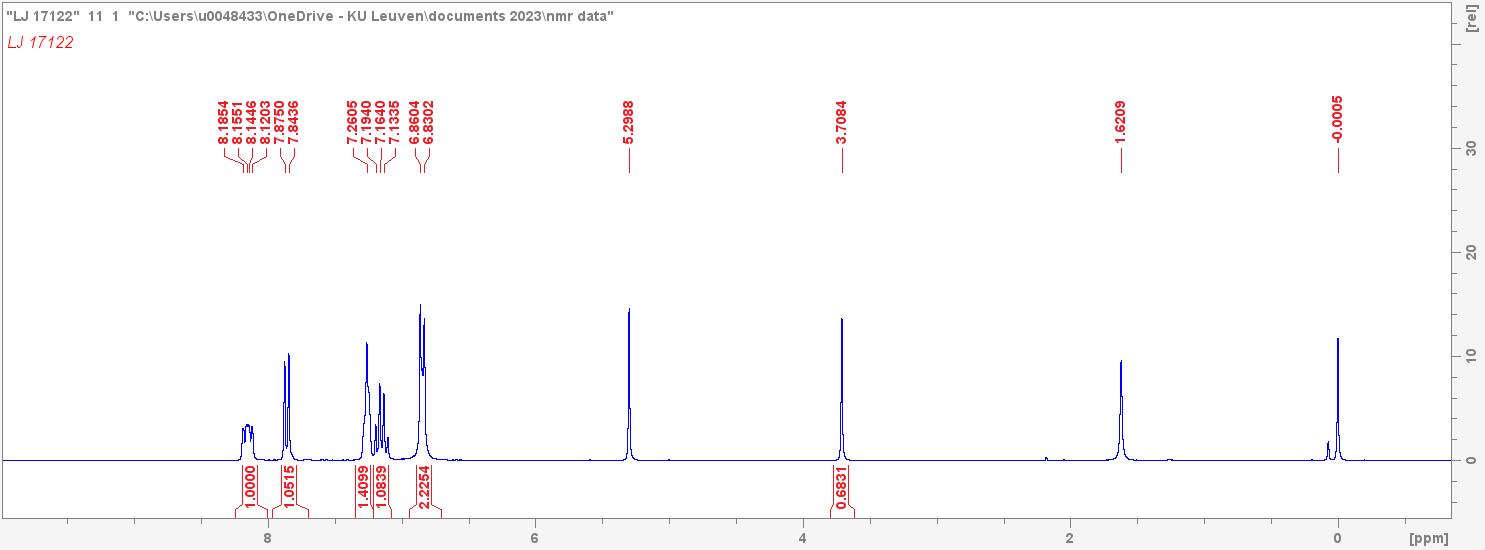


^13^C NMR spectrum of **15h**


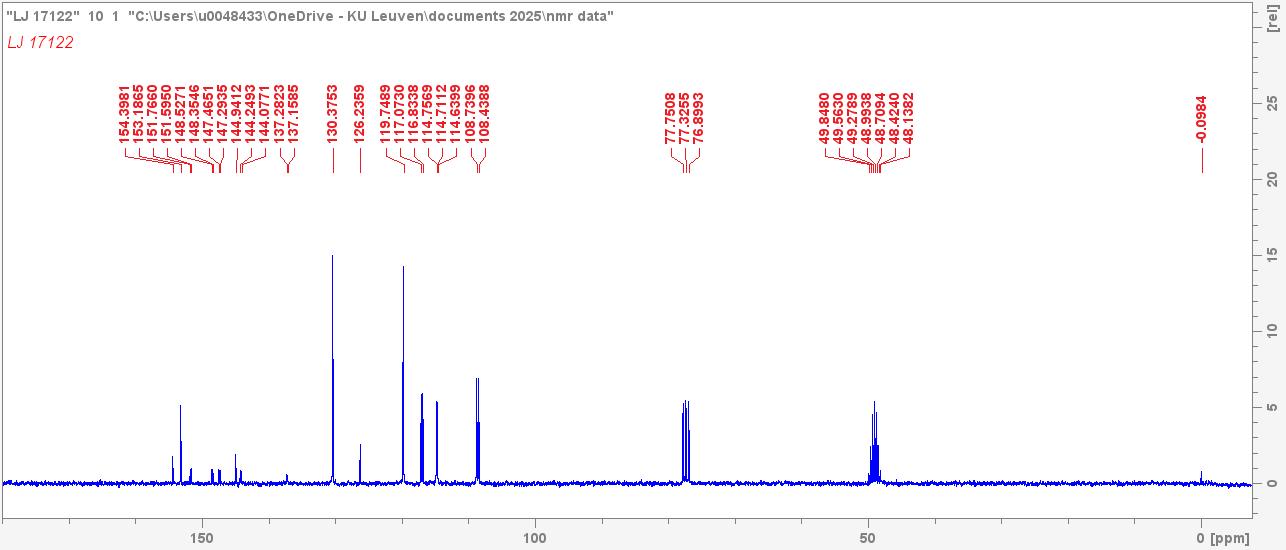


^1^H NMR spectrum of **16a**


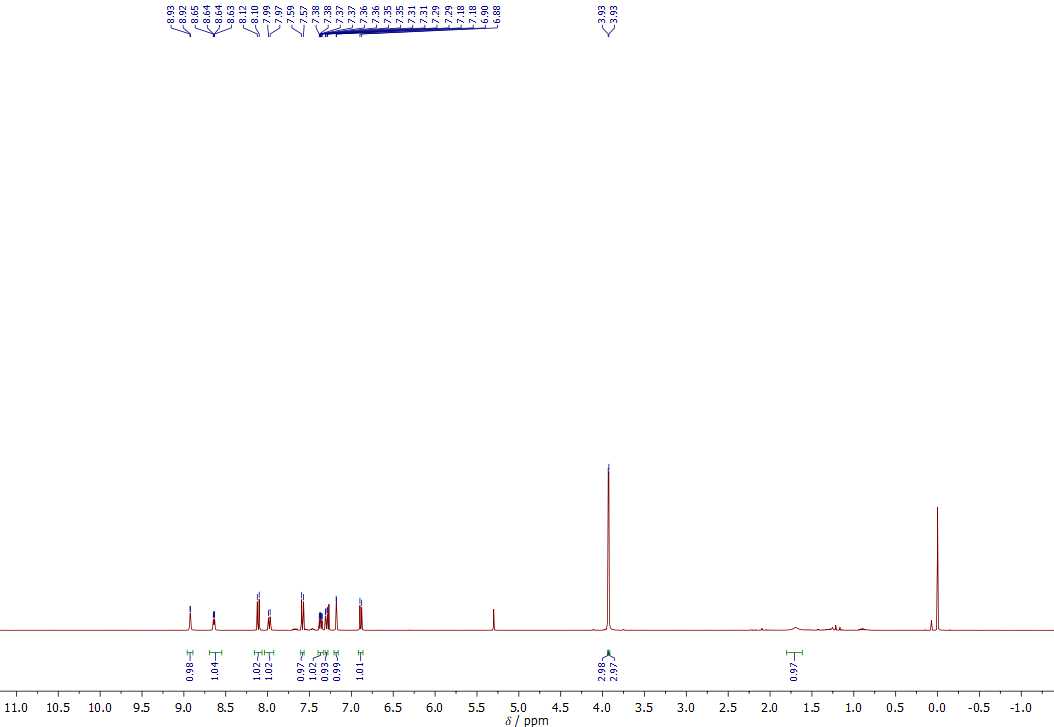


^13^C NMR spectrum of **16a**


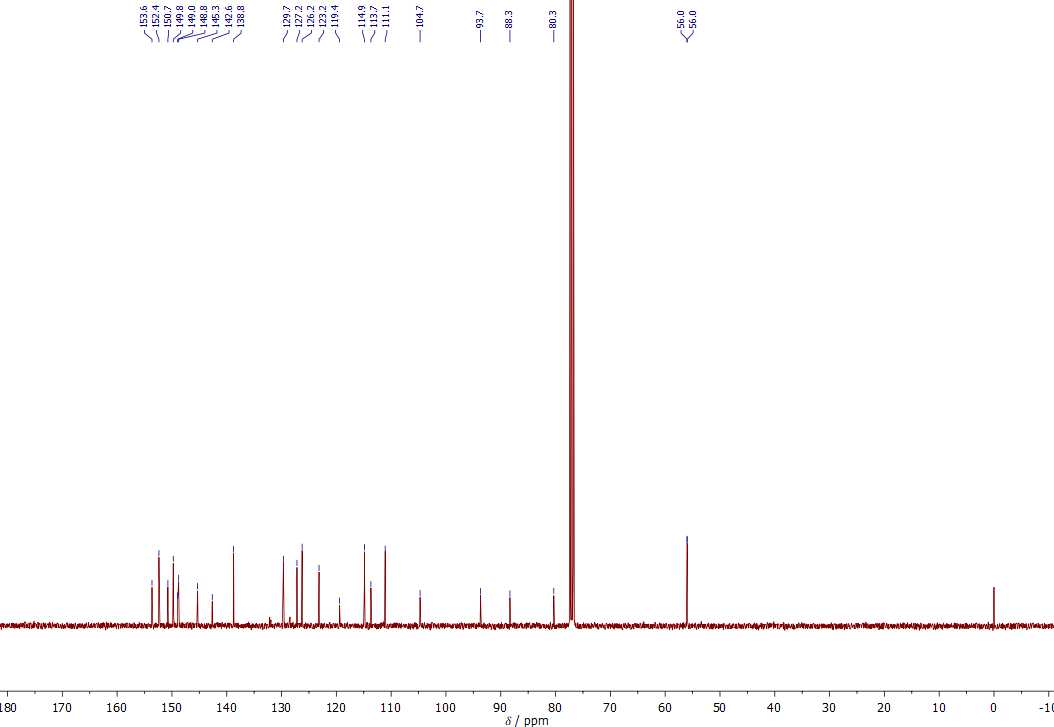


^1^H NMR spectrum of **16b**


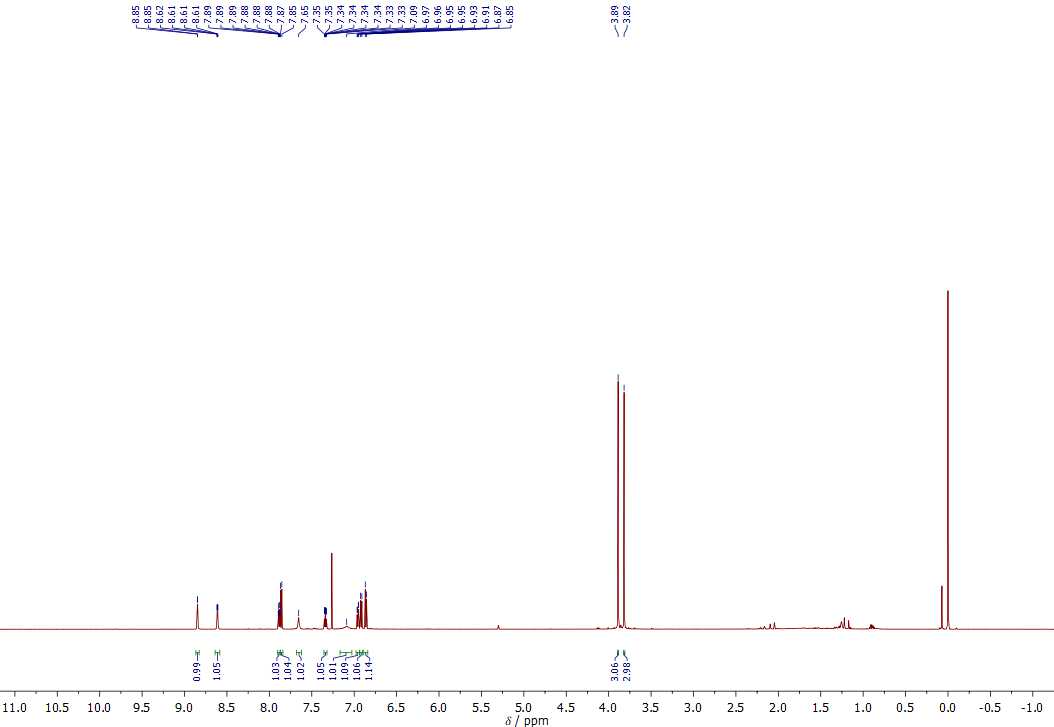


^13^C NMR spectrum of **16b**


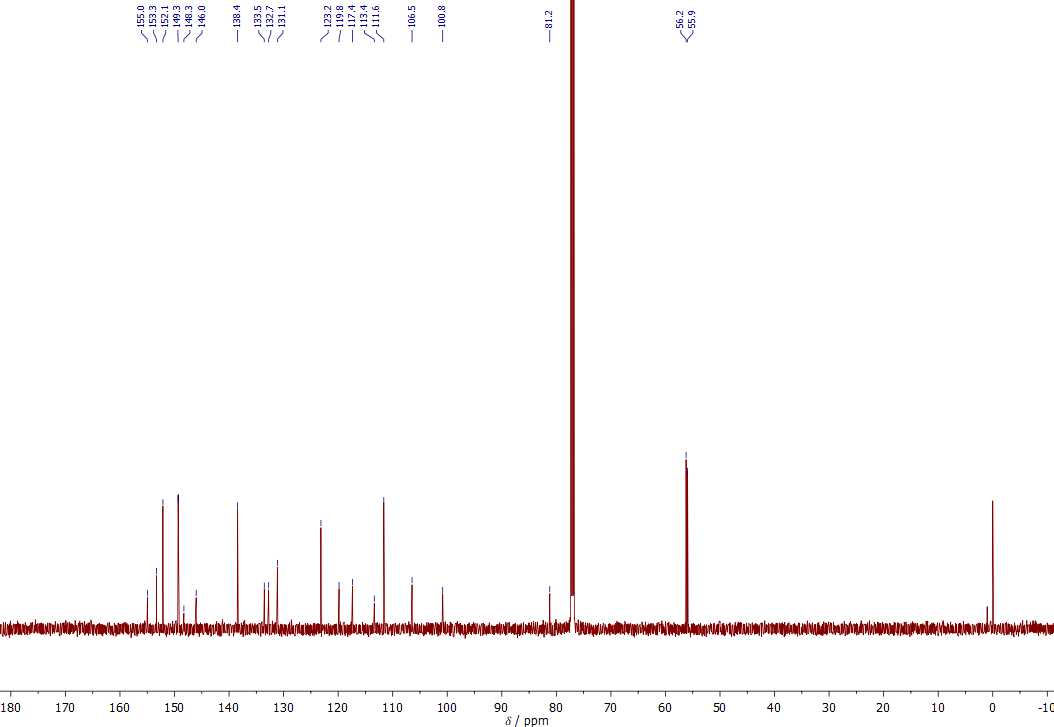


^1^H NMR spectrum of **16c**


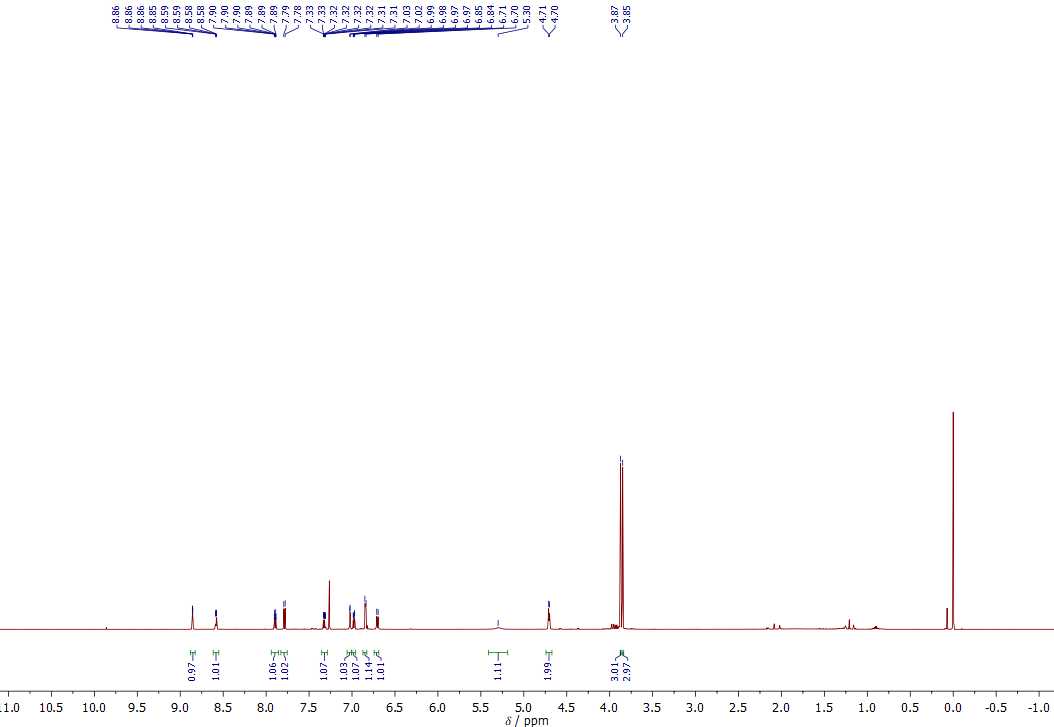


^13^C NMR spectrum of **16c**


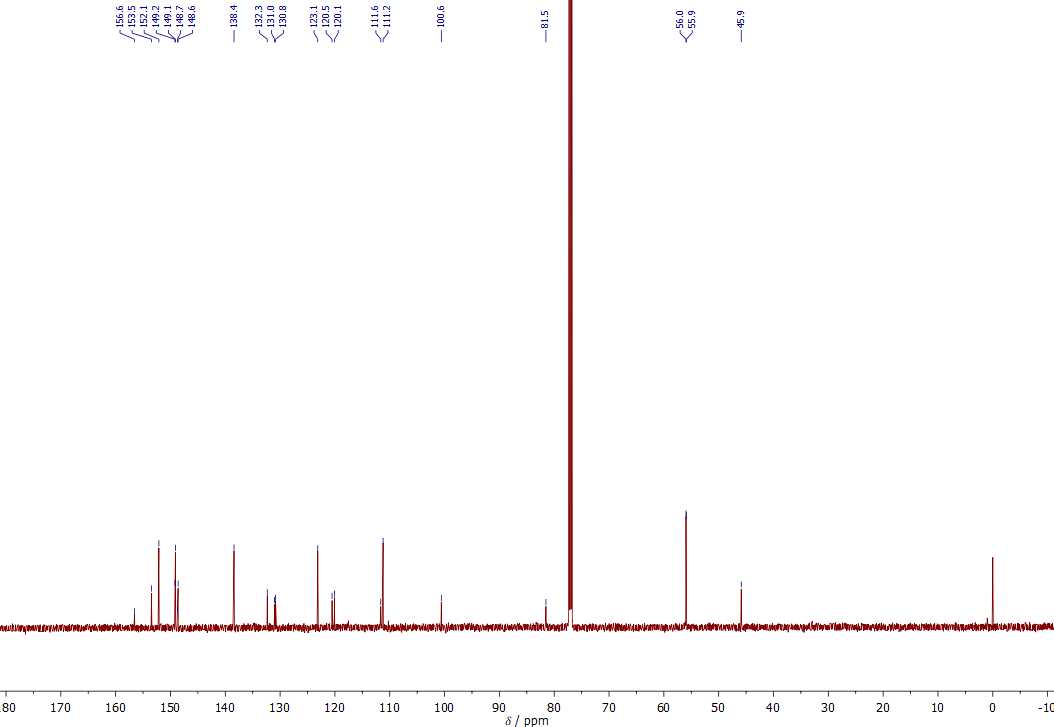


^1^H NMR spectrum of **16d**


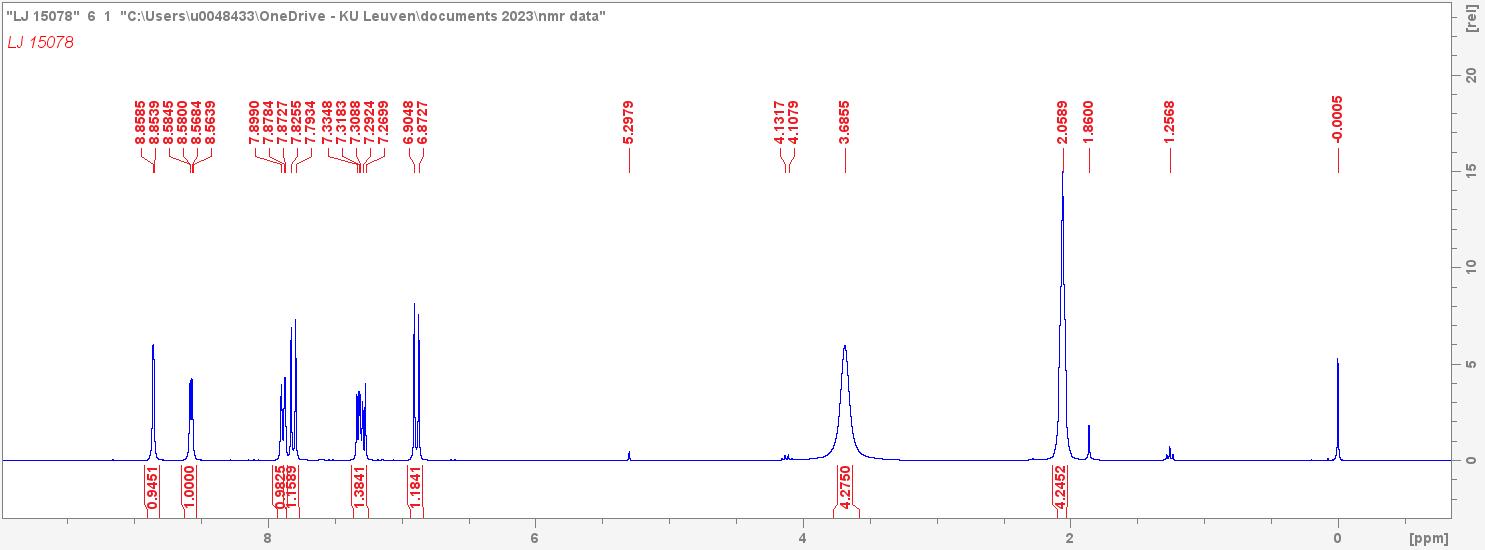


^13^C NMR spectrum of **16d**


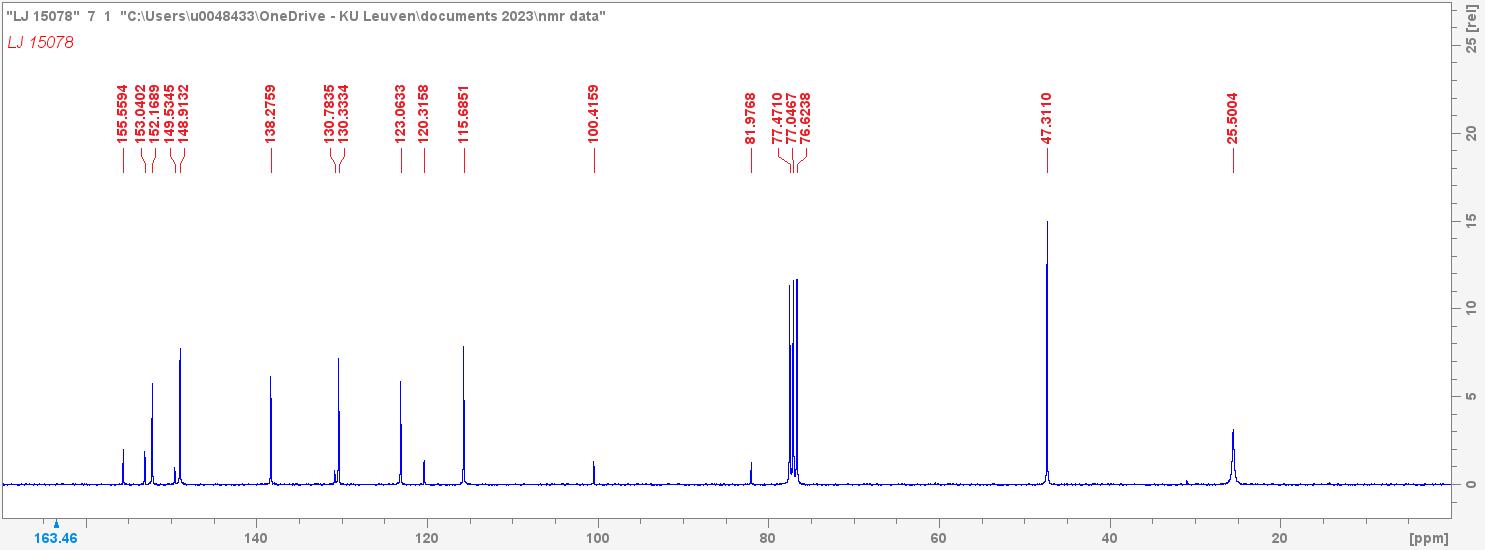


^1^H NMR spectrum of **16e**


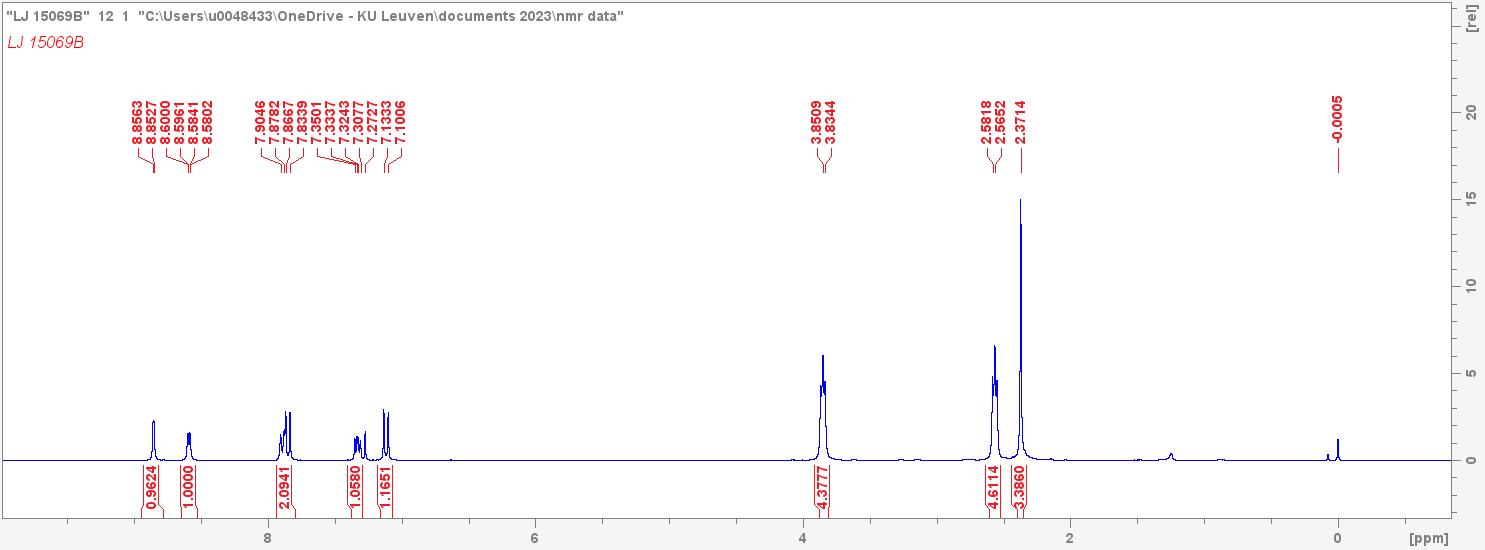


^13^C NMR spectrum of **16e**


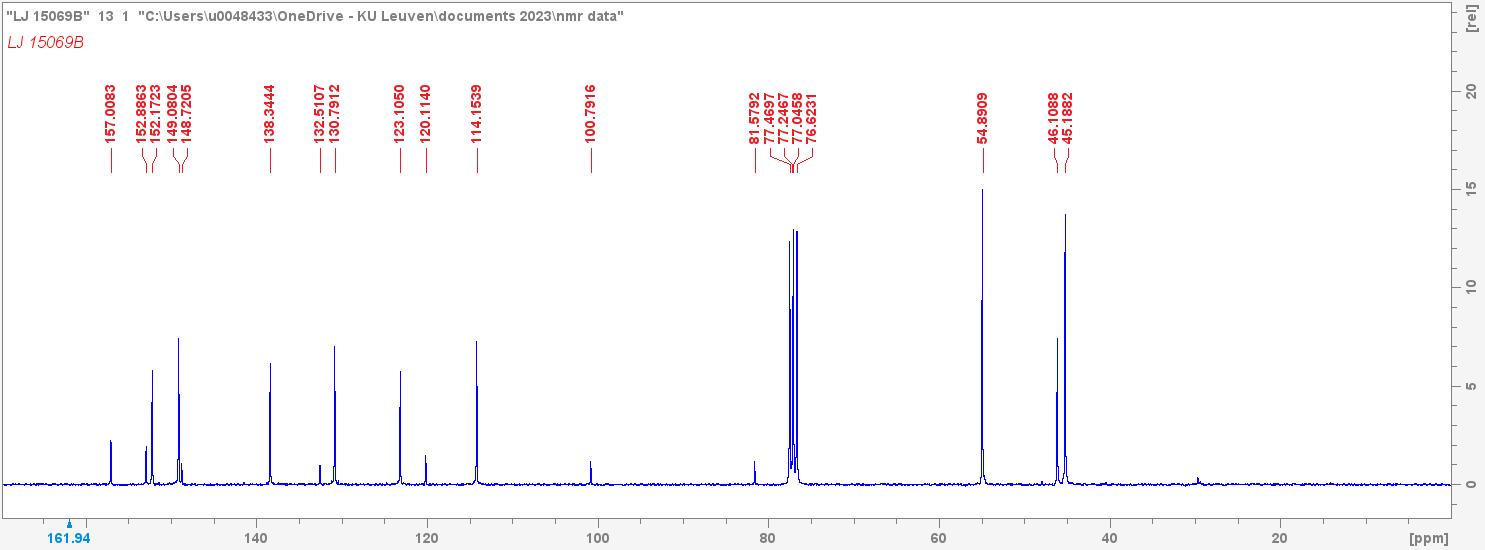


^1^H NMR spectrum of **16f**


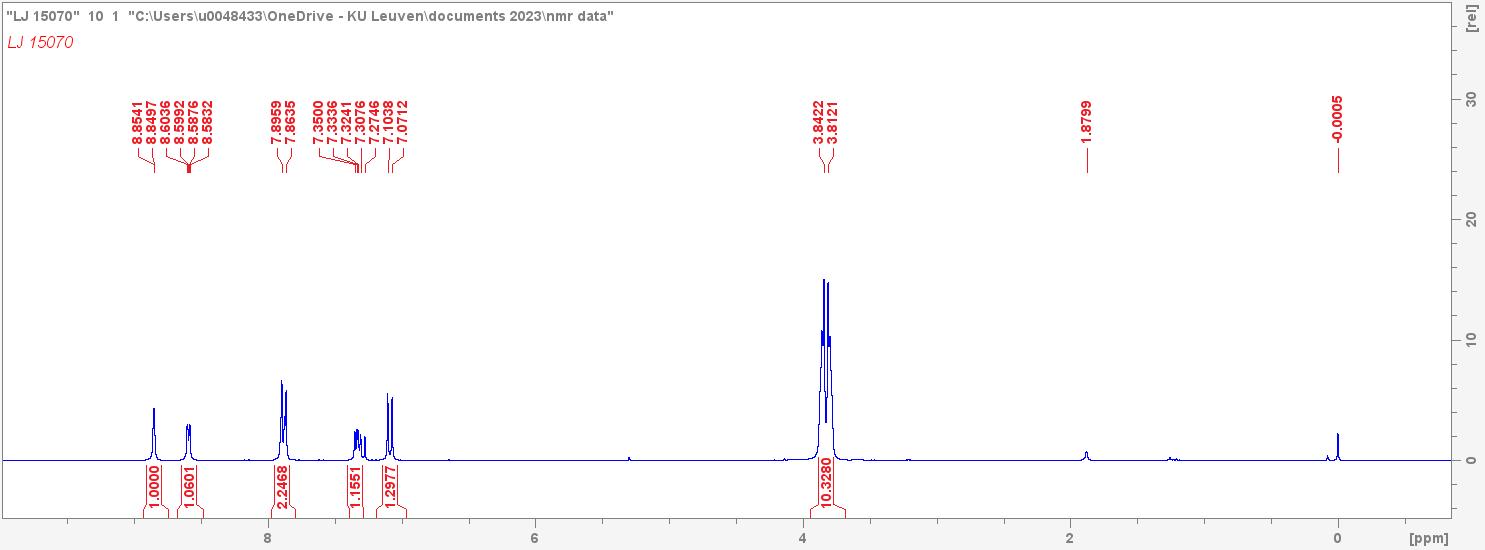


^13^C NMR spectrum of **16f**


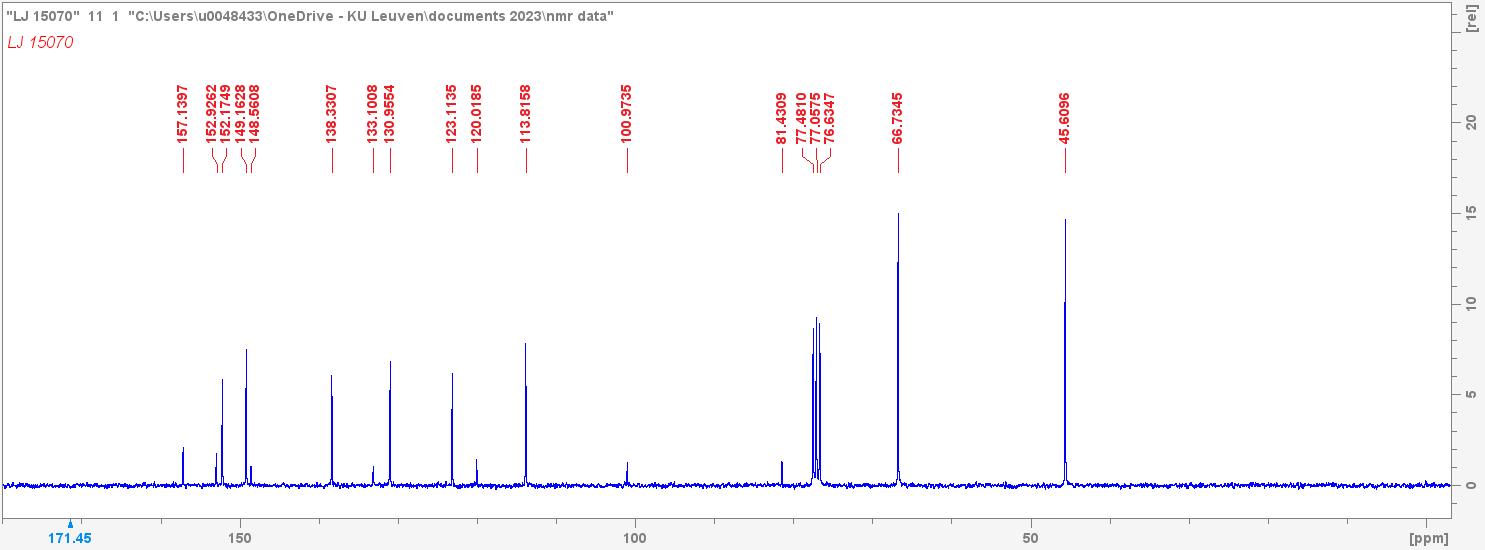


^1^H NMR spectrum of **16g**


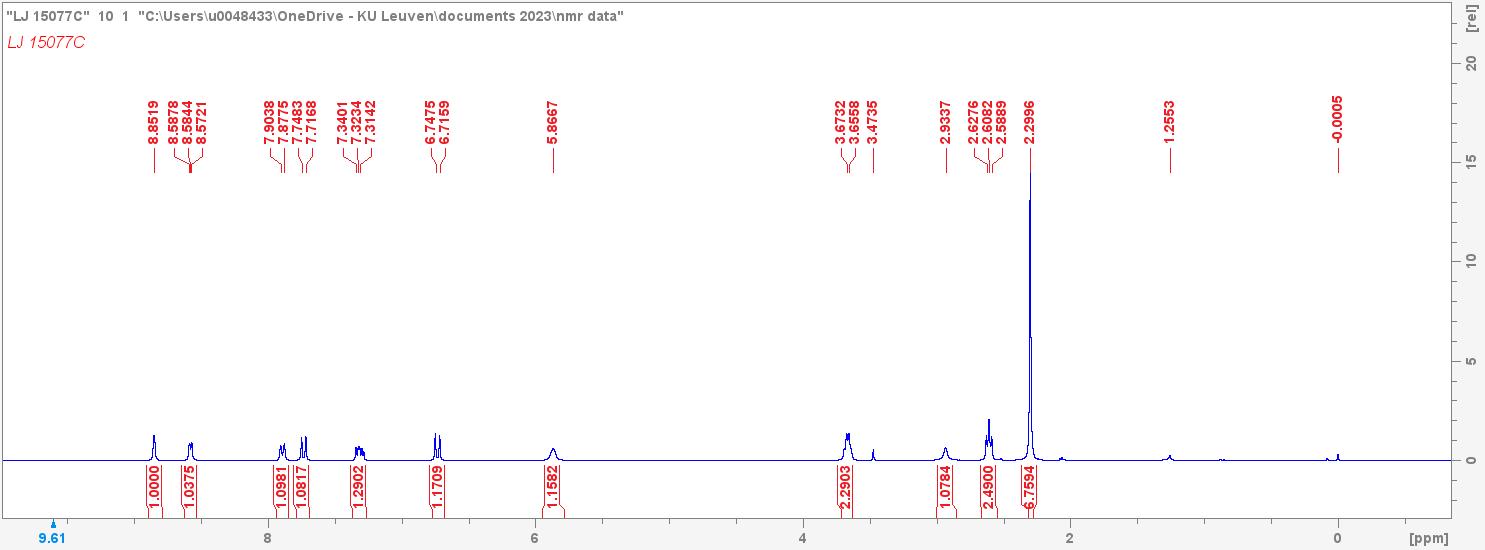


^13^C NMR spectrum of **16g**


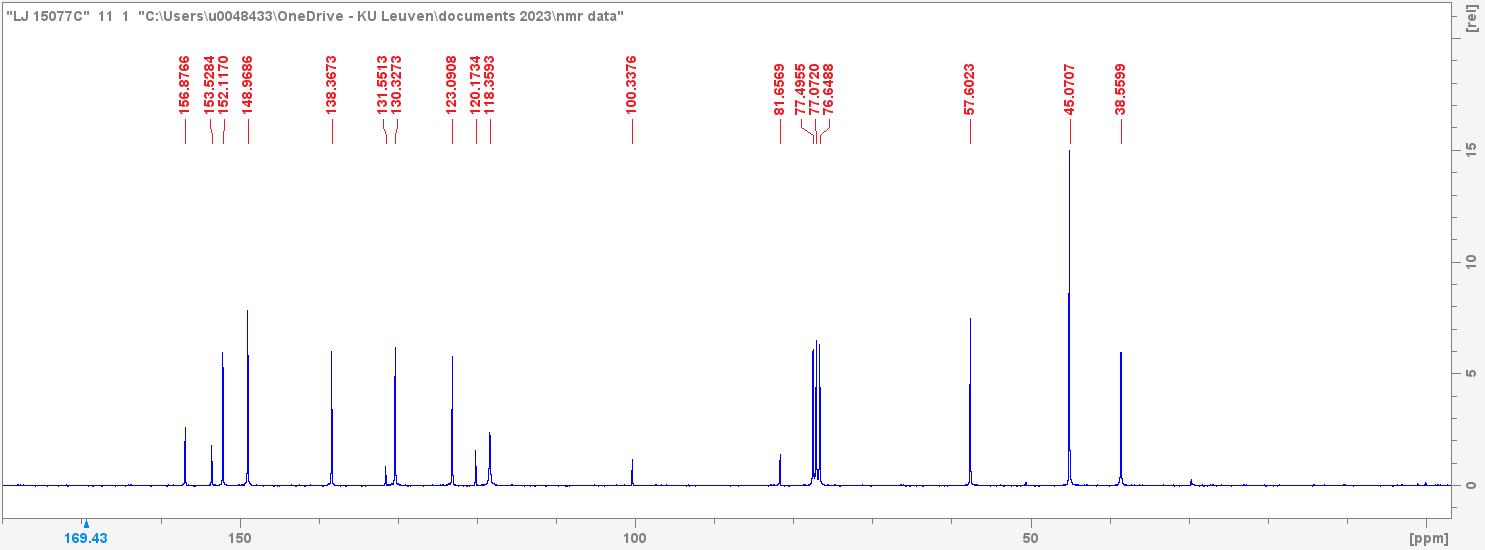


^1^H NMR spectrum of **16h**


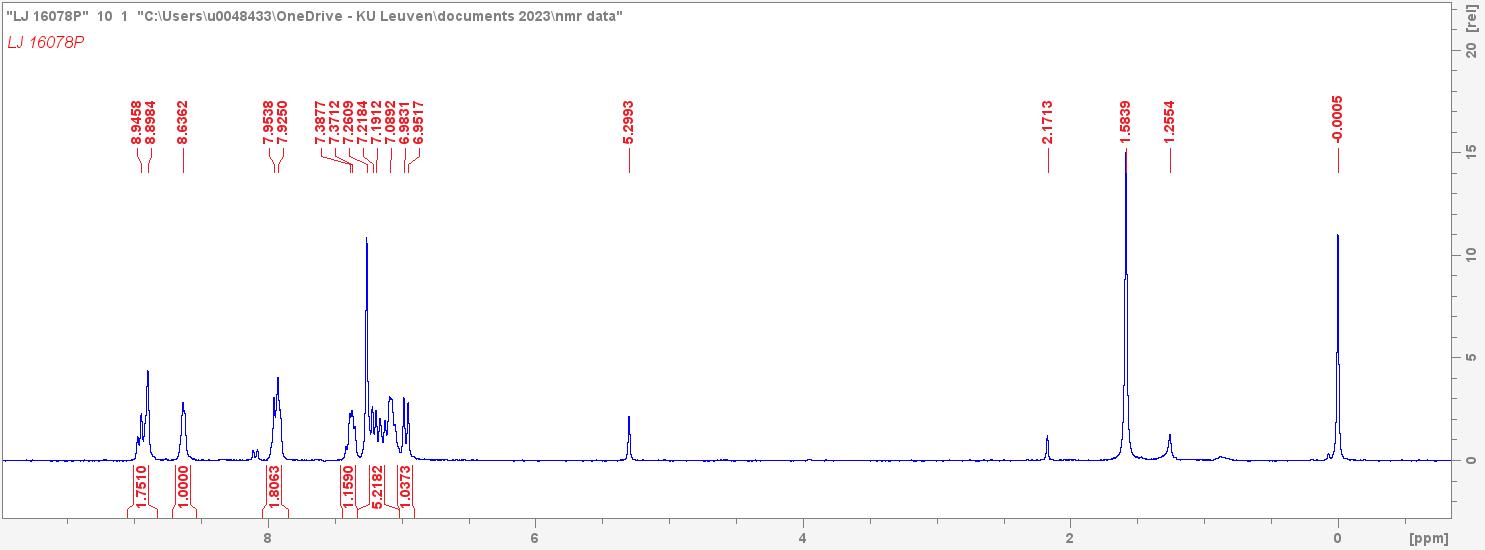


^13^C NMR spectrum of **16h**


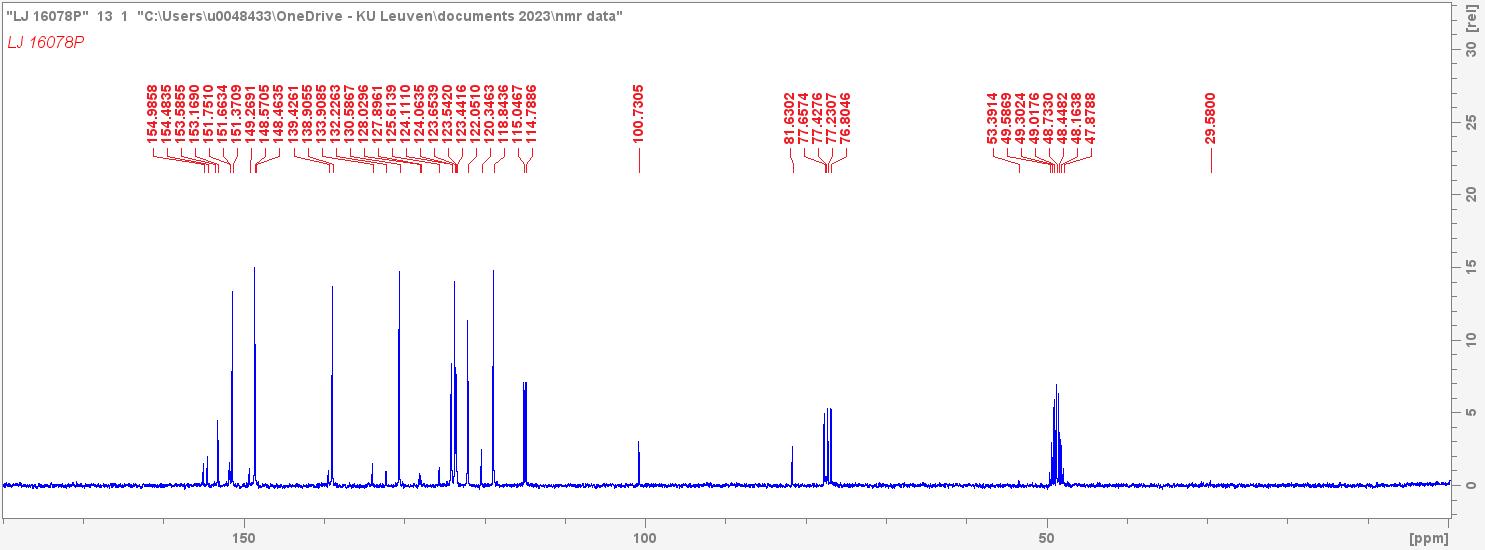


^1^H NMR spectrum of **16i**


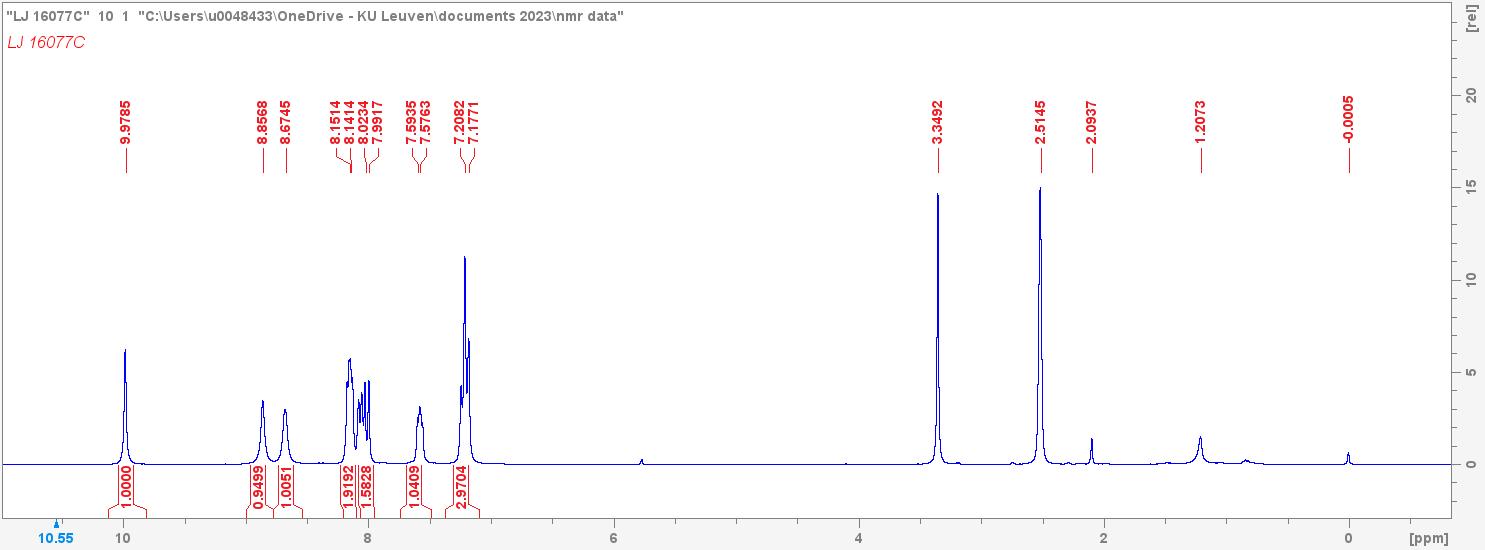


^13^C NMR spectrum of **16i**


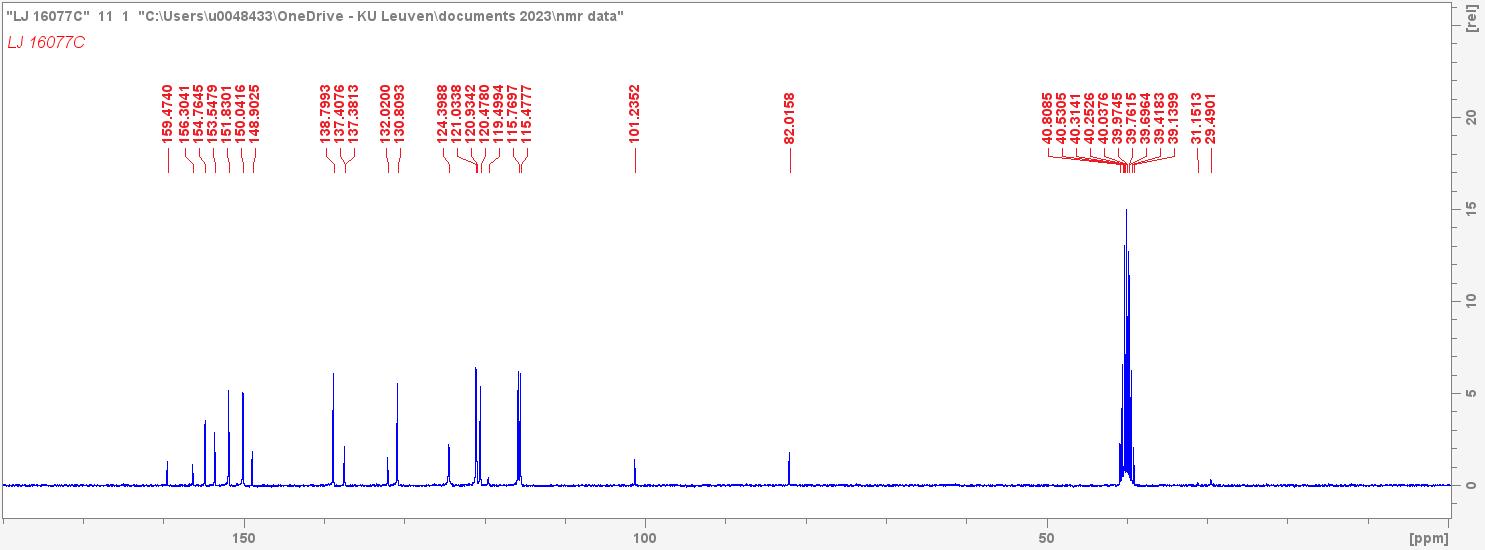


^1^H NMR spectrum of **16j**


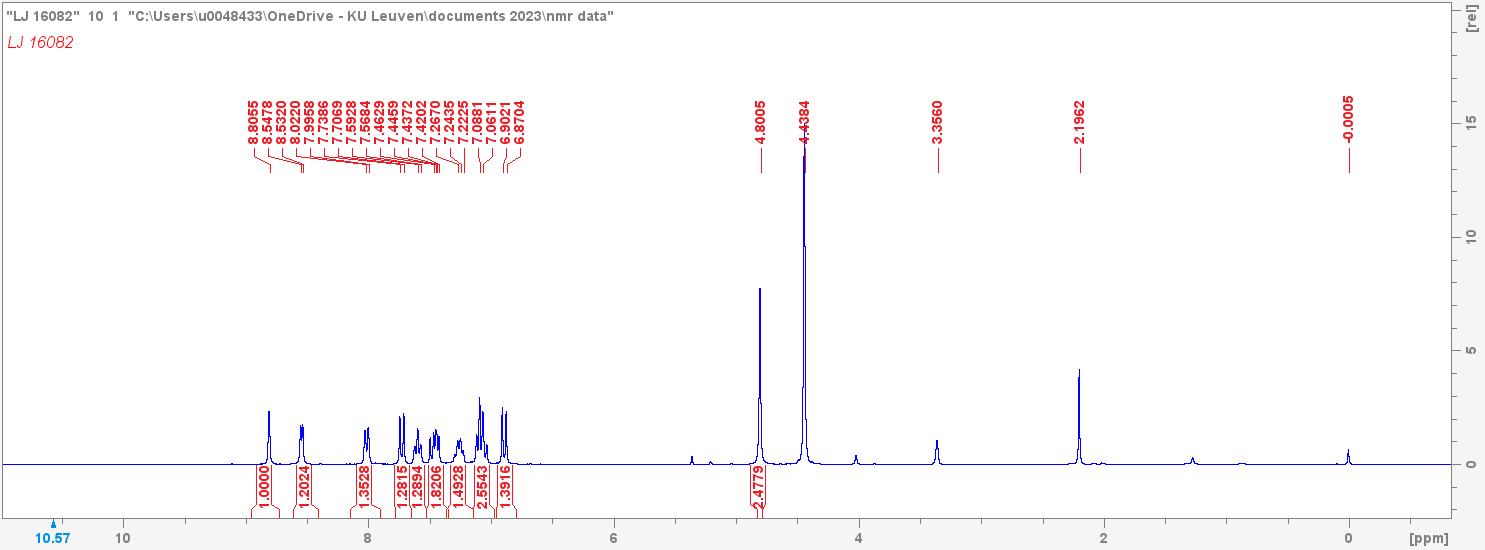


^13^C NMR spectrum of **16j**


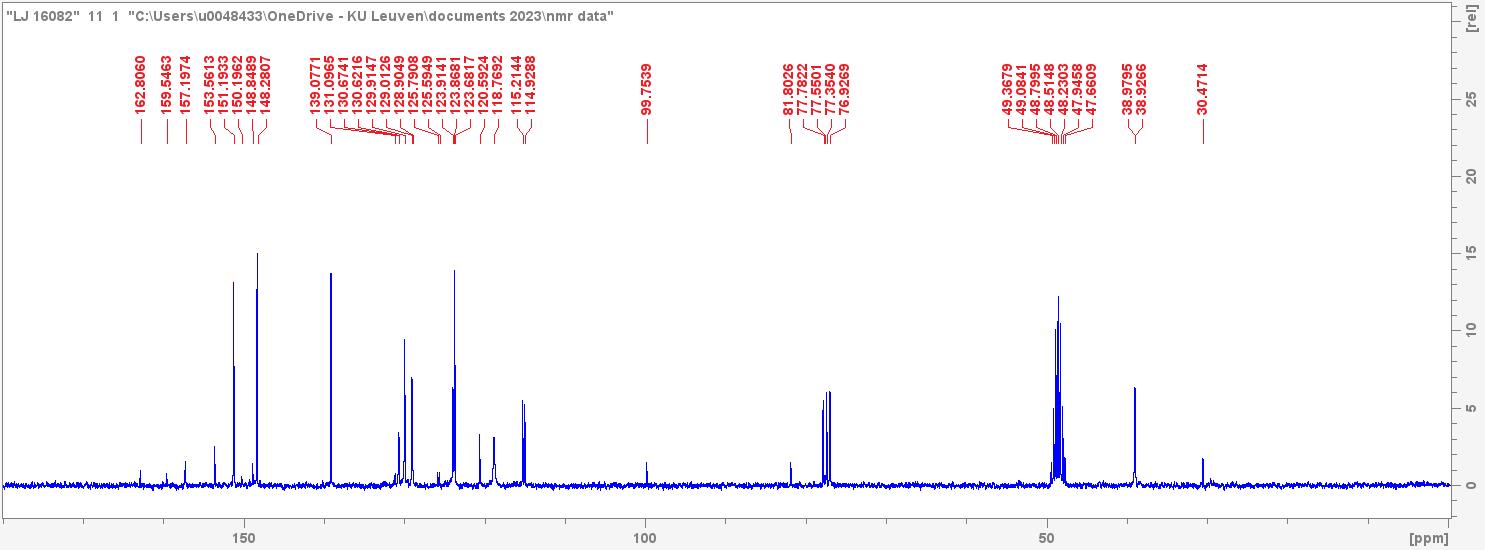


^1^H NMR spectrum of **16k**


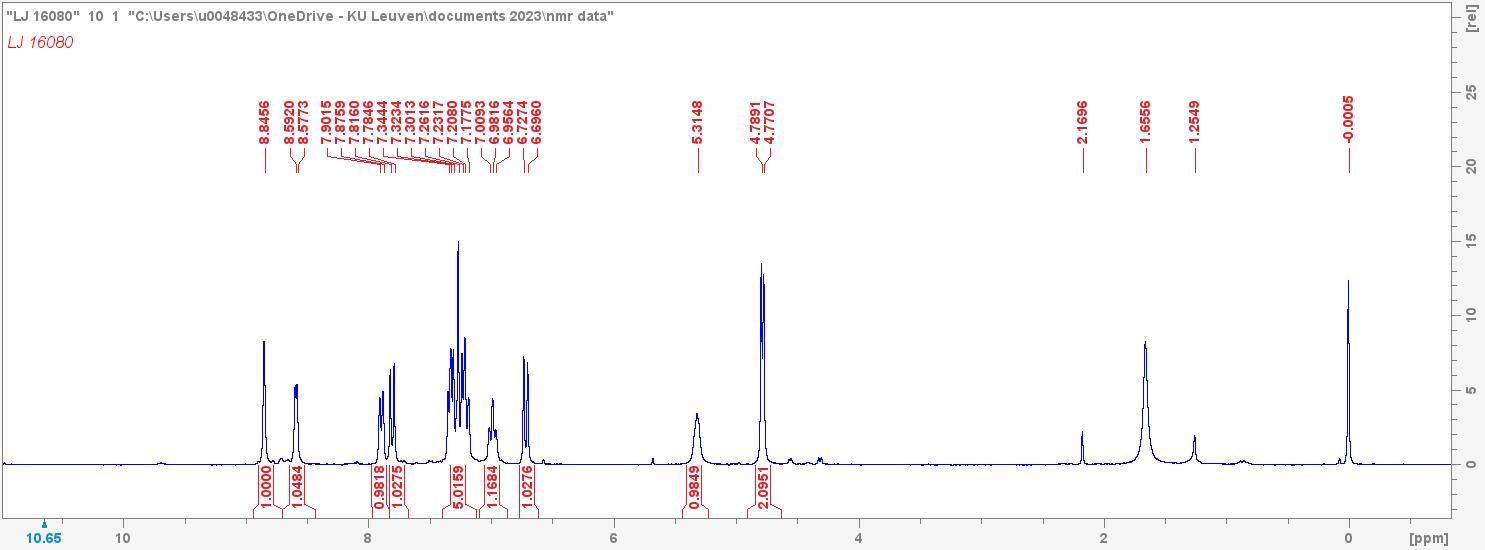


^13^C NMR spectrum of **16k**


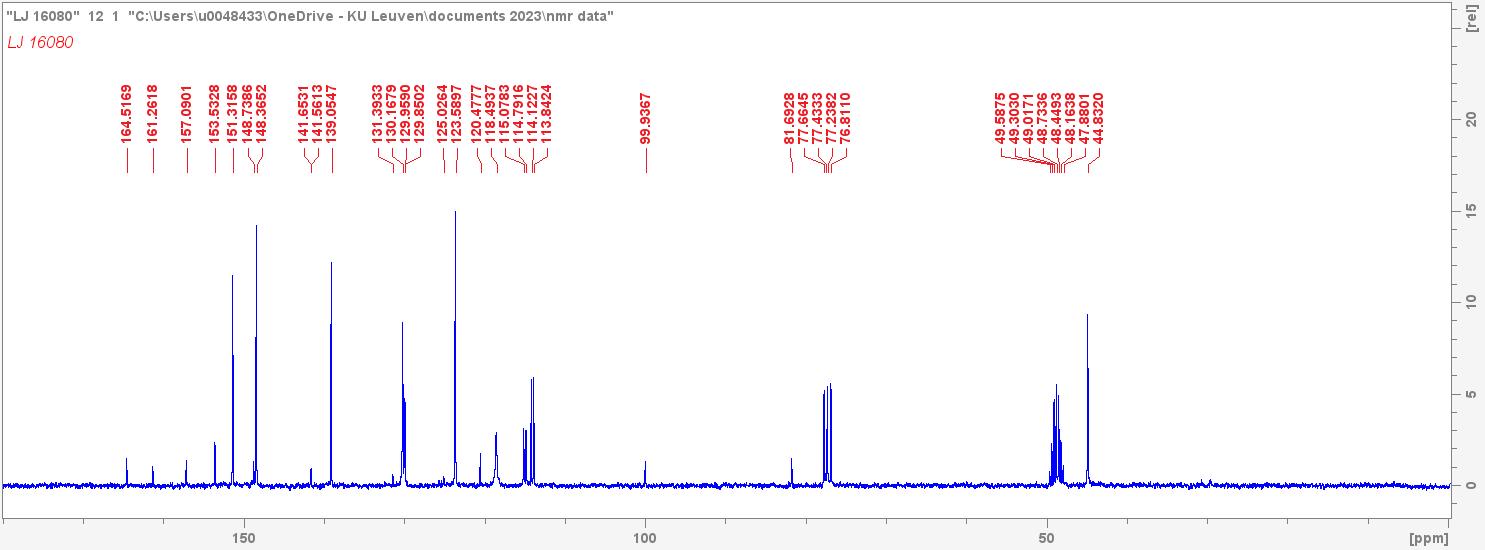


^1^H NMR spectrum of **16l**


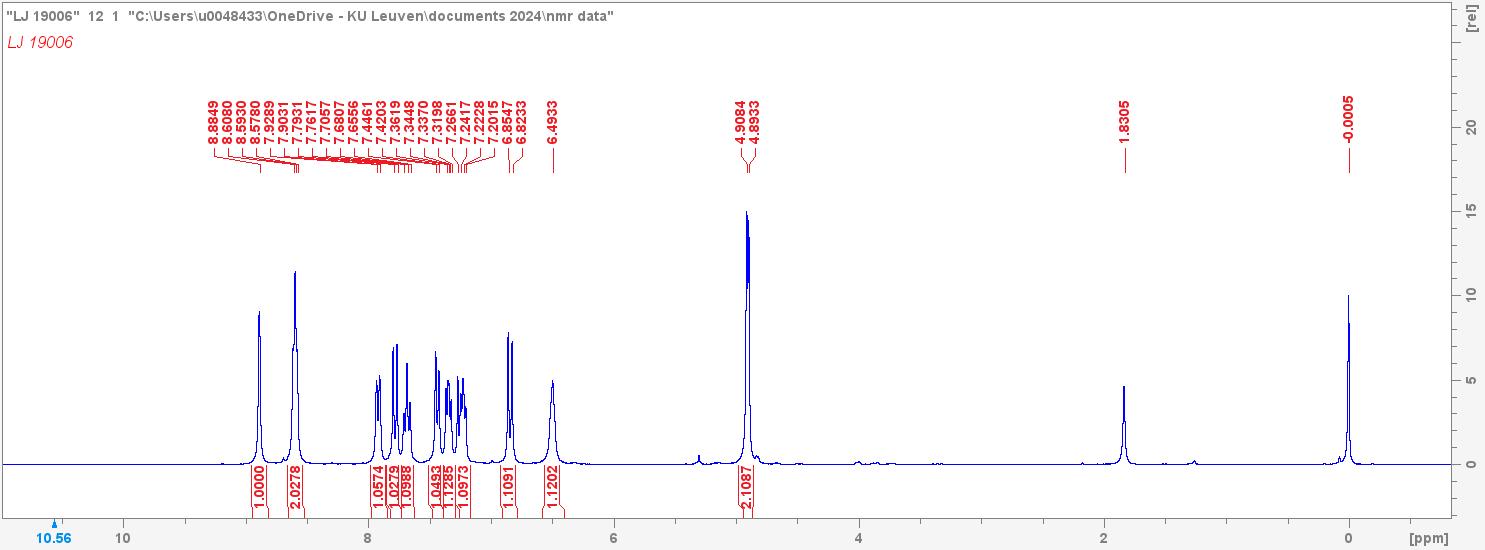


^13^C NMR spectrum of **16l**


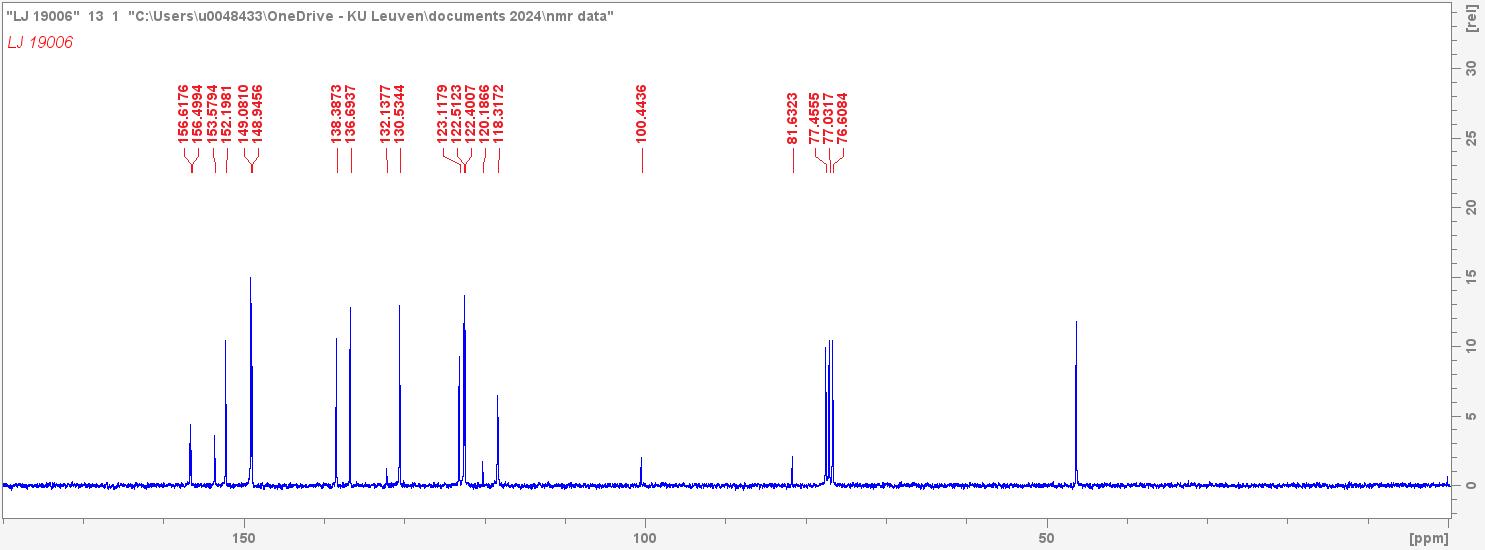


^1^H NMR spectrum of **16m**


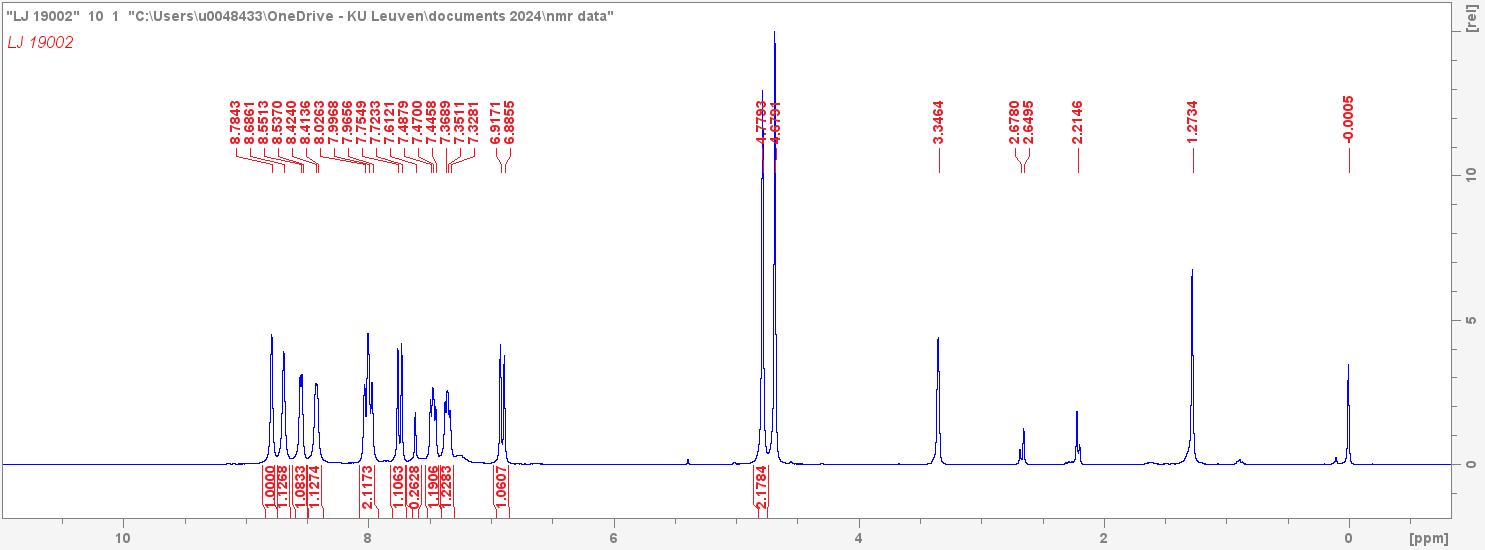


^13^C NMR spectrum of **16m**


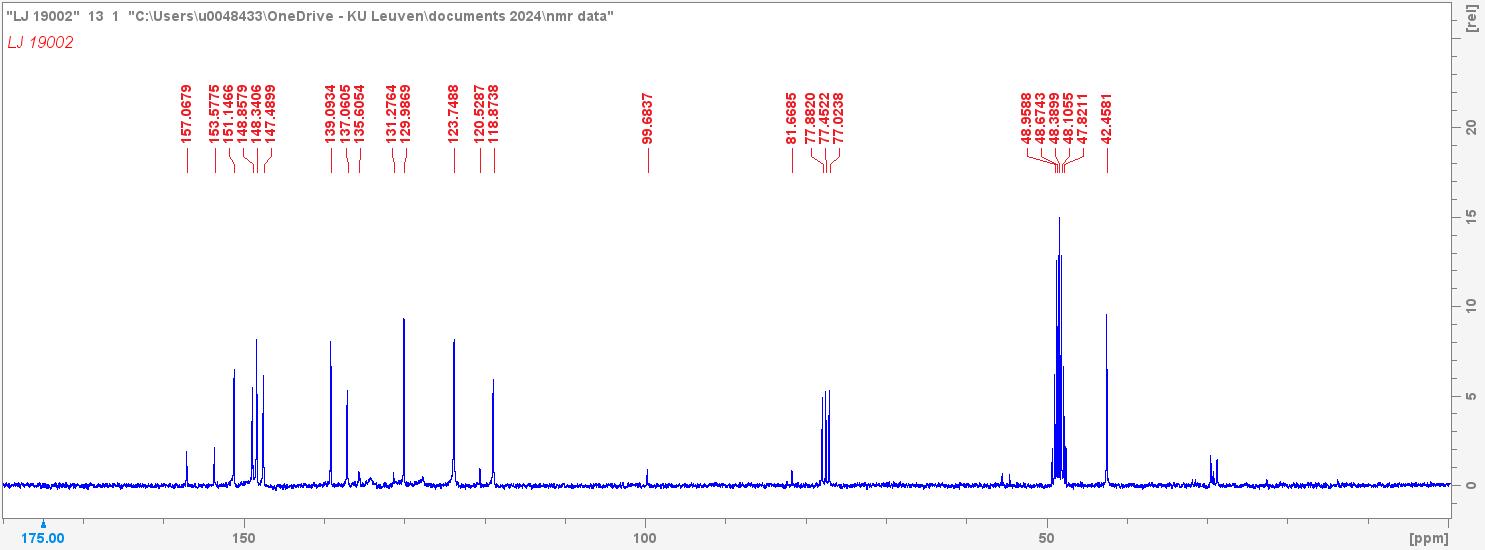


^1^H NMR spectrum of **16n**


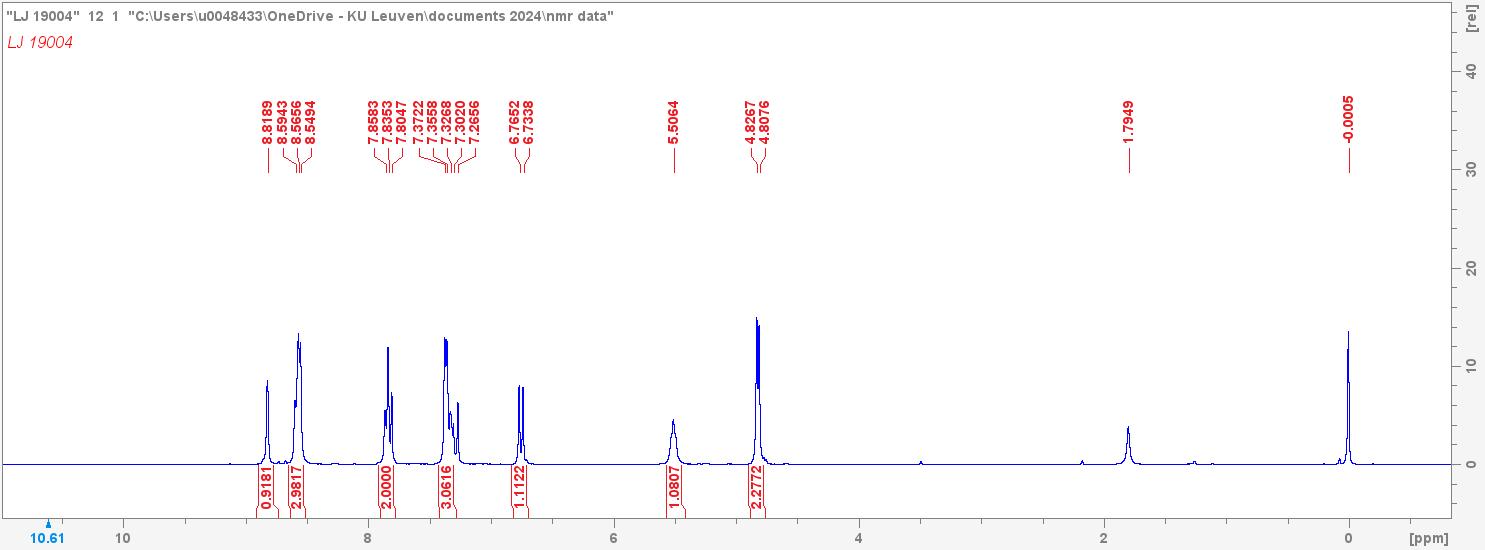


^13^C NMR spectrum of **16n**


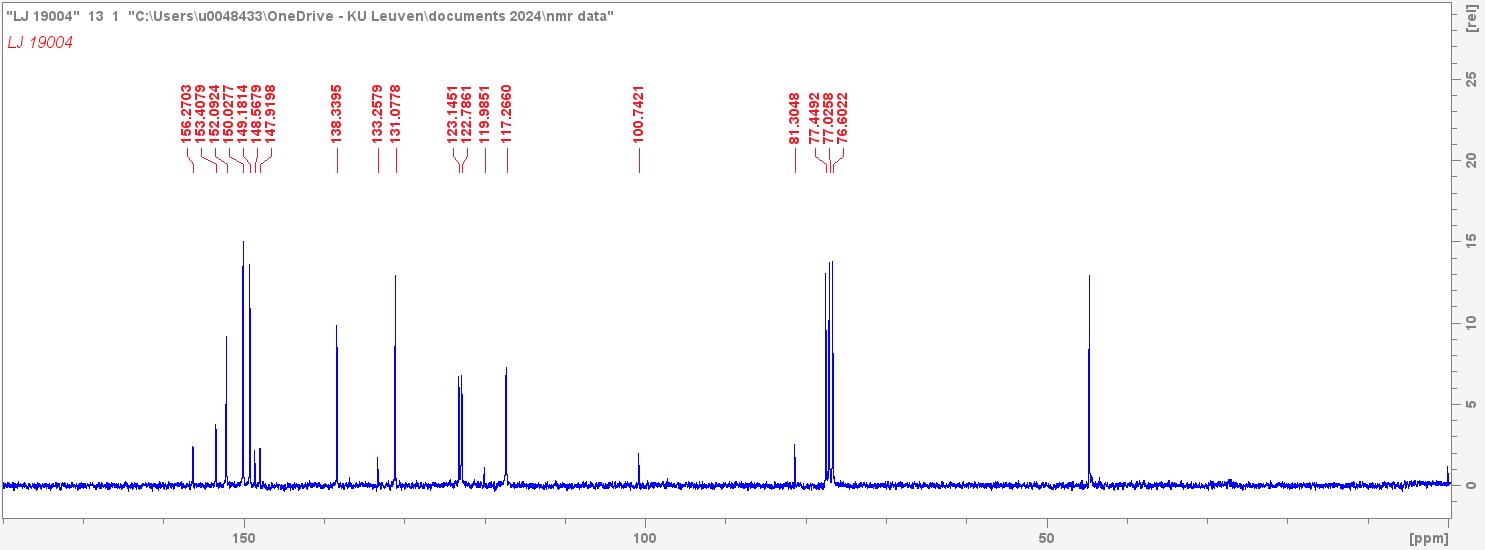


^1^H NMR spectrum of **16o**


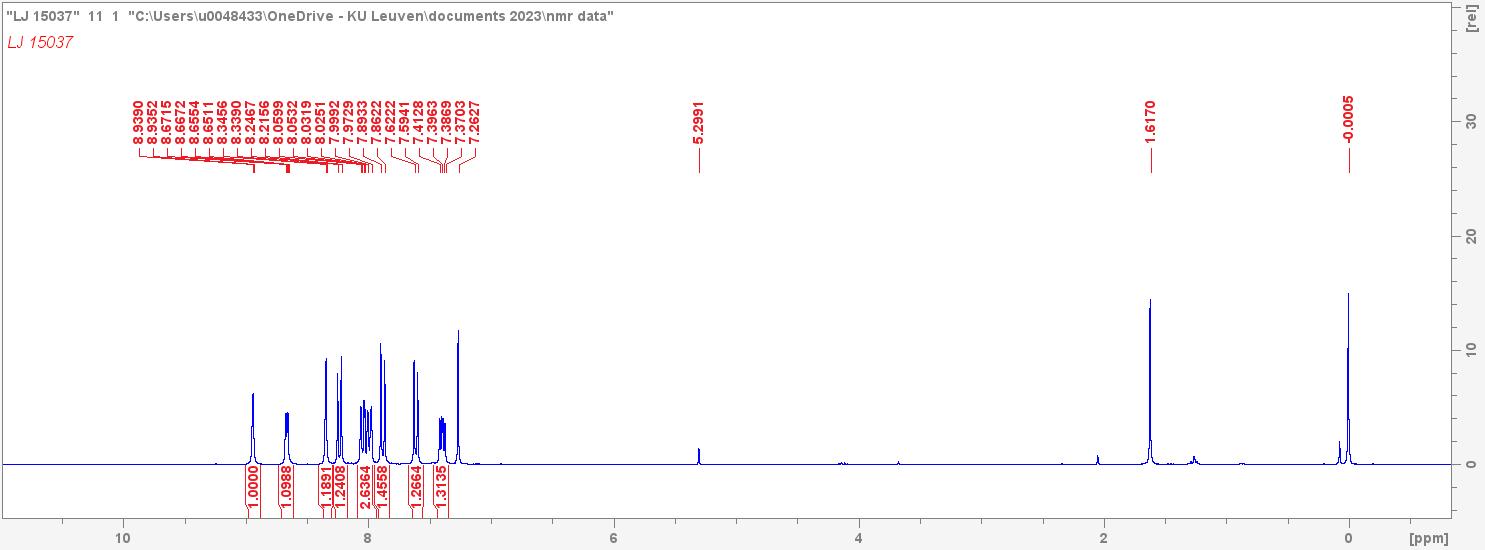


^13^C NMR spectrum of **16o**


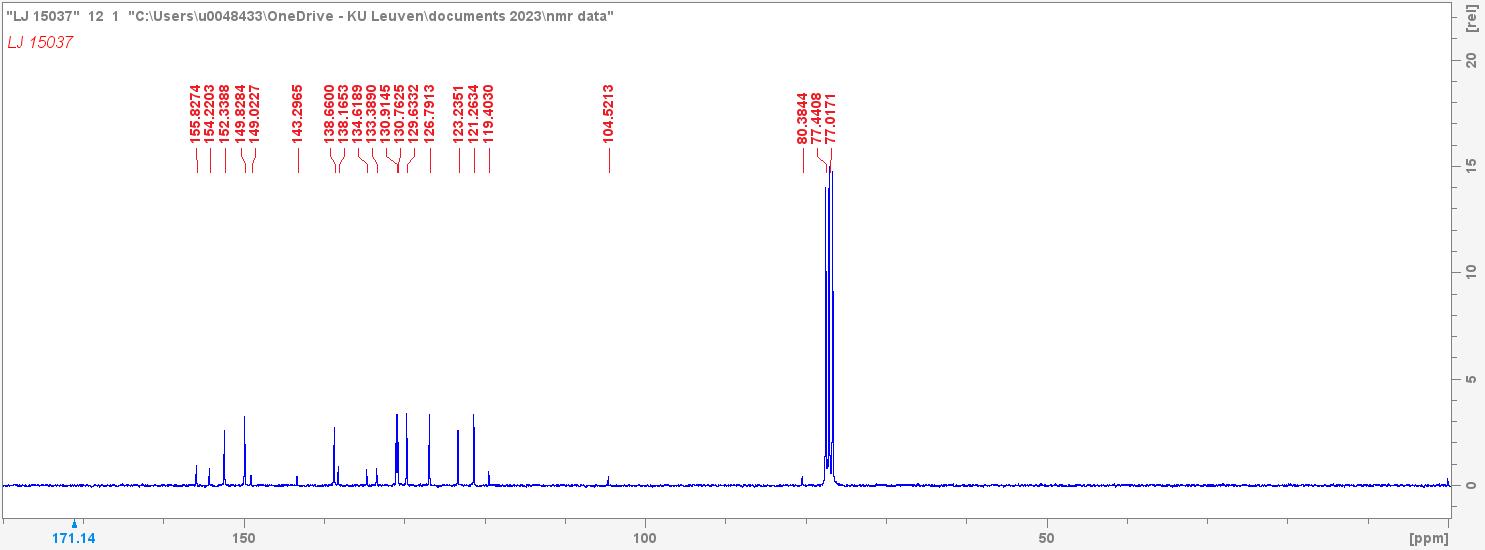


^1^H NMR spectrum of **16p**


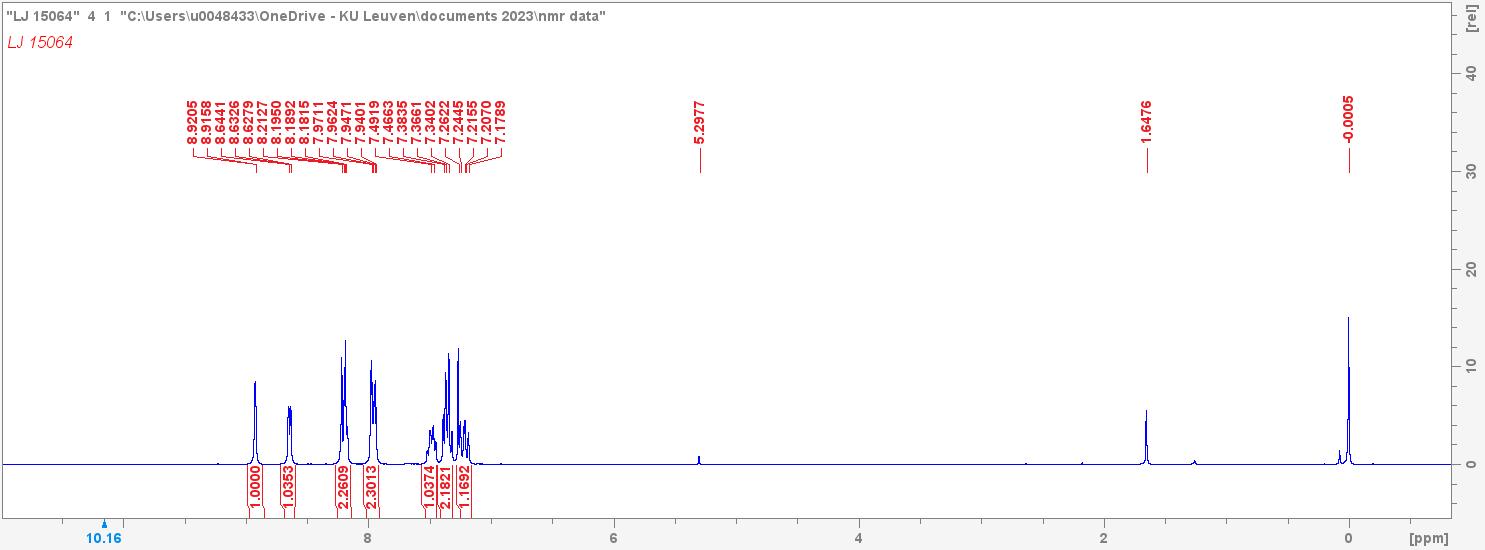


^13^C NMR spectrum of **16p**


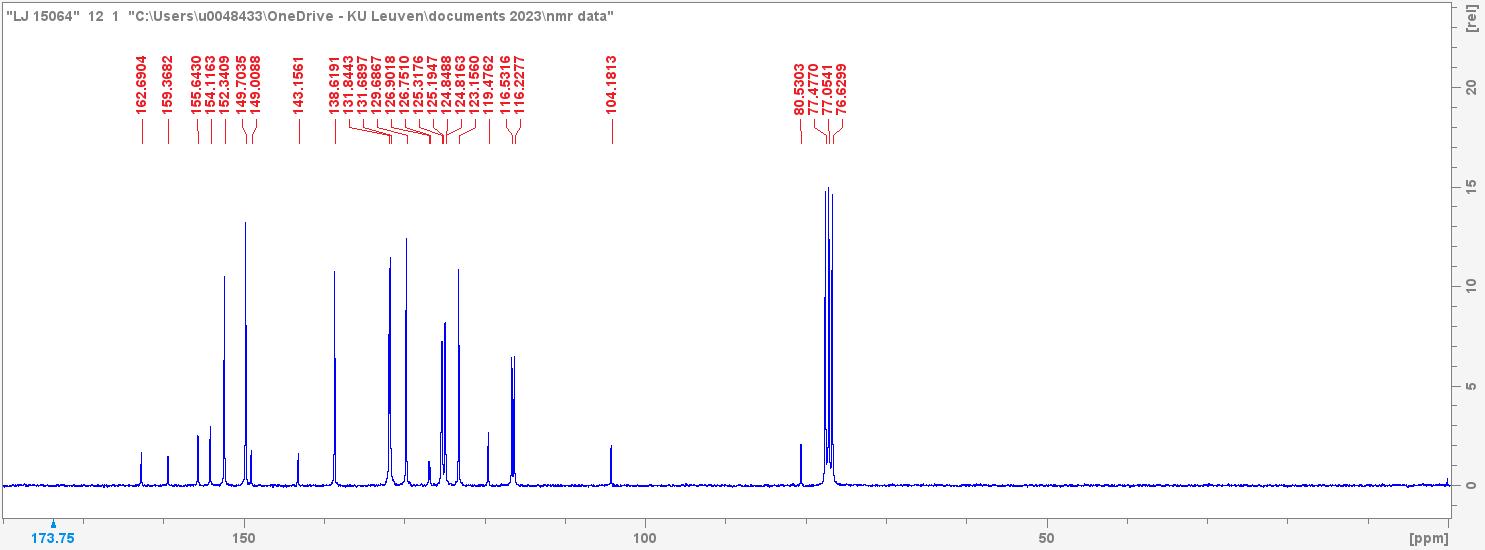


^1^H NMR spectrum of **17a**


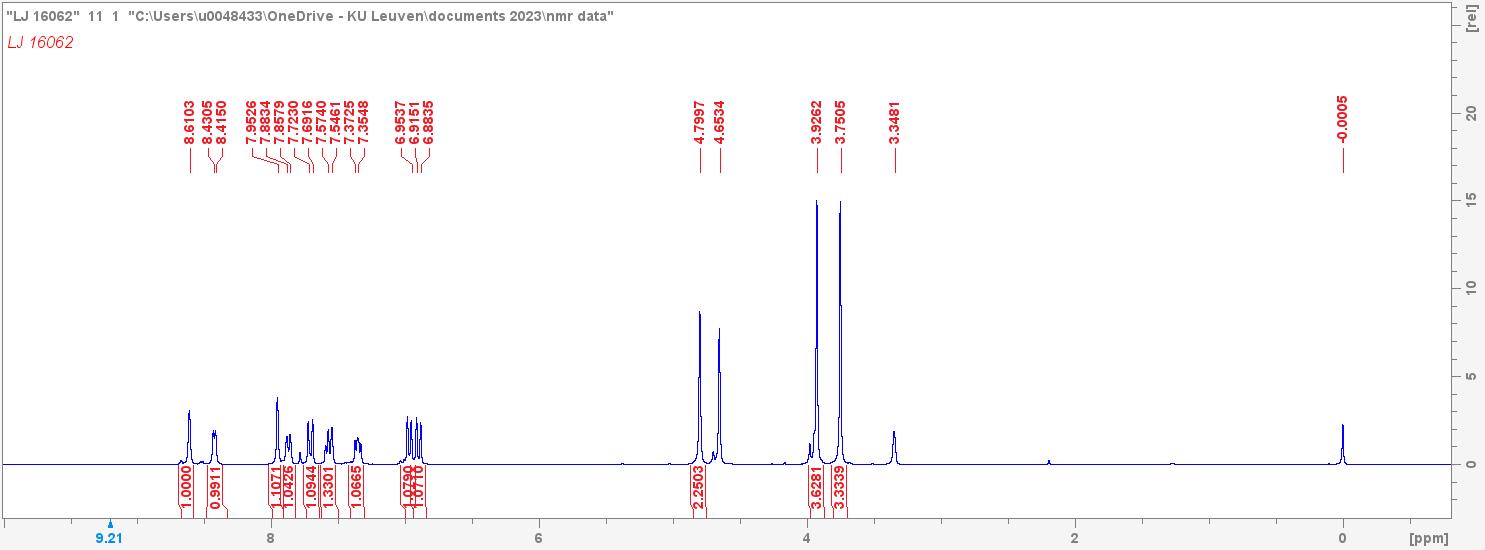


^13^C NMR spectrum of **17a**


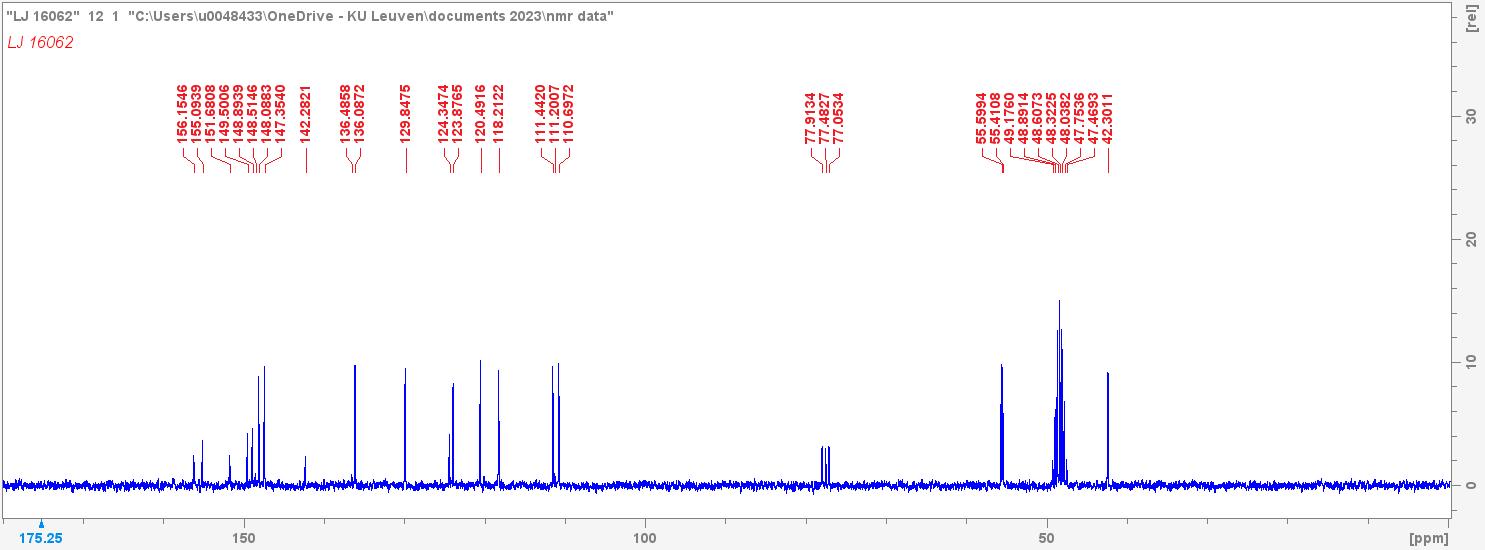


^1^H NMR spectrum of **17b**


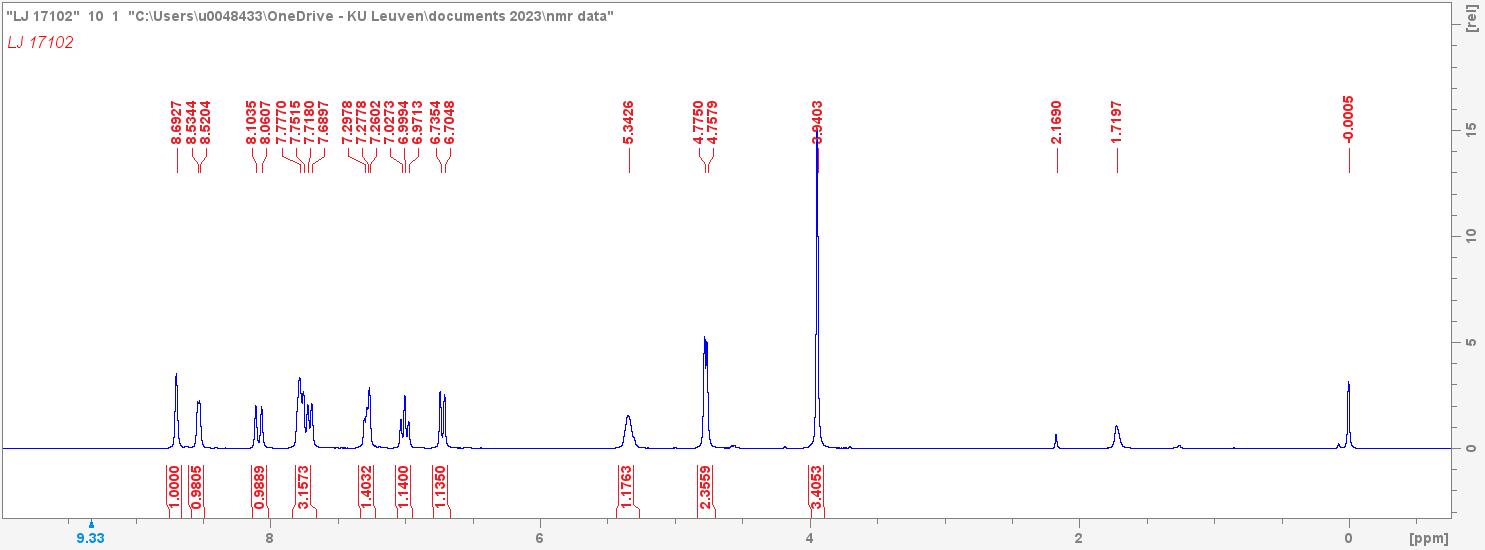


^13^C NMR spectrum of **17b**


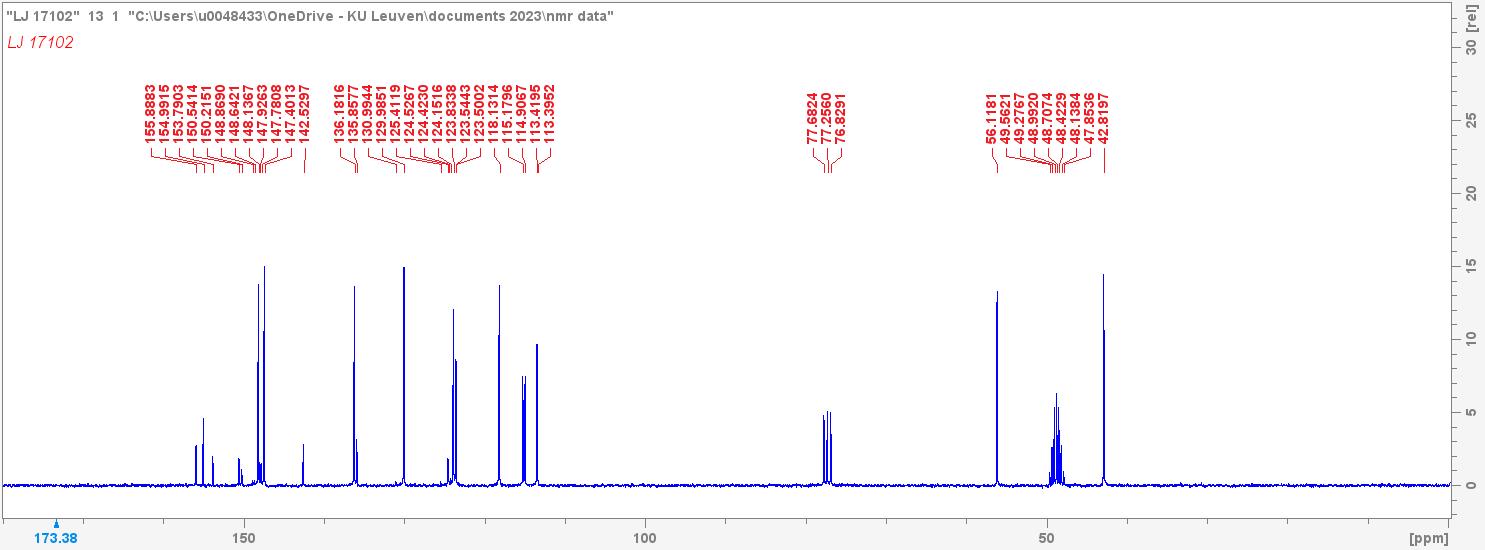


^1^H NMR spectrum of **17c**


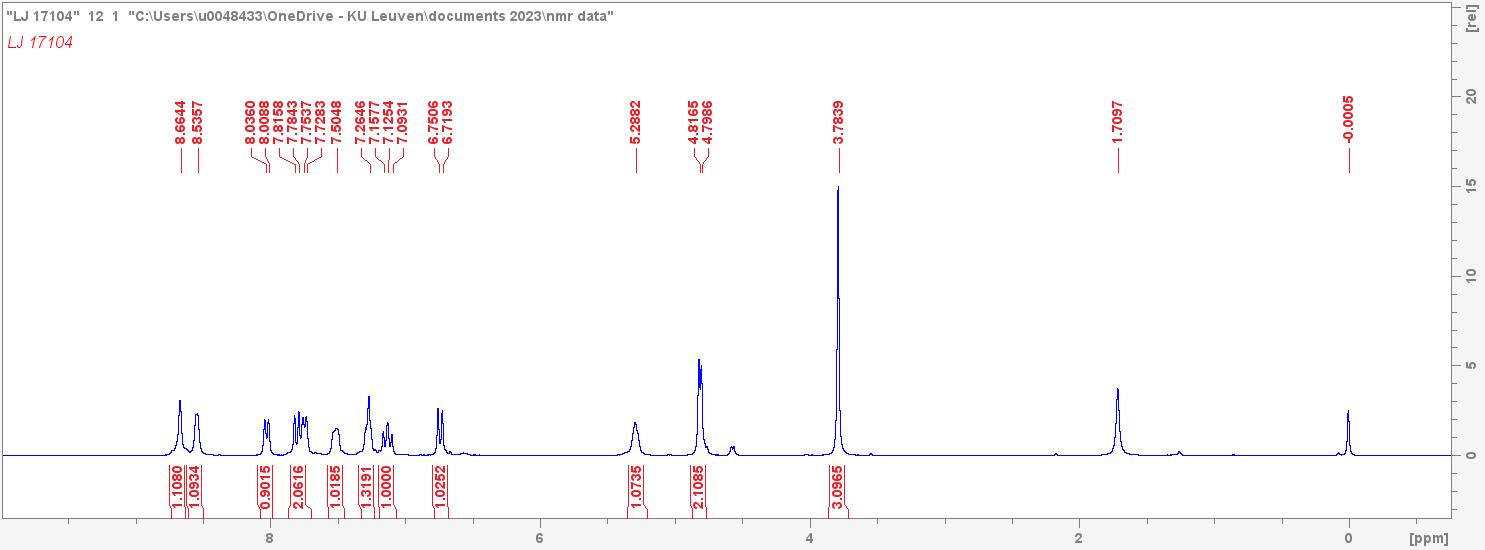


^13^C NMR spectrum of **17c**


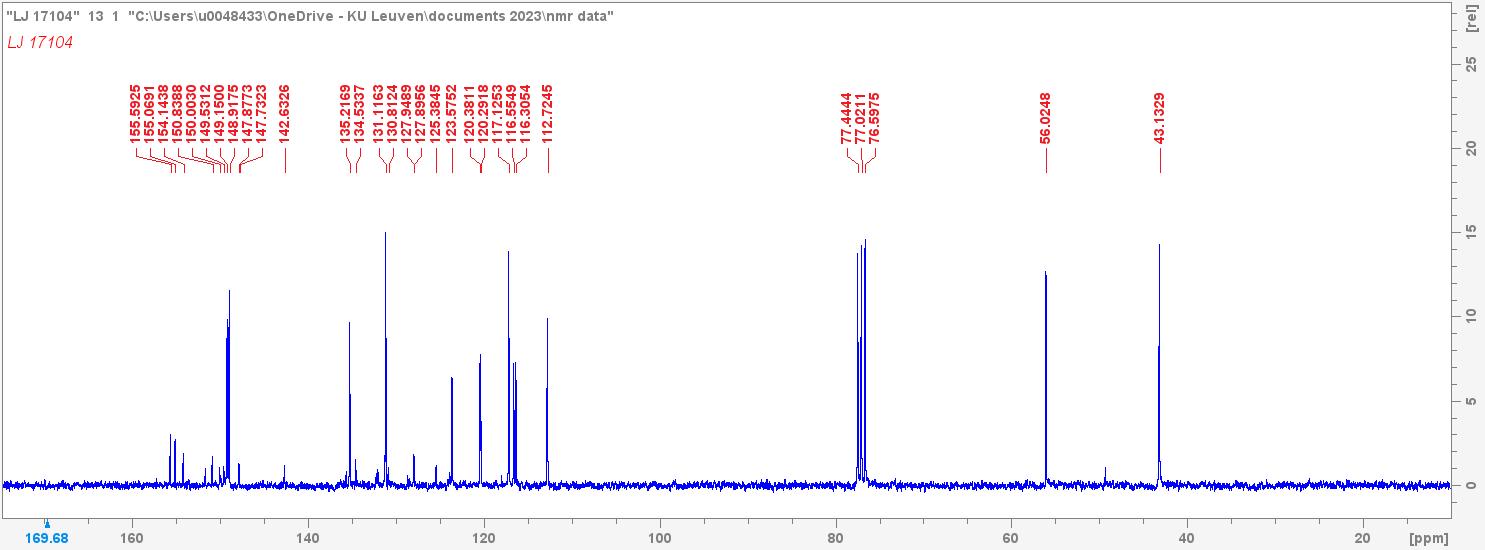


^1^H NMR spectrum of **17d**


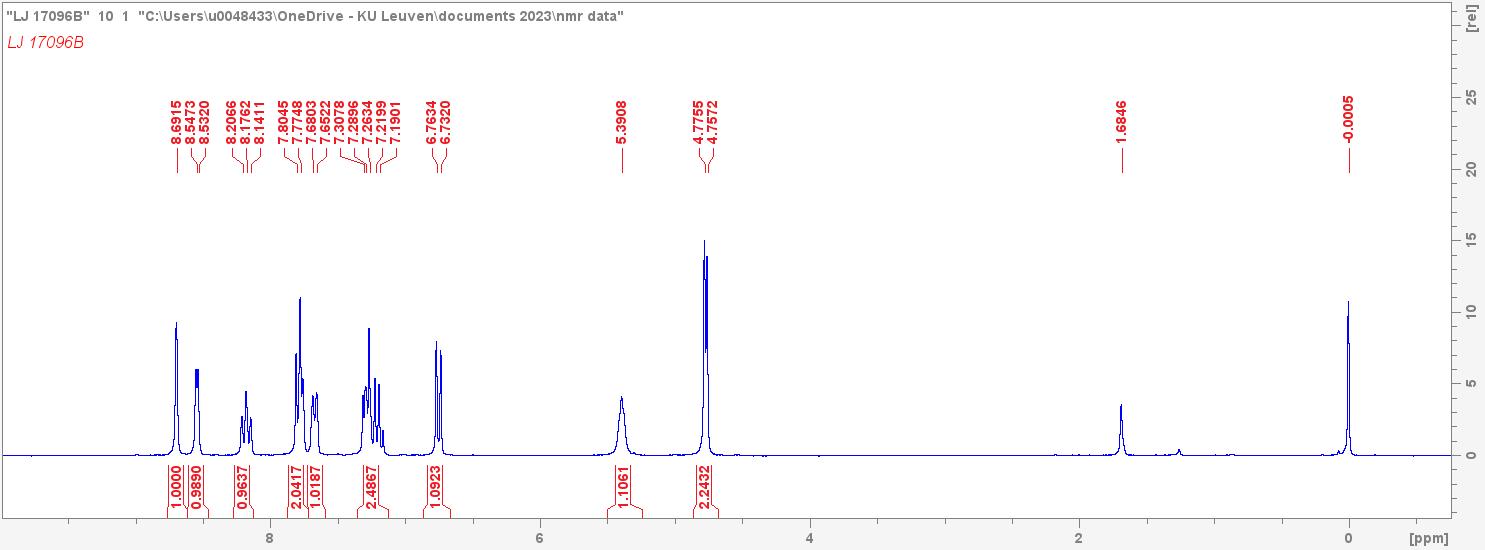


^13^C NMR spectrum of **17d**


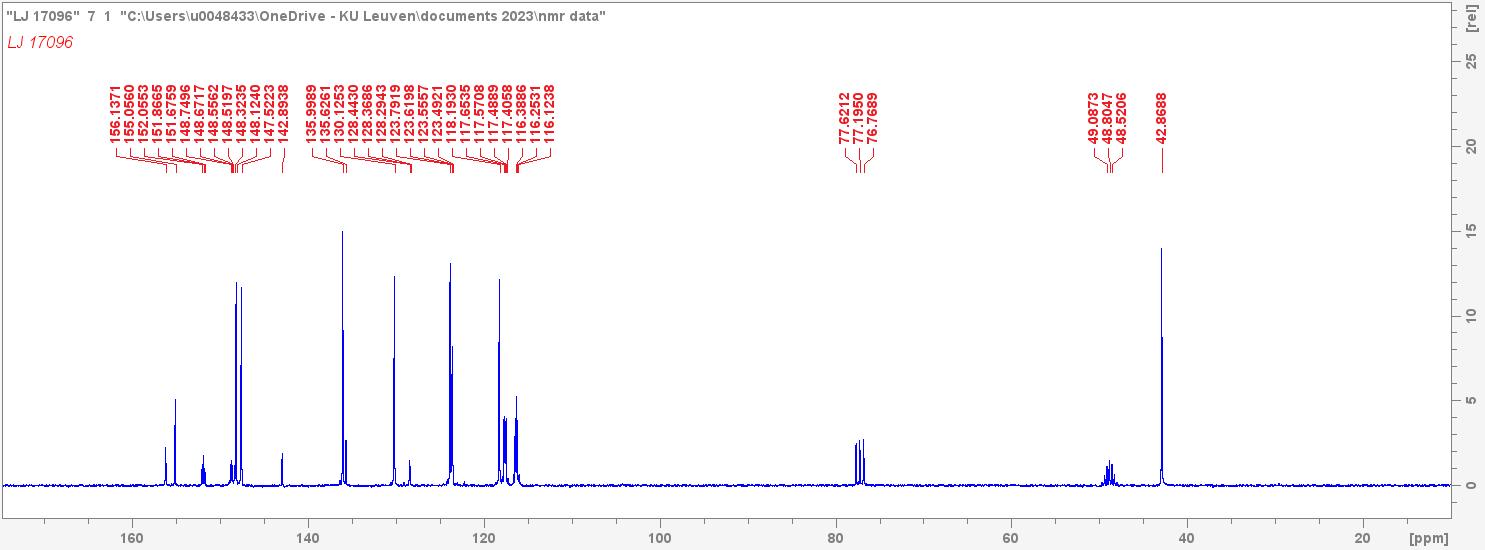


^1^H NMR spectrum of **17e**


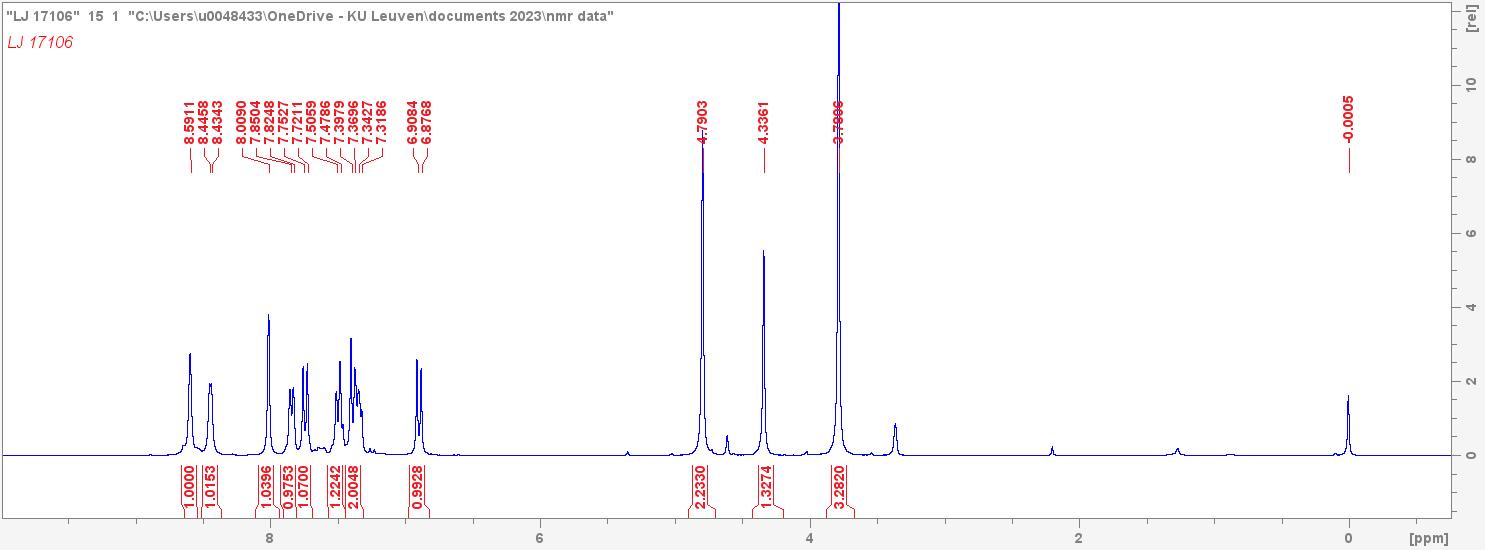


^13^C NMR spectrum of **17e**


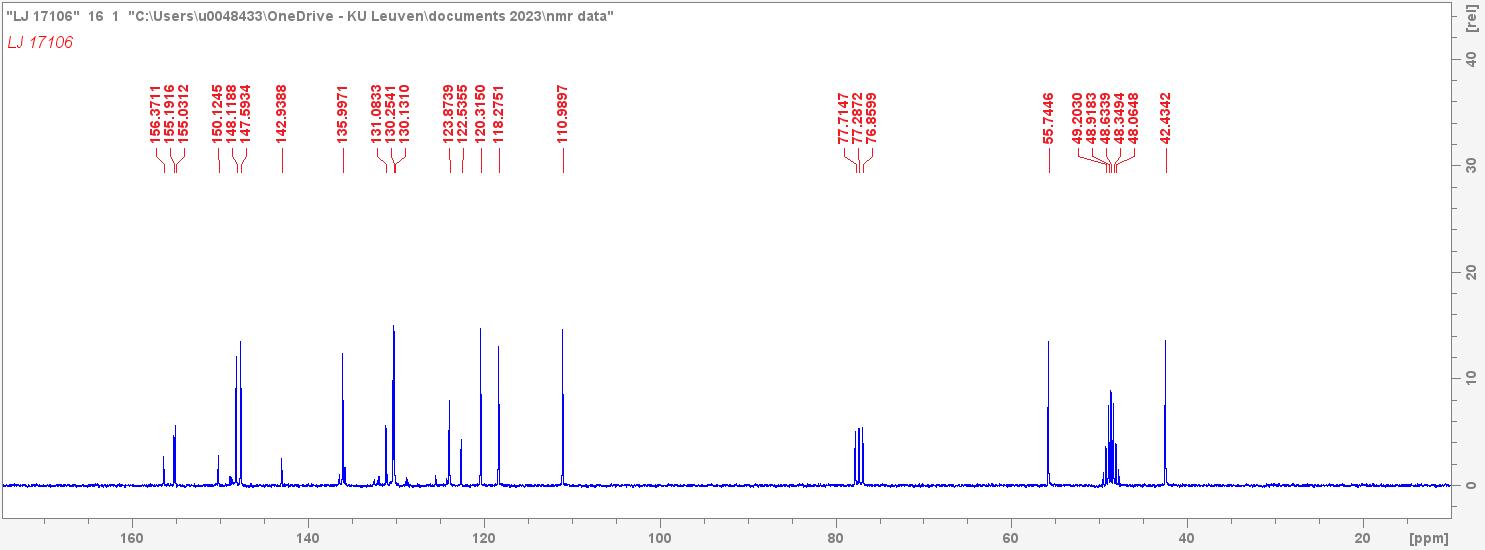


^1^H NMR spectrum of **17f**


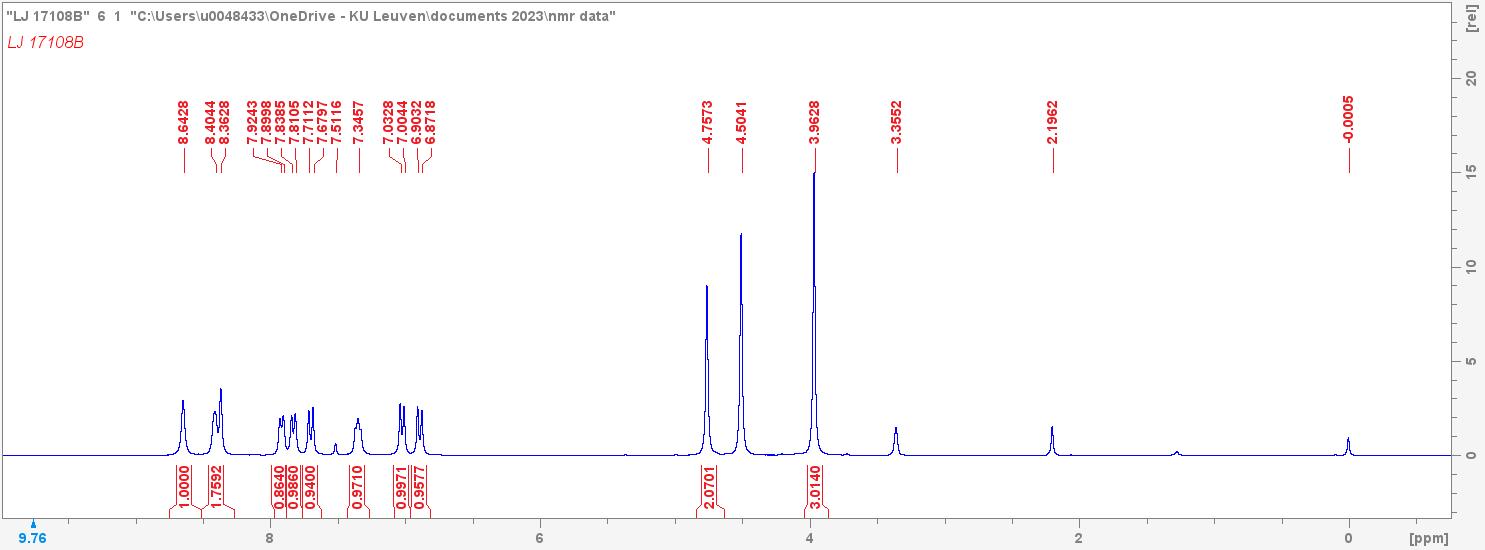


^13^C NMR spectrum of **17f**


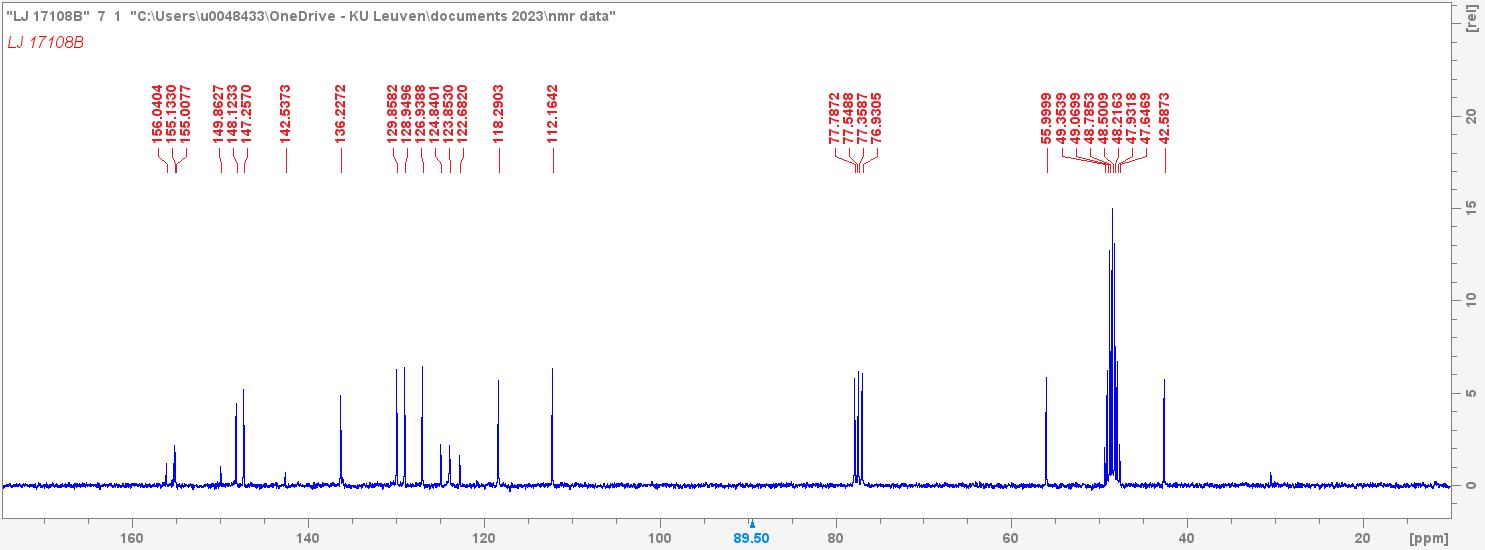


^1^H NMR spectrum of **17g**


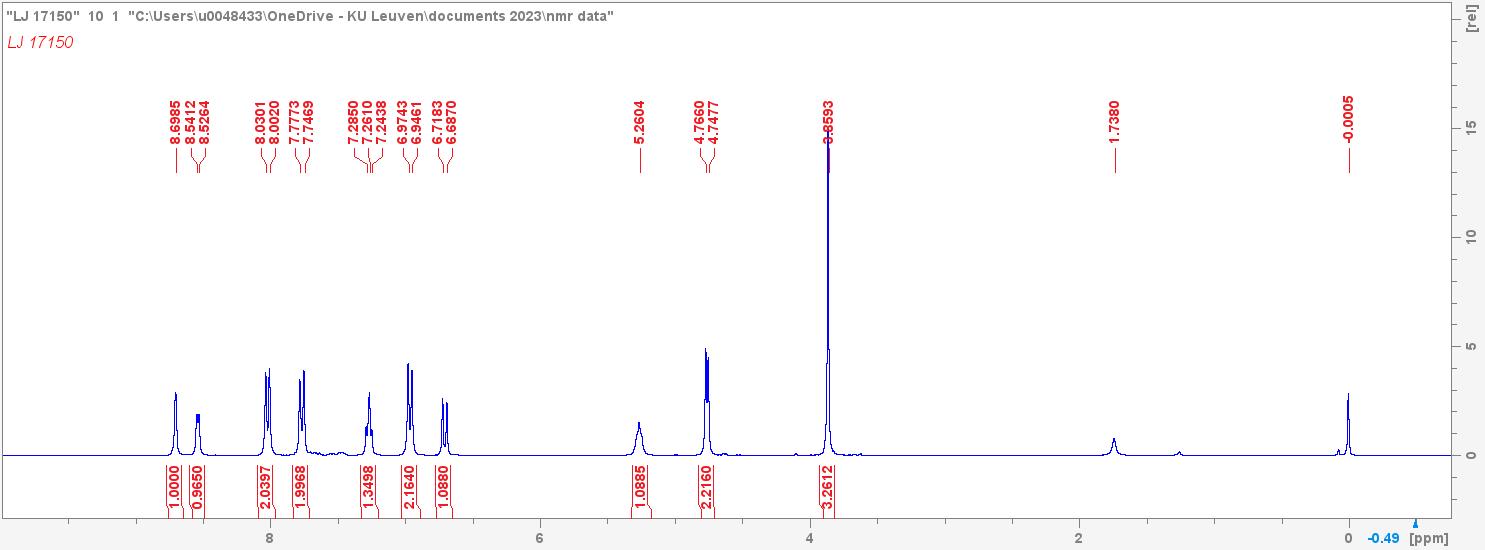


^13^C NMR spectrum of **17g**


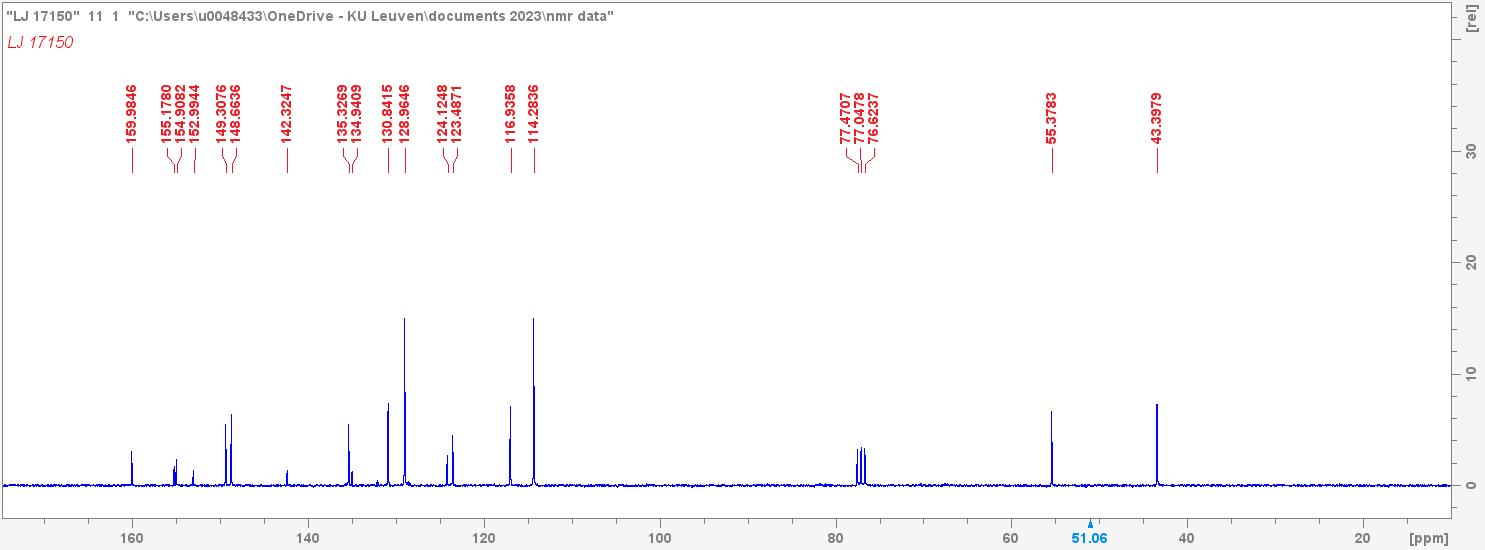


^1^H NMR spectrum of **17h**


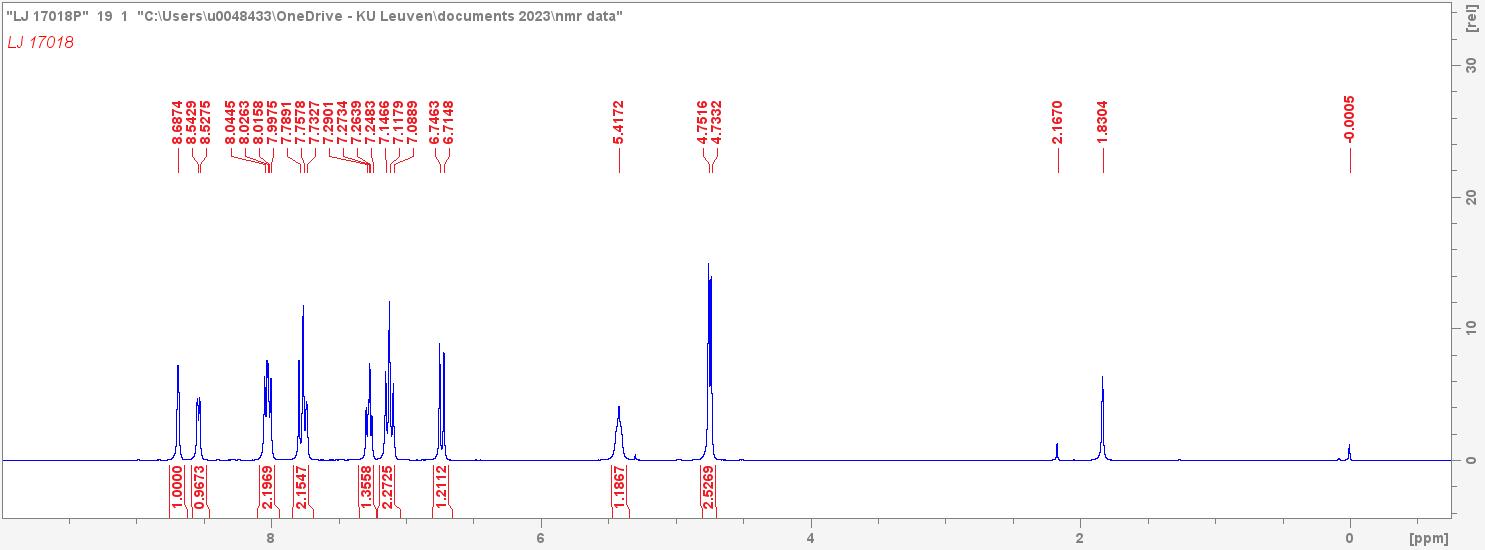


^13^C NMR spectrum of **17h**


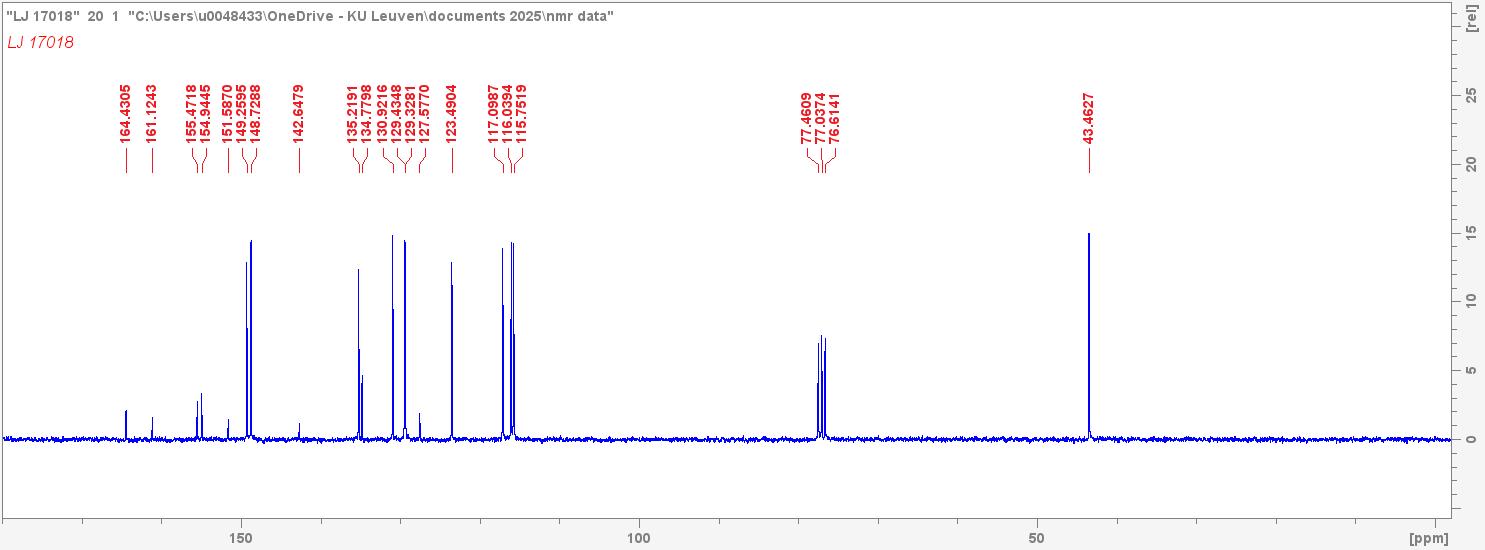


^1^H NMR spectrum of **17i**


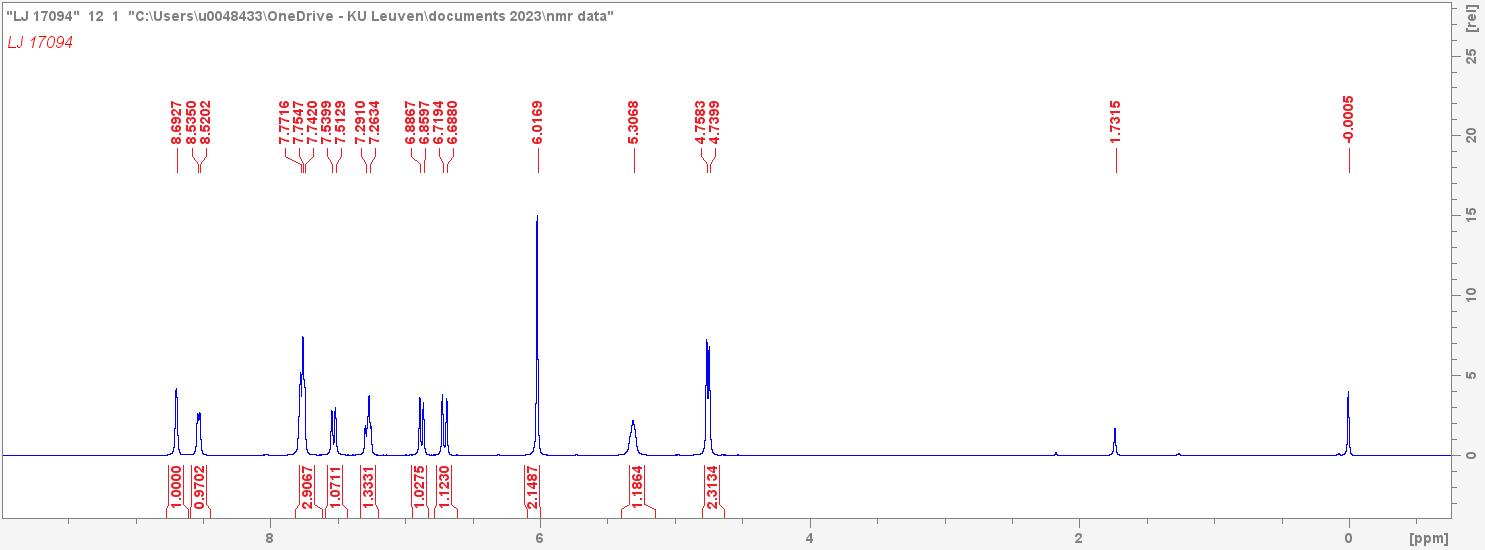


^13^C NMR spectrum of **17i**


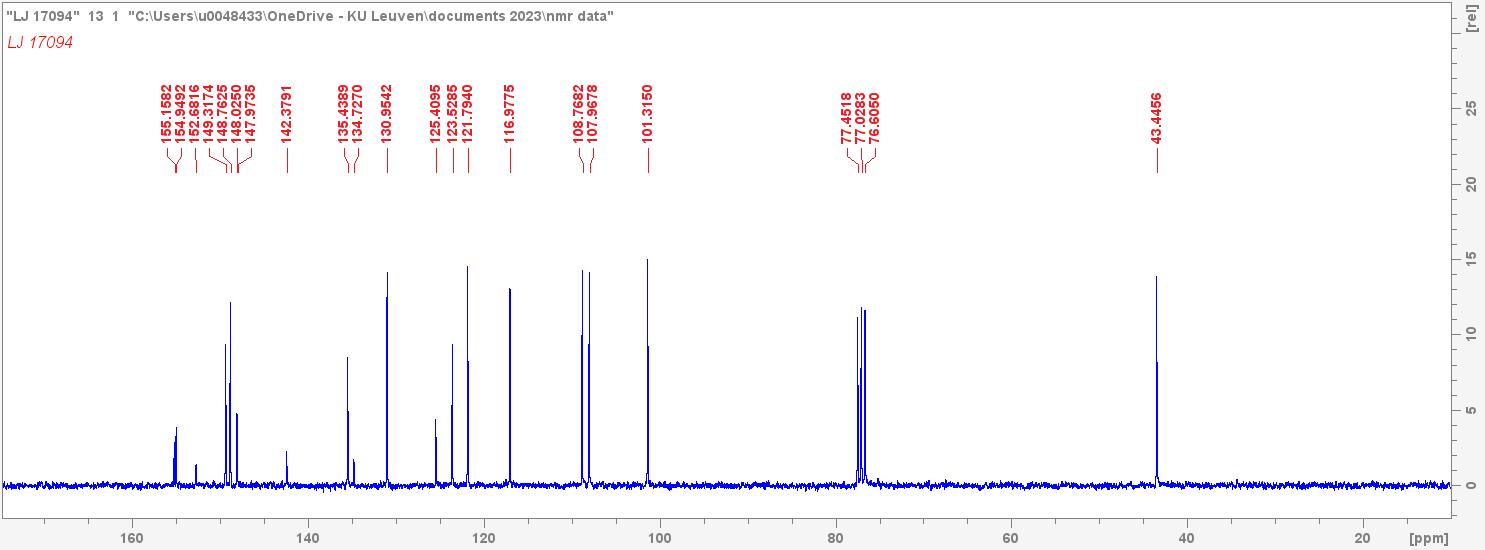


^1^H NMR spectrum of **17j**


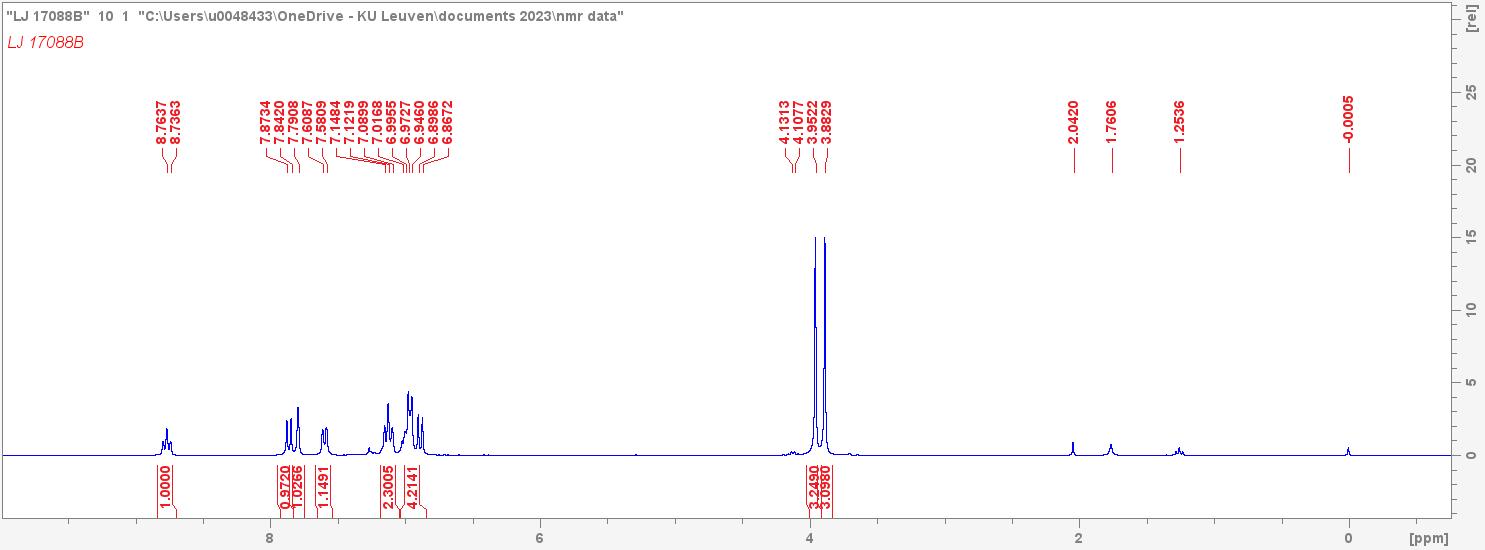


^13^C NMR spectrum of **17j**


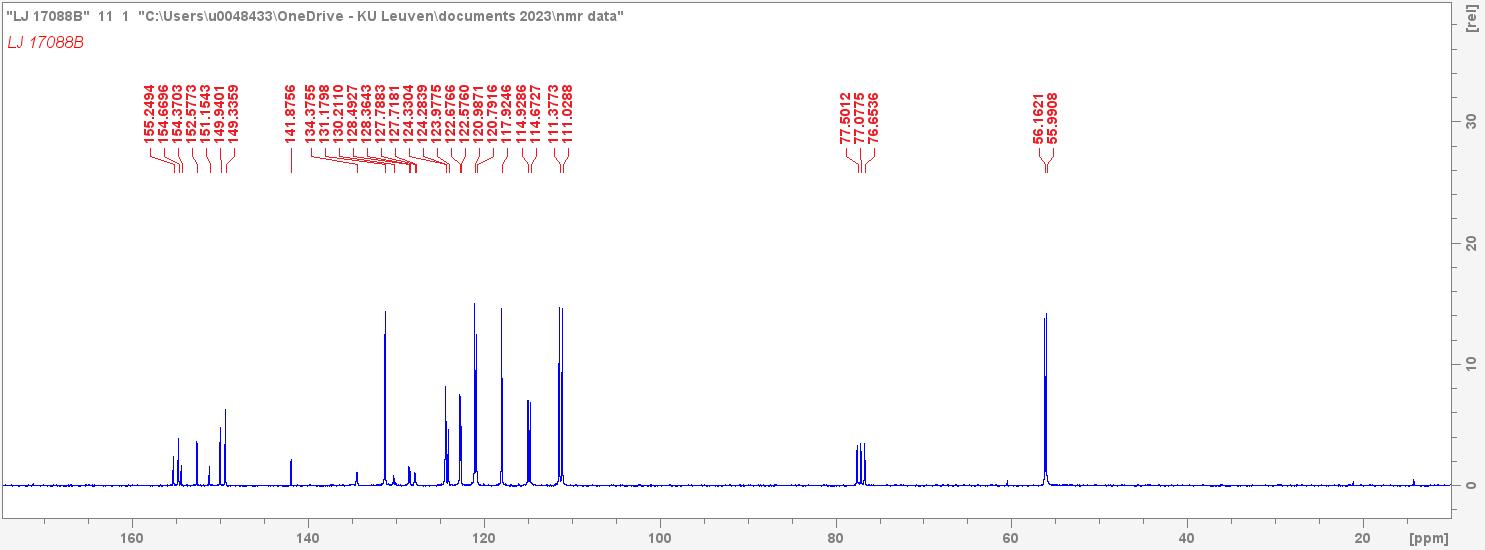


^1^H NMR spectrum of **17k**


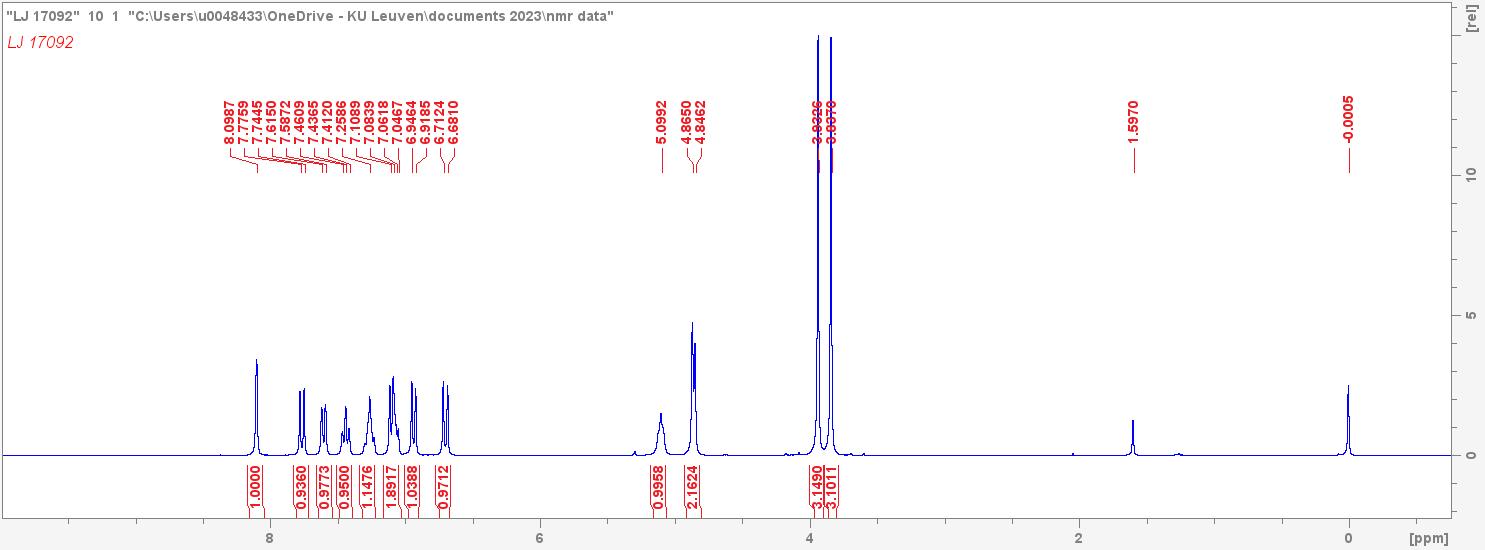


^13^C NMR spectrum of **17k**


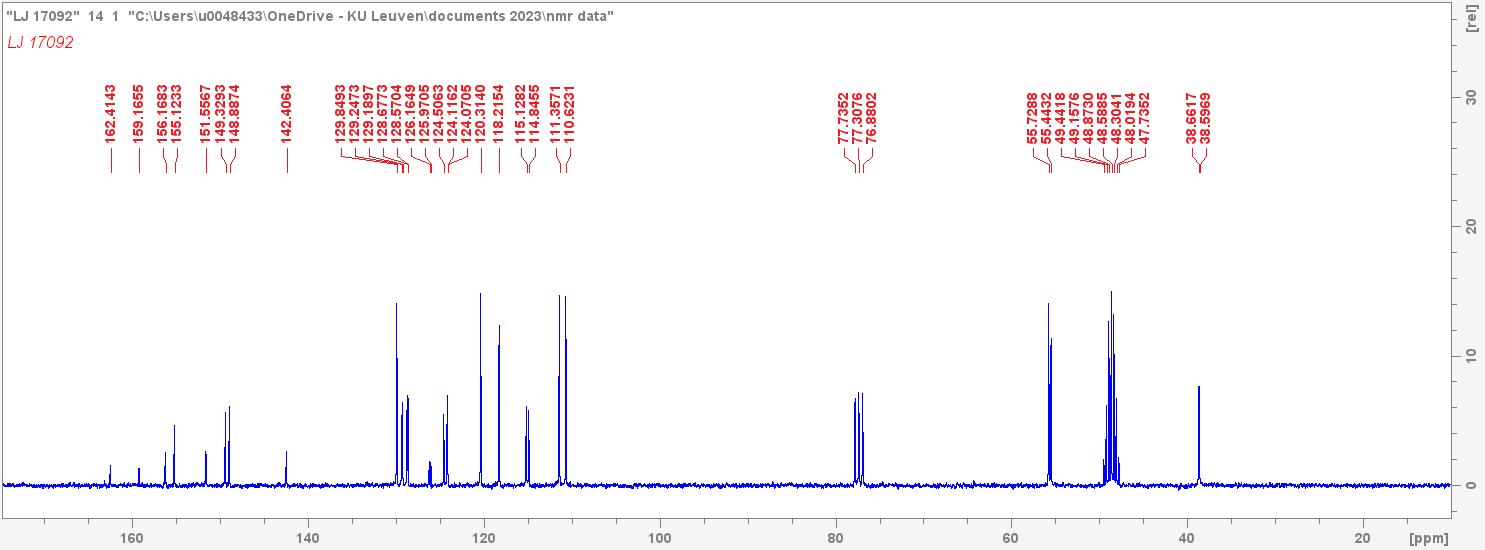


^1^H NMR spectrum of **17l**


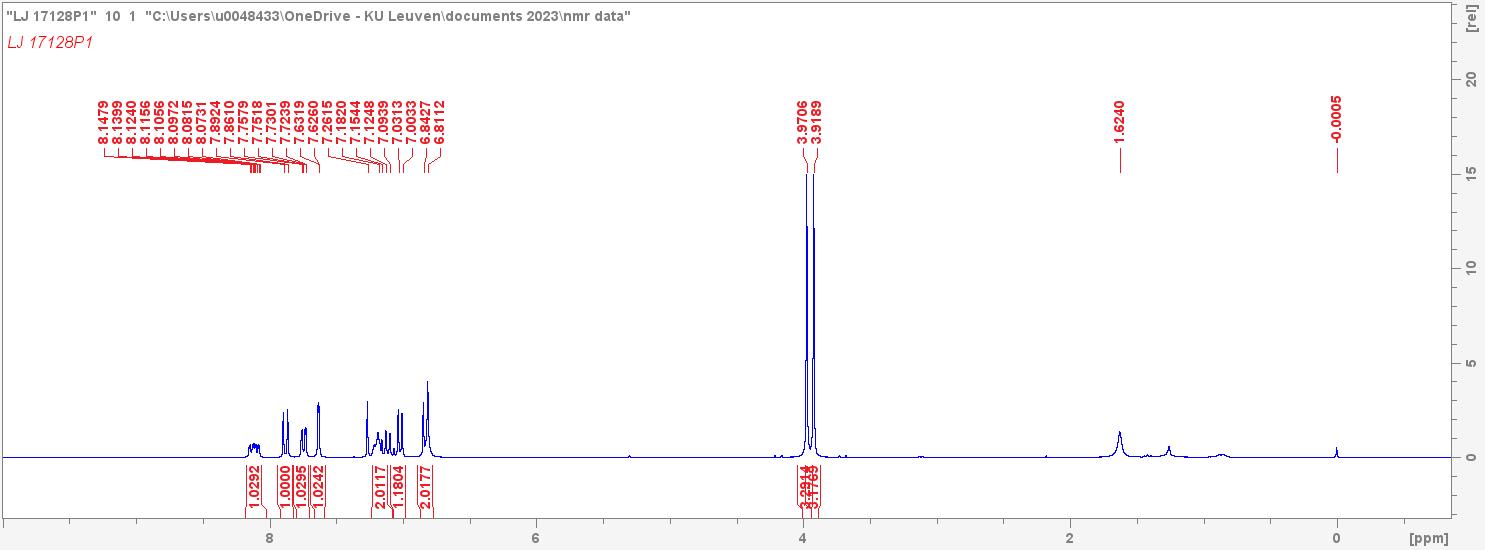


^13^C NMR spectrum of **17l**


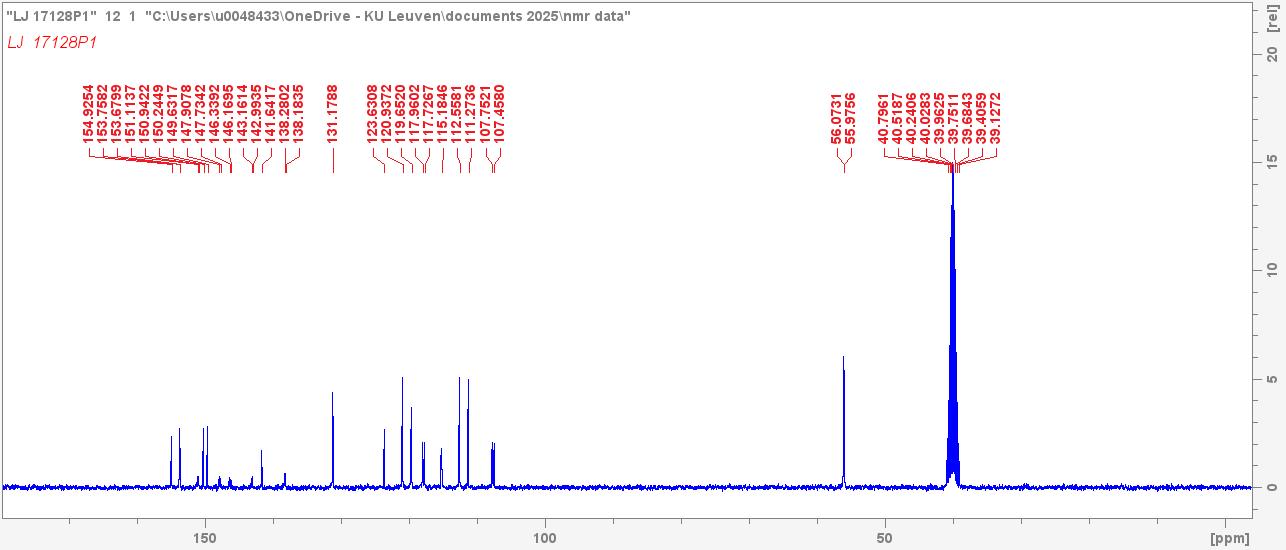


^1^H NMR spectrum of **17m**


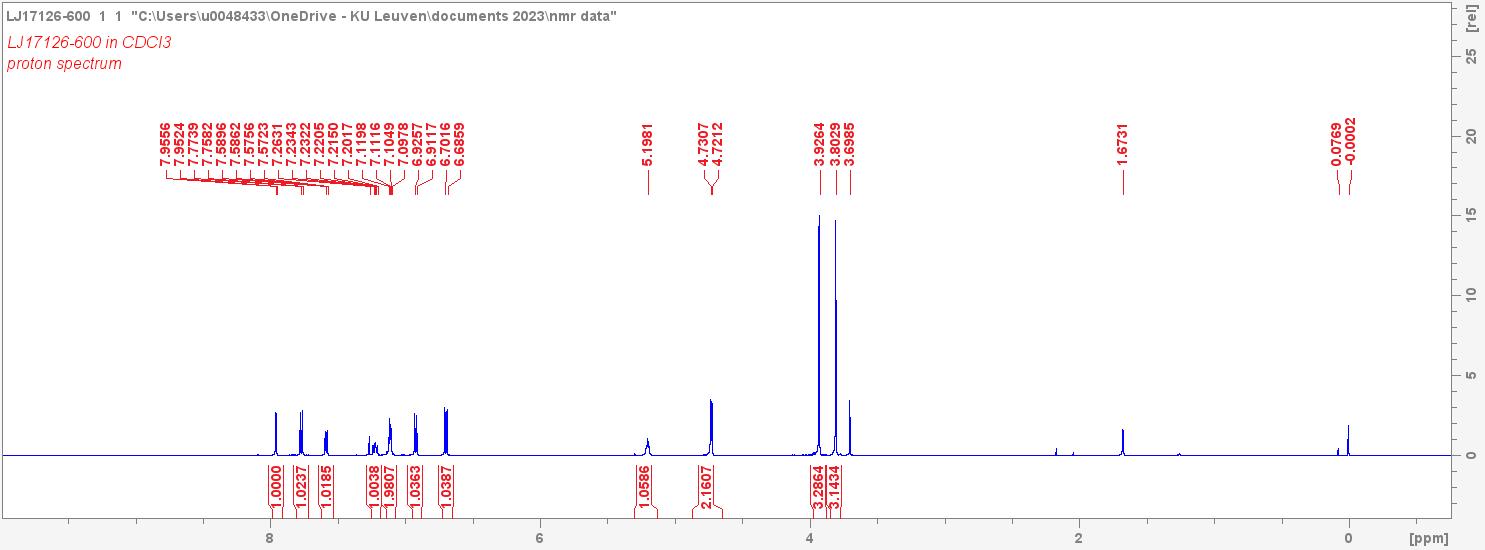


^13^C NMR spectrum of **17m**


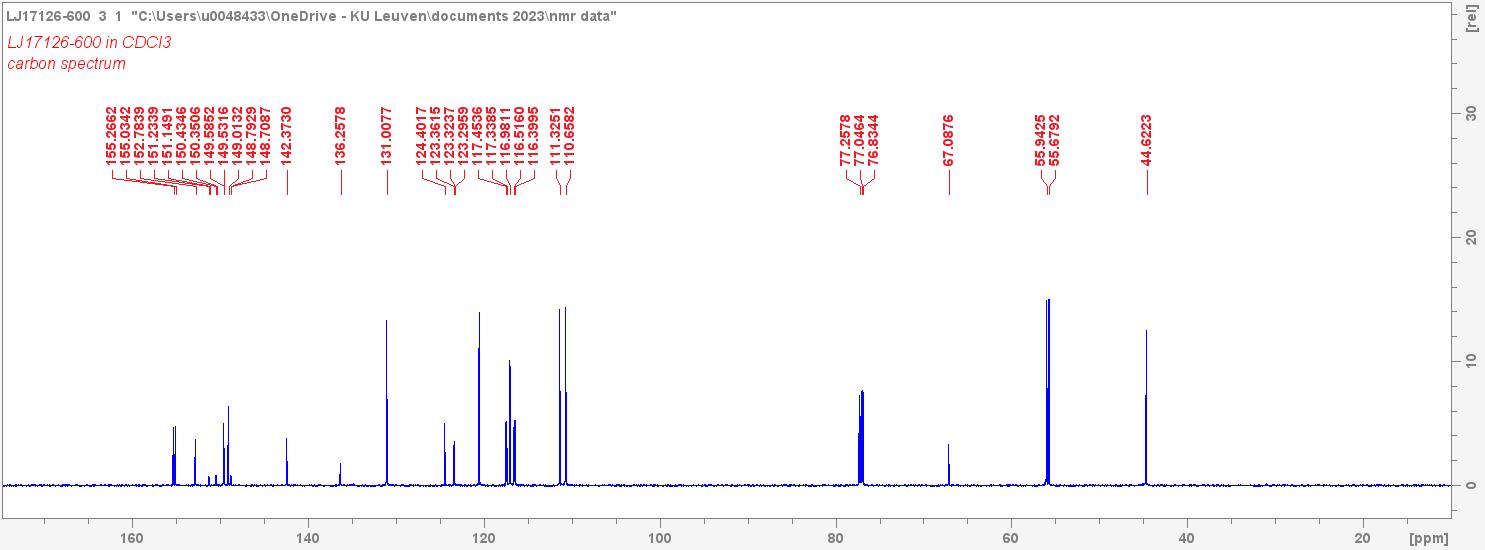


^1^H NMR spectrum of **17n**


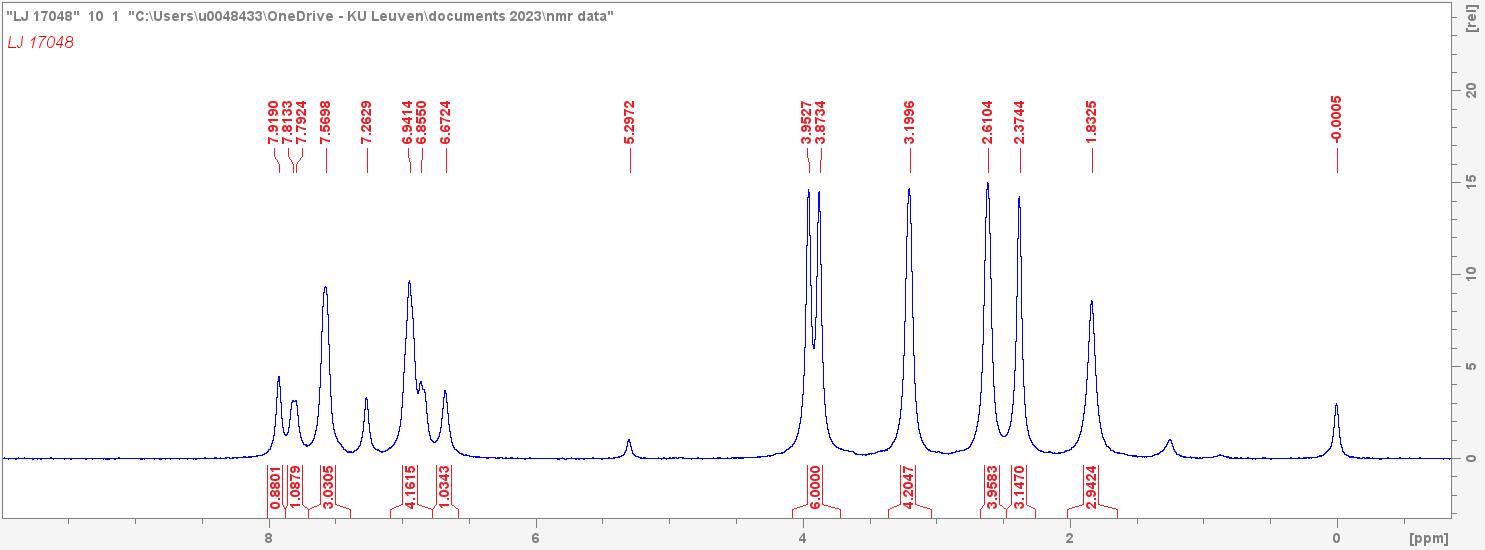


^13^C NMR spectrum of **17n**


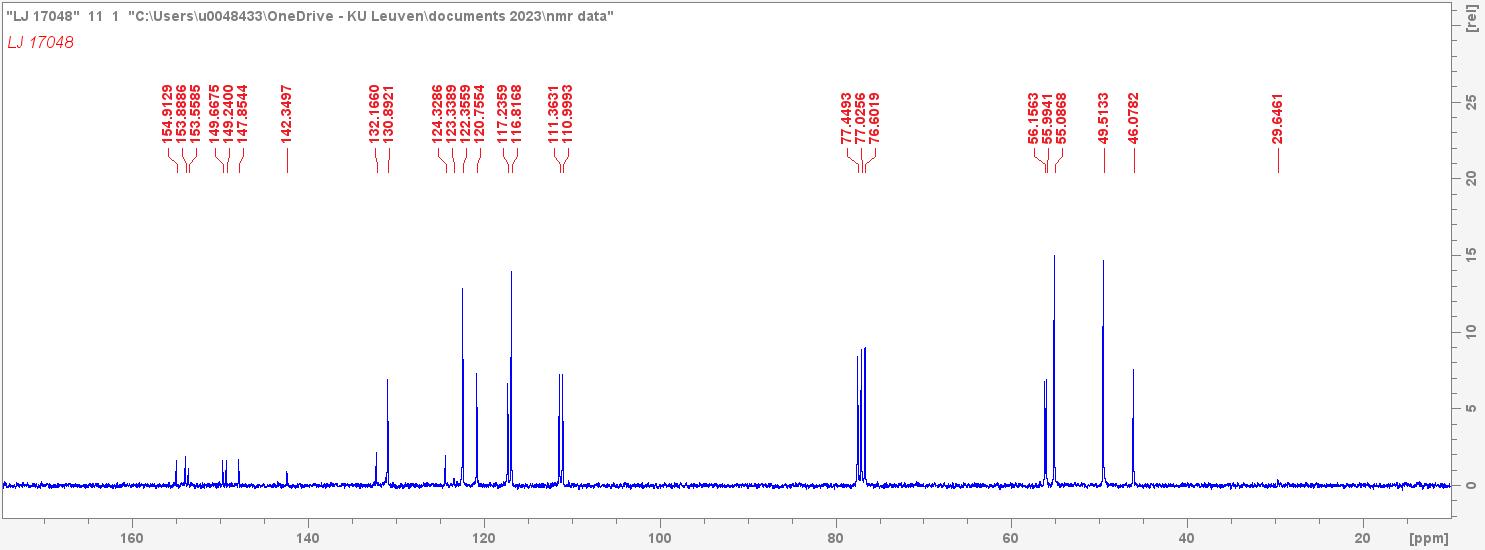


^1^H NMR spectrum of **17o**
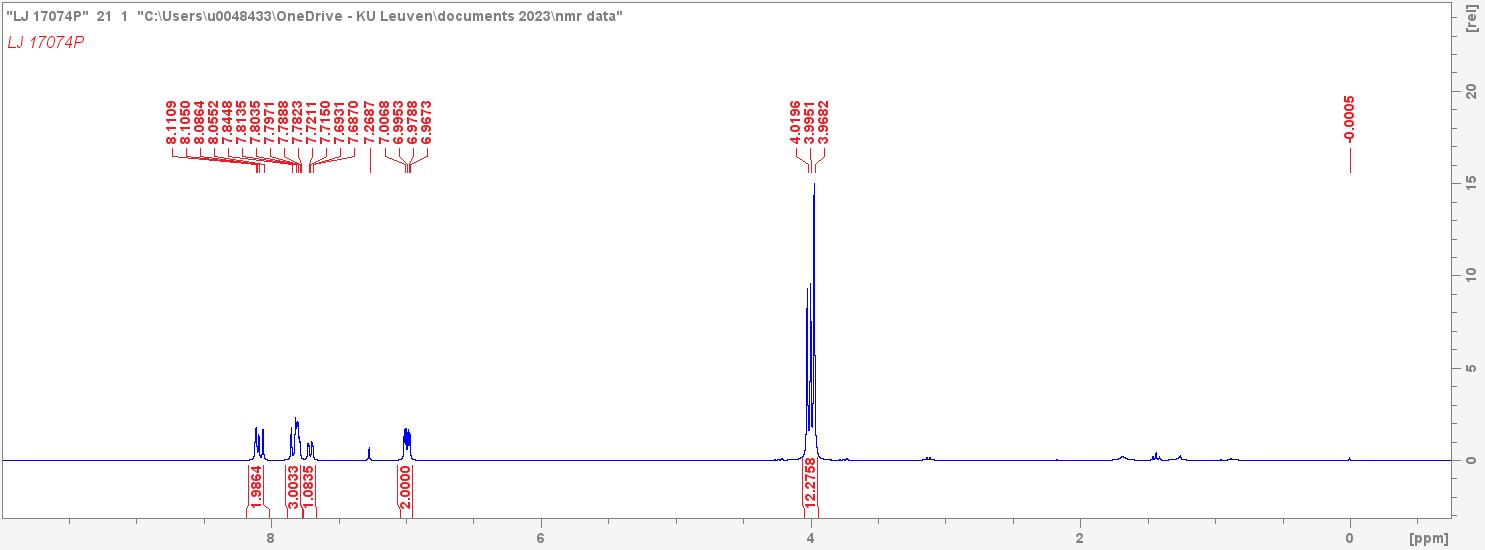


^13^C NMR spectrum of **17o**


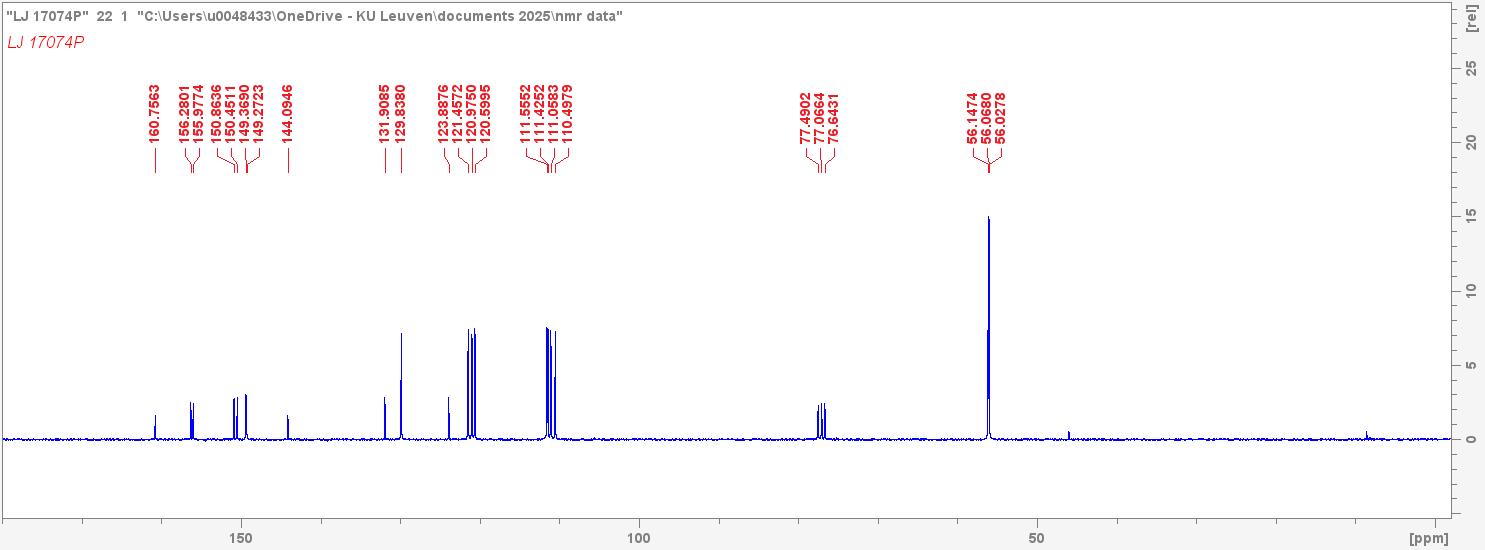


Residual activity for compound **16i** at 10 µM in duplicate for 70 kinases. Values are calculated as % of control. Staurosporine is positive control for the protein kinases; wortmannin is positive control for lipid kinases.
